# *N*-Heterocycles Scaffolds as *Quorum Sensing* Inhibitors. Design, Synthesis, Biological and Docking Studies

**DOI:** 10.3390/ijms21249512

**Published:** 2020-12-14

**Authors:** Alfredo Fuentes-Gutiérrez, Everardo Curiel-Quesada, José Correa-Basurto, Alberto Martínez-Muñoz, Alicia Reyes-Arellano

**Affiliations:** 1 Departamento de Química Orgánica, Escuela Nacional de Ciencias Biológicas del Instituto Politécnico Nacional (ENCB-IPN), Ciudad de México 11340, Mexico; alfredfg@hotmail.com; 2 Departamento de Bioquímica, Escuela Nacional de Ciencias Biológicas del Instituto Politécnico Nacional (ENCB-IPN), Ciudad de México 11340, Mexico; ecqmixcoacdf@gmail.com; 3 Laboratorio de Diseño y Desarrollo de Nuevos Fármacos e Innovación Biotecnológica, Sección de Estudios de Posgrado e Investigación, Escuela Superior de Medicina del Instituto Politécnico Nacional, Ciudad de México 11340, Mexico; corrjose@gmail.com (J.C.-B.); albert.wsker@gmail.com (A.M.-M.)

**Keywords:** heterocycles synthesis, new AHL bioisosteres, π–π interactions, *quorum sensing*

## Abstract

*Quorum sensing* is a communication system among bacteria to sense the proper time to express their virulence factors. *Quorum sensing* inhibition is a therapeutic strategy to block bacterial mechanisms of virulence. The aim of this study was to synthesize and evaluate new bioisosteres of *N*-acyl homoserine lactones as *Quorum sensing* inhibitors in *Chromobacterium violaceum* CV026 by quantifying the specific production of violacein. Five series of compounds with different heterocyclic scaffolds were synthesized in good yields: thiazoles, **16a**–**c**, thiazolines **17a**–**c**, benzimidazoles **18a**–**c**, pyridines **19a**–**c** and imidazolines **32a**–**c**. All 15 compounds showed activity as *Quorum sensing* inhibitors except **16a**. Compounds **16b**, **17a**–**c**, **18a**, **18c**, **19c** and **32b** exhibited activity at concentrations of 10 µM and 100 µM, highlighting the activity of benzimidazole **18a** (IC_50_ = 36.67 µM) and **32b** (IC_50_ = 85.03 µM). Pyridine **19c** displayed the best *quorum sensing* inhibition activity (IC_50_ = 9.66 µM). Molecular docking simulations were conducted for all test compounds on the *Chromobacterium violaceum* CviR protein to gain insight into the process of *quorum sensing* inhibition. The in-silico data reveal that all 15 the compounds have higher affinity for the protein than the native AHL ligand (**1**). A strong correlation was found between the theoretical and experimental results.

## 1. Introduction

*Quorum sensing* (QS), a mechanism of cell-to-cell communication in bacteria and fungi, involves self-produced chemical signals called autoinducers that function as semiochemicals [1]. Bacteria use this mechanism to communicate among themselves through the recognition and measurement of extracellular autoinducers, which accumulate in the local environment until reaching a certain level. At such a point, the bacterial population is sufficient to allow for group actions and therefore signaling pathways are activated and specific genes (some related to virulence factors) are transcribed [2,3,4,5]. 

Since the disruption of QS could plausibly attenuate or halt bacterial virulence and overcome bacterial resistance, it is an attractive target for drug discovery [6,7]. In Gram-negative bacteria the autoinducers for QS are *N*-acyl homoserine lactones (AHLs **1**). They are synthesized and released to the extracellular medium, diffuse freely through the bacterial membrane, and bind to specific cytoplasmic receptors. Once reaching a certain level, AHLs **1** promote specific gene expression responsible for bioluminescence [8], pigment production [9], toxin production [10], and the regulation of the expression of virulence factors such as biofilm formation [4,5,6]. It is particularly important to discover and implement an effective strategy for controlling Gram-negative bacteria due to their role in hospital-acquired infections and their increasing resistance to numerous drugs [6,11]. 

**Figure 1:**
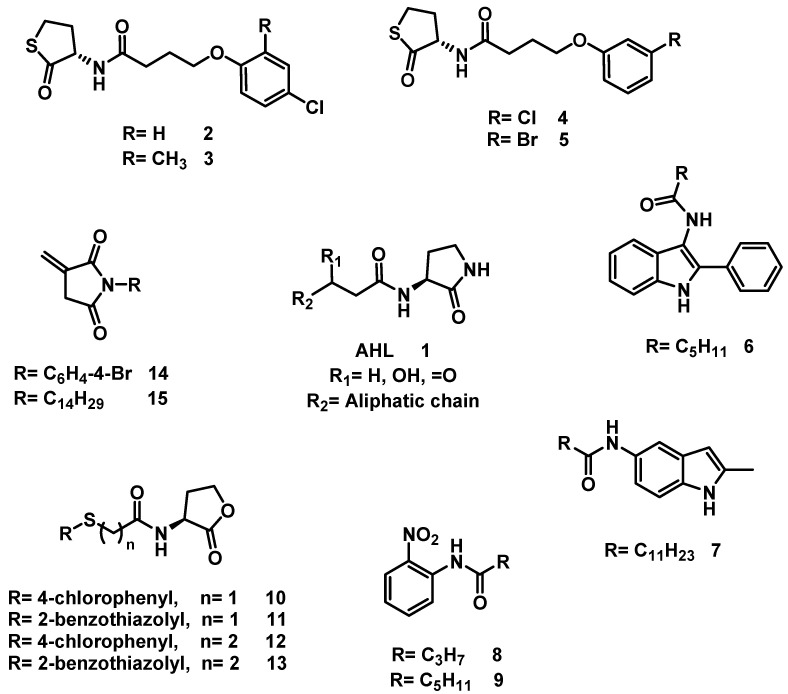
AHL and some known bioisosteres.

One of the most common strategies used to interfere with QS is the antagonism of AHL signaling by organic molecules [7]. Accordingly, researchers have developed bioisosteres of AHL (although they are not always designated as such), most of which are monovalent bioisosteres and act as antagonists [12,13]. Thus, the use of AHL as a lead compound has led to the development of a variety of QS inhibitors (QSIs) (Figure 1). The inhibition of QS has been achieved in *Chromobacterium violaceum* by a set of *N*-substituted homocysteine thiolactones (**2**–**5**) [14], in *Pseudomonas aeruginosa* by AHL bioisosteres based on indole (**6**–**7**) [15], in *Vibrio fischeri* by a *N*-(2-nitrophenyl)-amide scaffold (**8**–**9**) as an AHL bioisosteres [16], in *C. violaceum* and *P. aeruginosa* by a set of AHL bioisosteres (**10**–**13**) by modifying the acyl side chain [17], and in *P. aeruginosa* by a set of AHL bioisosteres based on the itaconimide scaffold (**14**–**15**) [18]. 

After synthesizing AHL bioisosteres, it is important to demonstrate their capacity to inhibit QS. An excellent model for this purpose is *Chromobacterium violaceum* due to it is a QS biosensor [19]. The aim of the present study was to synthesize new bioisosteres of AHL and evaluate them in *C. violaceum* CV026 as QSIs by quantifying the specific production of violacein. Five series of compounds were synthesized, each containing a distinct heterocyclic scaffold in its structure: thiazoles (**16a**–**c**), thiazolines (**17a**–**c**), benzimidazoles (**18a**–**c**), pyridines (**19a**–**c**) and imidazolines (**32a**–**c**) to be assayed experimentally. Finally, molecular docking simulations were conducted for all 15 test compounds on *C. violaceum* CviR protein to provide insights into the process of QS inhibition. 

## 2. Results and Discussion

### 2.1. Bioisosteres Design

**Figure 2:**
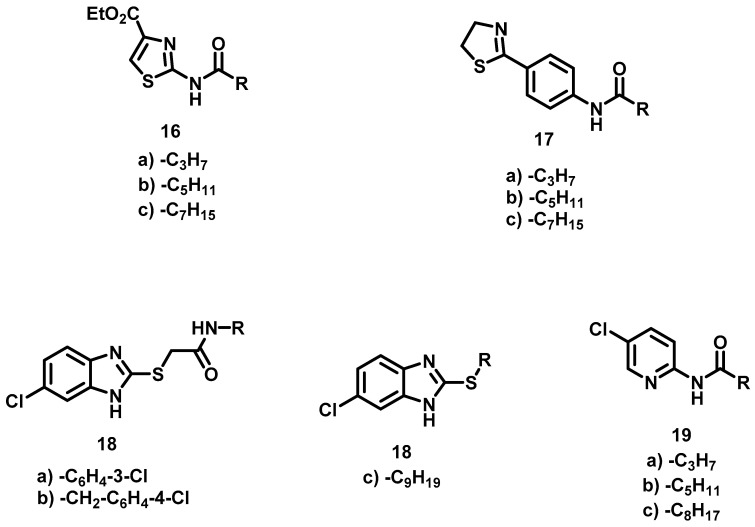
AHL bioisosteres design presently.

New non-classical bioisosteres [12] of AHL were designed by modifying hexanoyl homoserine lactone 1 (i.e., R_1_ = H, R_2_ = C_3_H_7_), an autoinducer of *Chromobacterium violaceum*. Specifically, the lactone ring 1 was bioisosterically replaced by heterocyclic rings of thiazoles (**16a**–**c**), thiazolines (**17a**–**c**), benzimidazoles (**18a**–**c**), pyridines (**19a**–**c**) and imidazolines (**32a**–**c**) (Figure 2). The lactone ring has been previously substituted by other heterocycles and even phenyl (Figure 1) [14,15,16,17,18]. Furthermore there are reports of its replacement with cyclopentane, cyclopentanol, cyclopentanone, oxazolidinone, benzothiazol and thiadiazol [13]. Our group reported a substitution with imidazoline and oxazoline in prior studies [20,21]. In the design of the present bioisosteres, the amido group and the lateral chain were conserved but the length of the latter varied. Additionally, an aromatic ring was included in all the molecules to promote π–π interactions with the receptor. Thus, five series of compounds emerged (Figure 2).

**Thiazoles 16a–c**. Although compounds 16 contain two structural elements (an amido group and a lateral chain) similar to hexanoyl homoserine lactone 1, they are non-classical bioisosteres [12], formed by exchanging one functional group with another (i.e., lactone with pyridine). The ester group could have been removed, but it was left in place to observe the effect.

**Thiazolines 17a–c**. The phenyl ring in these compounds serves both as a linker and the aromatic factor required in the design. 

**Benzimidazoles 18a–c**. The aim with this series was to combine a fragment-based QSI design [22] with a bioisosteric substitution of the lactone ring of AHL by a benzimidazole ring. Thioacetamide is a fragment in a compound reported to have good activity as a QSI [23], furthermore the thioacetamide group could be considered a retroisoster [12] of the amido group of AHL. In the case of **18a**–**b**, the lateral chain was replaced by a phenyl ring, which was 3-Cl-phenyl for **18a** and 4-Cl-benzyl for **18b**. The amido group of **18c** was substituted by a sulfur, and the nonyl chain was left in place since it has worked well in other bioisosteres [24].

**Pyridines 19a–c**. Although the pyridines contain two elements of AHL, they are regarded as non-classical bioisosteres because the pyridine ring is completely different from the lactone ring. Consequently, they are based on an exchange of one functional group for another [12]. 

### 2.2. Synthesis

#### 2.2.1. Synthesis of 2-Acylamino-thiazole-4-carboxylic Acid Ethyl Esters (**16a**–**c**)

**Scheme 1:**
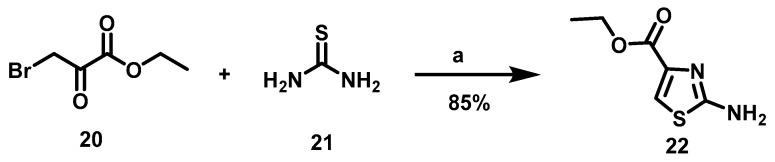
Synthetic route to obtain the *N*-(thiazol-2-yl)-amine (**22**). Reagents and conditions: a: EtOH, rt, 16 h.

The synthesis of **16a**–**c** began with the formation of amino thiazole **22**, utilizing thiourea and ethyl bromopyruvate as raw materials (Scheme 1).

To obtain 2-amine thiazol **22**, Hantzsch synthesis was carried out with an α-halo carbonyl compound, α-bromo ethyl pyruvate (**20**) and thiourea as raw materials and EtOH as solvent according to the methodology published by Kaushik [25]. The reaction mixture was stirred for 16 h at room temperature (rt), furnishing the desired product in good yields. (Scheme 1.) Subsequently, intermediary **22** was acylated with the corresponding carboxylic acid, a reaction described by various authors [26,27,28,29]. Such a reaction occurs in 2-amine thiazole with electron with drawing groups in position 4, for which heterogeneous yields have been documented in the literature. For instance, low yields (17–47%) were provided when Parish performed the acylation reaction with acyl chlorides in the presence of bases [26]. The acylation reaction has also been carried out with carboxylic acids and coupling reagents CDI or EDCI, affording moderate to good yields (54–69%) [27]. Considering, this background, distinct conditions were herein investigated but two procedures were followed: MW energy, as reported by You [28], and DCC as a coupling reagent with conventional heating as the energy source [29] (Table 1). 

Data in the literature [27] indicate that the acylation reaction of compounds like **22** depends on the coupling reagents, the solvent and the mixture of these two factors. Additionally, it is necessary to consider the low nucleophilicity of the amine group conjugated to an electron withdrawing group. When Toyoshima [30] carried out the synthesis of compounds **16**, the yields were lower than those found presently. In the acylation reaction of an amine, the excess concentration of the acid is important due autocatalysis [31]. Compounds **16** have been tested as antivirals and anti-tuberculosis drugs [30] but never as QSIs. 

#### 2.2.2. Synthesis of *N*-[4-(Thiazolin-2-yl)-phenyl]-amides (**17a**–**c**)

The synthetic pathway began with the reaction between 4-amino benzonitrile and cysteamine hydrochloride to prepare intermediate **25**.

To obtain **25**, 4-amine benzonitrile and cysteamine hydrochoride were reacted by using EtOH: H_2_O 1:1 as solvent (Table 2).

Cazin conditions [32] provided a low yield for **25** (Table 2, experiment 1), observing an incomplete reaction. Lengthening the reaction time (experiment 2) did not improve the outcome, nor did a greater amount of base (experiment 3), which caused the yield to drop sharply due to the hydrolysis of benzonitrile to carboxylic acid. The combination of a weaker base (K_2_CO_3_ instead of NaOH) and a higher reaction temperature, as described by Hintermann [33], gave better results (experiment 4). Since the raw material was detected in TLC, the reaction time was increased (experiment 5), leading to a high yield with the complete reaction of the raw material. 

**Figure 3:**
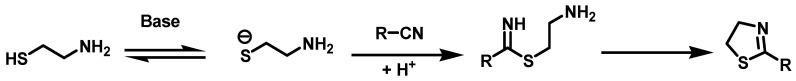
Transformation of benzonitriles to 2-thiazolines.

It is known that the transformation of benzonitrile to thiazoline was established in accordance with the computational studies by Mor and Cavalli [34], (Figure 3). 

The synthesis of **17** was completed by an acylation reaction of **25** with the corresponding carboxylic acids. Two possibilities were taken into account when planning the synthetic route of **17**: the acylation of amine thiazole **22** and the acylation of anilines, the latter frequently reported. The different conditions that have been adopted for the amine acylation reaction include carbodiimides as coupling reagents [26,27], MW and SiO_2_ [35], zeolitas as catalyst [31] and transamidation with zirconocene dichloride [36]. The methodology chosen, based on the acylation of thiazole **22** (Table 3), furnished the novel thiazolines **17a**–**c** in good yields.

#### 2.2.3. Synthesis of 6-Chloro-2-alkylsulfanyl-1H-benzimidazoles (**18a**–**c**)

**Scheme 2:**
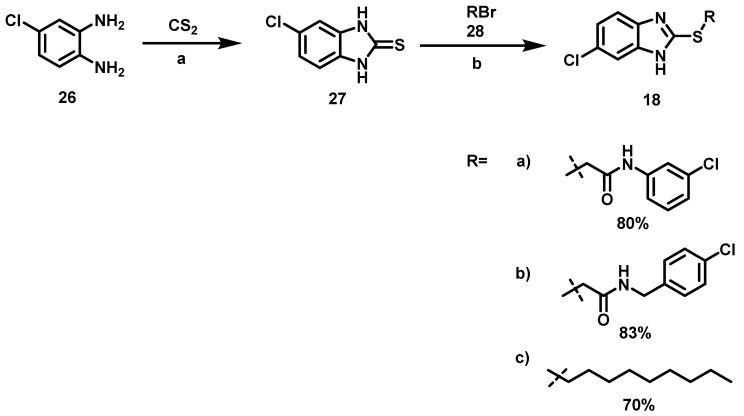
Synthetic procedure to obtain the 6-chloro-2-alkylsulfanyl-*1H*-benzimidazoles (**18a**–**c**). Reagents and conditions: a: potassium hydroxide, EtOH/H_2_O (7:3), 80 °C, 5 h; b: EtOH, 60 °C, 16 h.

The two-step synthetic procedure to obtain benzimidazoles **18a**–**c** (Scheme 2) began with the reaction between carbon disulfide and 4-chloro-1,2-phenylenediamine **26** to afford 5-chloro-benzimidazole-2-thione **27** [37] (Tabla 4). 

To prepare intermediary **27**, variations in the conditions were based on previous reports [37]. (Table 4).

**Scheme 3:**
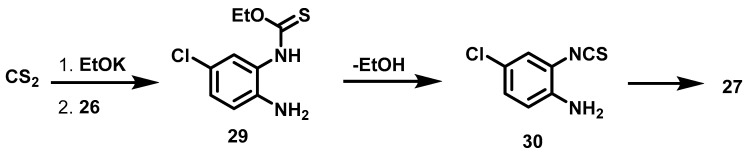
Synthetic route of thione **27** by transformation of 4-Cl phenylenediamine **26** with carbamothiate **29**.

Experiment 1 was carried out under the conditions described by Micheva [37], who obtained very good yields at 3 h of reaction. In our hands, however, the outcome was different, detecting the presence of phenylenediamine after 3 h (experiment 1). A change to MW energy (experiment 2) was also unsuccessful. By using conventional energy and increasing the reaction time from 3 to 5 h (experiment 3), the total consumption of raw material was observed and thione **27** was produced in a higher yield. In accordance with the work of Sartori [38], the proposed reaction sequence is the formation of ethyl(2-amino-5-chlorophenyl)carbamothioate **29** followed by its conversion into isothiocyanonitrile **30** and finally into thione **27** (Scheme 3).

**Scheme 4:**
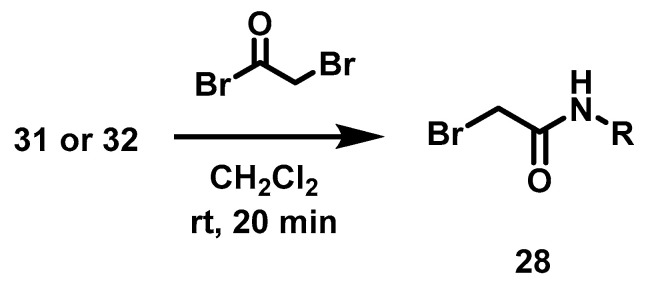
Synthesis of bromoacetamides **28**.

This intermediate **27** was then subjected to an acylation reaction [25] with the bromoacetamides **28a**, **28b** or alkylation with *n*-bromononane **28c**, to obtain **18a**–**c**, Scheme 3. Bromoacetamides **28a**–**b** were synthesized with 2-bromo acetyl bromide and 3-Cl aniline **31** or 4-Cl benzylamine **32** (Scheme 4). 

Finally **28** were reacted with **27** to achieve **18a**–**c**. Scheme 2. There are reports of benzimidazole acetamides as antidiabetic drugs [39], antimicrobial agents [40], and QSI in *P. aeruginosa* [41]. However, benzimidazoles **18** are new compounds that open a broad range of opportunities for research into QSIs.

#### 2.2.4. Synthesis of *N*-(5-Chloro-pyridin-2-yl)-amides (**19a**–**c**)

Compounds **19a**–**c** were synthesized by the acylation of 5-chloro-2-amino pyridine **33** under the same reaction conditions as for **17a**–**c** and **18a**–**c**. The *N-*(5-chloro-pyridin-2-yl)-amides **19** were generated in excellent yields (87–91%) (Table 5).

Although compounds **19** are known and their use has been reported [42,43], their possible application as QSIs is unexplored. Characterization of pyridines **19** was not found.

#### 2.2.5. Synthesis of *N*-Substituted-2-[4-(imidazolin-2-yl)-phenoxy]-acetamides (**32a**–**c**)

In addition to designing bioisosters **16**–**19**, three other novel imidazoline acetamides were synthesized (**32a**–**c**) because of the similarity of their structure to that of imidazoline, the QS inhibitory activity of which is well recognized [20]. Moreover, their structural elements coincide with those considered presently. In a previous theoretical study, the ΔG of the complexes formed by **32a**–**c** and the CviR protein were calculated, finding much lower values than for the complex of AHL with the same protein [44].

**Scheme 5:**
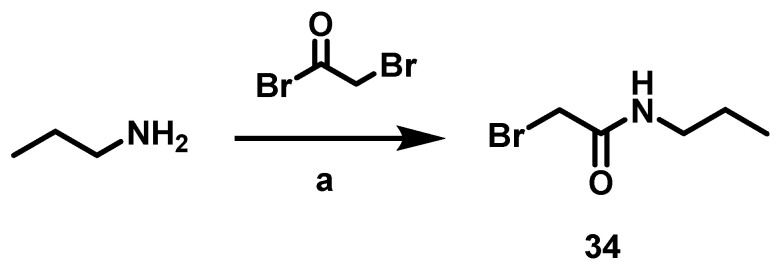
Procedure to synthesize α-bromo-*N*-propylacetamide **34**. Conditiones: a. Amine (1 eq), bromoacetyl bromide (1.2 eq), CH_2_Cl_2_ (15 mL), rt, 20 min.

Based on the method described by our group [20], imidazolines **32** were synthesized with different substituents on the amide group. Obtaining compounds **32** began with the synthesis of the aldehydes **35**, for this it was necessary to obtain acetamide **34** (Scheme 5).

To prepare aldehydes **35**, a previous report was considered [20] (Table 6).

**Scheme 6:**
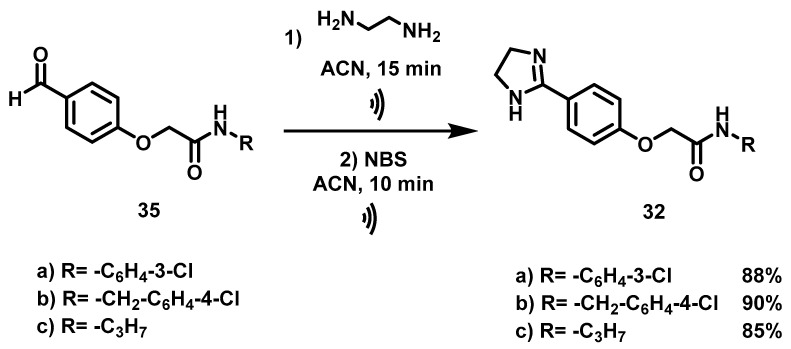
Synthetic procedure to obtain the imidazoline acetamides (**32a**–**c**). Reagents and conditions: (**a**) CH_2_Cl_2_, rt, 20 min; (**b**) DBU, ACN, 70 °C, 8 h; (**c**) ethylenediamine, ACN, 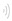, 15 min; then NBS, ACN, 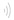, 10 min.

Compared to the report on the preparation of aldehyde **35a**, a shorter time was herein achieved (Table 6) by using 2-bromoacetyl bromide rather than the previously employed α-bromoacetic acid and DCC [20]. Compounds **35** were subjected to cyclization with ethylenediamine and subsequent oxidation with NBS under ultrasound irradiation, as reported by Torres [29]. The corresponding imidazolines **32a**–**c** were provided in good yields (85–90%) (Scheme 6).

### 2.3. Characterization of Compounds

All compounds were characterized by spectroscopic methods utilizing IR, NMR, and DIP-MS. Spectra were assigned with the help of heteronuclear correlation (gHSQC, gHMBC) and in some cases with homonuclear correlation (gCOSY). Mass spectra were recorded with positive and negative ions. Spectra for all compounds are attached in the Supplementary Materials (Figures S1–S104).

## 3. Evaluation of QS Inhibition on *Chromobacterium violaceum* CV026

**Figure 4:**
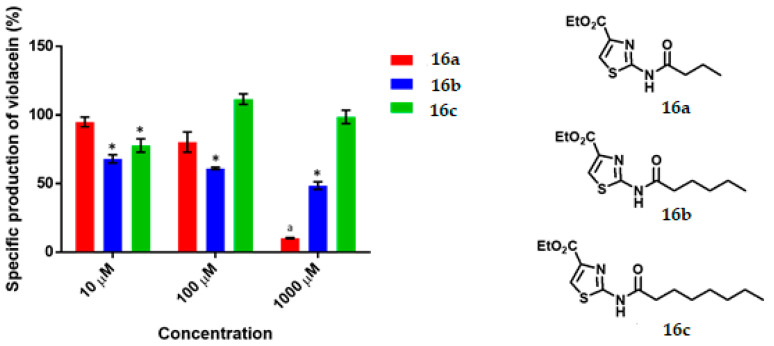
Specific production of violacein by *Chromobacterium violaceum* CV026 in the presence of the thiazoles (**16a**–**c**) at 10, 100 and 1000 μM. Data are expressed as a percentage of the specific production of violacein (mean ± SEM) and normalized by considering the value without the addition of a compound as 100%. Significance was confirmed by the Student’s *t*-test with an accuracy of * *p* < 0.05 at the marked bars. ^a^ Denotes an antimicrobial effect of the compound. Experiments were performed with *n* = 6.

**Figure 5:**
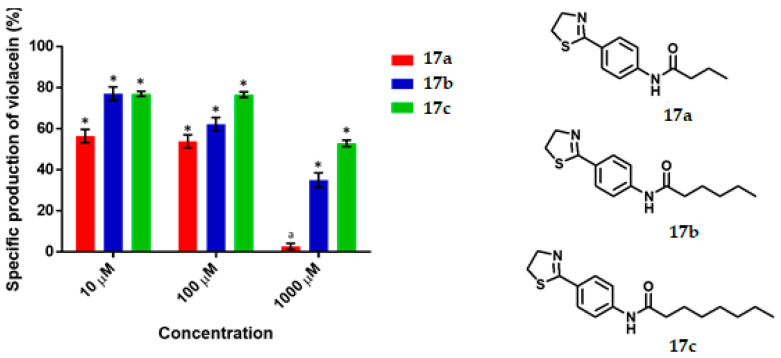
Violacein specific production by *Chromobacterium violaceum* CV026 in the presence of the synthesized thiazolines at 10, 100 and 1000 μM concentrations. Data are expreseed as the percentage of the specific production of violacein (mean ± SEM) and normalized on the violacein production without compound addition and this was considered as 100%. Significance was confirmed by the Student’s *t*-test with an accuracy of * *p* < 0.05 at the marked bars; ^a^ represents antimicrobial effect of the tested compound. Experiments were performed with *n* = 6.

**Figure 6:**
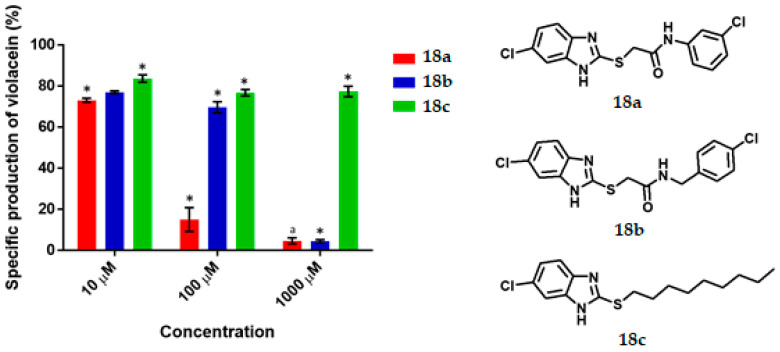
Violacein specific production by *Chromobacterium violaceum* CV026 in the presence of the synthesized benzimidazoles at 10, 100 and 1000 μM concentrations. Data are presented as percentages of violacein specific production (mean ± SEM) and normalized on the violacein production without compound addition and this was considered as 100%. Significance was confirmed by the Student´s *t*-test with an accuracy of * *p* < 0.05 at the labelled bars; ^a^ Indicates an antimicrobial effect of the compound. Experiments were performed with *n* = 6.

**Figure 7:**
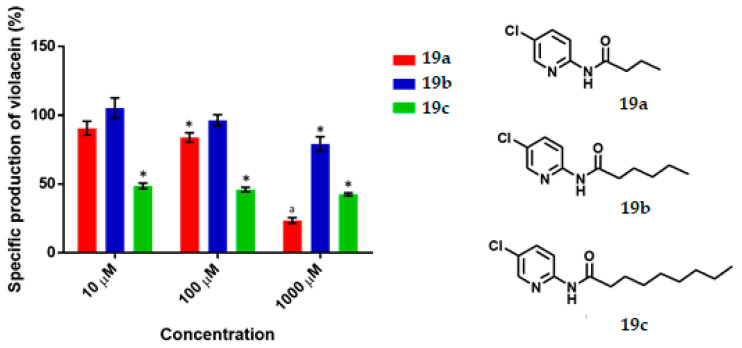
The specific production of violacein by *Chromobacterium violaceum* CV026 in the presence of the synthesized compounds (**19a**–**c**) at 10, 100 and 1000 μM. Data are expressed as a percentage of specific production of violacein (mean ± SEM), normalized on the violacein production without compound addition and this considered as 100%. Significance was confirmed by the Student’s *t*-test with an accuracy of * *p* < 0.05 at the marked bars. ^a^ Indicates an antimicrobial effect of the tested compound. Experiments were performed with *n* = 6.

**Figure 8:**
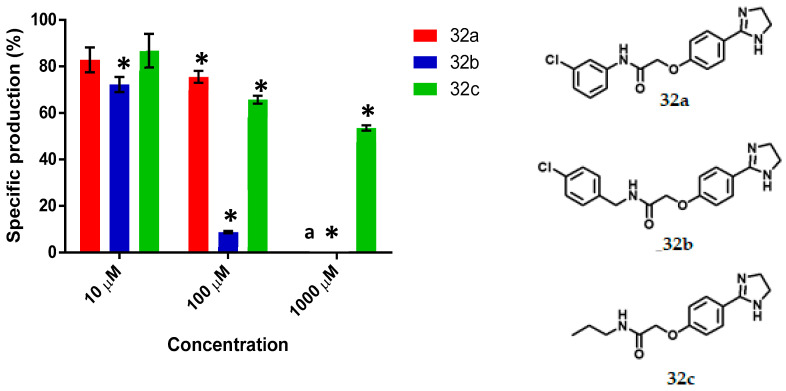
The specific production of violacein by *Chromobacterium violaceum* CV026 in the presence of the synthesized imidazolines (**32a**–**c**) at 10, 100 and 1000 μM concentrations. Data are presented as percentages of violacein specific production (mean ± SEM), and normalized on the violacein production without compound addition and this was considered as 100%. Significance was confirmed by the Student’s *t*-test with an accuracy of * *p* < 0.05 at the marked bars. ^a^ Denotes antimicrobial effect of the tested compound. Experiments were performed with *n* = 6.

The synthesized compounds were added (at 10, 100 and 1000 μM) to the bacteria (OD600 of 0.12) in thioglycolate broth supplemented with C6-AHL, followed by incubation for 16 h. The determination of the specific production of violacein, calculated as the ratio of detectable pigment (OD577) per amount of bacteria (OD720), demonstrated the existence of QS inhibitory activity for almost all test compounds. (Figure 4, Figure 5, Figure 6, Figure 7 and Figure 8). There is a clear difference between *quorum sensing* and antibacterial activity. In this research, growth inhibition by the evaluated heterocycles results in a decrease in OD720. While this decrease was statistically significant, taking cultures grown in the absence of inhibitors as a basis of comparison, this is denoted with a letter ‘a’ on the top of the corresponding bar and stated as ‘a’ the footnote of the figure.

### 3.1. Thiazoles

The QS inhibitory activity of the series of thiazoles is illustrated in Figure 4. No such effect was found for **16a** at any concentration. Thiazole **16b** at 10, 100 and 1000 μM showed low-moderate QS inhibitory activity (32%, 39% and 51%, respectively) with an IC_50_ = 925.0 µM. The thiazole **16c** was QSI only at 10 μM, causing limited inhibition (22%). 

### 3.2. Thiazolines

The results for thiazoline series are shown in Figure 5. Thiazoline **17a** displayed a moderate QS inhibition at 10 and 100 μM being of 44% and 47% respectively, while at 1000 μM it behaved as antimicrobial. Thiazoline **17b** exhibited a limited to good inhibition at 10, 100 and 1000 μM being of 23%, 37% and 65% respectively, in addition **17b** gave an IC_50_ value of 517.86 µM. Compound **17c** reduced violacein production 23%, 24% and 48% at 10, 100 and 1000 μM respectively. 

### 3.3. Benzimidazoles

For the benzimidazole series (Figure 6), **18a** gave 27% and 85% QS inhibition at 10 and 100 μM respectively, presented a value of IC_50_ of 36.67 µM, and behaved as an antimicrobial at 1000 μM. For **18b** at 100 and 1000 μM, good and excellent activity as a QSI was found (31% and 95%, respectively). The IC_50_ value of **18b** is 376.92 µM. For **18c** the inhibitory effect was 17%, 24% and 23% at 10, 100 and 1000 μM, respectively.

Activity as QSI of benzimidazole **18a** (85% QS inhibition at 100 µM, IC_50_ of 36.67 µM) is comparable than 2-[4-(hexyloxy)phenyl]-4,5-dihidro-1*H*-imidazole (65% QS inhibition at 90 µM, IC_50_ = 56.38 µM) [21], more active than 4-nitro-pyridine-*N*-oxide (active at 100 µM), the best synthetic *quorum sensing* inhibitor (QSI) in a screening of 27 synthetic compounds and 27 natural extracts [11,45]. However benzimidazole **18a** is not QSI like 2-(4′-chlorophenoxy)-*N*-butanoyl homoserine lactone (IC_50_ = 377 nM) [7]. 

### 3.4. Pyridines

Pyridine **19a** exhibited QS inhibition activity (17%) at 100 μM, while at 1000 μM it behaved as an antimicrobial. Compound **19b** was active against QS only at 1000 μM, eliciting a 21% inhibition. In the case of **19c**, all three concentrations (10, 100 and 1000 μM) showed an QS inhibitory effect (52%, 55% and 58%, respectively). (Figure 7). According to the experimental data, the IC_50_ was calculated to be 9.66 µM. Pyridine **19c** is the best compound of all those tested in this work, its activity as QSI is comparable to **2** and **3** (IC_50_ = 1.1 µM and 2.2 µM respectively) [7]. It is more active than other synthesized inhibitors like 4-nitro-pyridine-*N*-oxide (active at 100 µM) [11,45] or 2-[4′-(pentyloxy)phenyl]-4,5-dihydro-1*H*-imidazole (IC_50_ = 56.38 µM) but is not better than 4-[(4′-chorophenoxy)-*N*-(2-oxotetrahydrofuran-3-yl)]butanamide (IC_50_ = 377 nM) [7] nor better than *N*-[4′-(4,5-dihydro-1*H*-imidazol-2-yl)phenyl]nonamide (activity as QSI from 1 nM) [24]. 

### 3.5. Imidazolines

For the series of imidazolines (Figure 8), **32a** and **32c** at 100 μM exhibited 24% and 34% activity as QSI, respectively. Imidazoline **32b** was QSI at all concentrations and displayed IC_50_ = 65.09 µM. The QS inhibition activity of **32b** is comparable to other synthesized bioisosteres [21]. Imidazoline **32c** at 1000 μM afforded 46% activity and compound **32a** behaved as an antimicrobial at the latter concentration. Compound **32b** is one of the top three in the series of compounds synthesized in this work, its QS inhibition activity is lower than that of ethyl (4-fluorobenzoyl) acetate (IC_50_ = 23 µM) the best QSI of a library with 26 compounds [46], but it is similar to 2-[4′-(pentyloxy)phenyl]-4,5-dihydro-1*H*-imidazole (IC_50_ = 56.38 µM) and more active than QSI 4-nitro-pyridine-*N*-oxide (active at 100 µM) [11,45].

## 4. Docking Results

Molecular docking was carried out to provide insight into the non-bonding interactions of the test compounds with the CviR protein (PDB code: 3QP6). The binding modes were observed and the affinity energy of each compound was calculated and expressed as ∆G (kcal/mol). The Docking simulations were performed with AutoDock 4.2, utilizing the blind docking method. The grid box size was adjusted to 126 Å^3^ with a grid spacing of 0.375 Å^3^. The Lamarckian genetic algorithm was employed with a randomized initial population of 100 individuals and a maximum number of energy evaluations of 1 × 10^7^. [47] The docking results were analyzed and visualized on AutoDockTools and PyMOL [48], represented as 3D plot conformations. The affinity energies of the evaluated compounds are shown in Table 7. 

Molecular Docking of compounds **32a**–**c** were performed by us in a previous study [44] obtaining the following results: **32a**, ΔG, = −11.39 kcal/mol; **32b**, ΔG = −8.52 kcal/mol y 32c, ΔG = −10.42 and AHL (1), ΔG = −7.26 kcal/mol. 

The theoretical calculations reveal that all tested compounds had greater affinity for the protein than the native ligand C6-AHL (1, R_1_ = H, R^2^ = C_3_H_7_). In addition, a strong correlation exists between the in silico and experimental results. For example, **17b**–**c**, **18a**–**c** and **19c** displayed good experimental inhibition and had the greatest affinity energies in silico.

**Figure 9:**
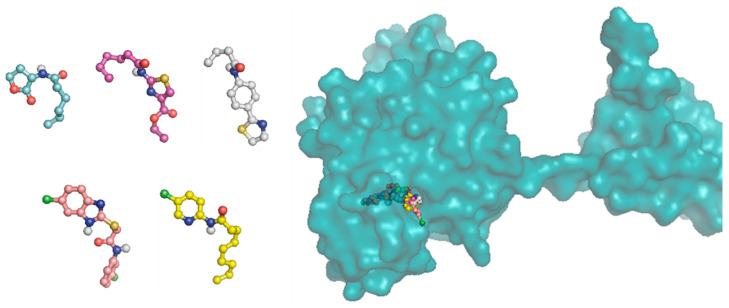
3 D plot conformation of molecular docking of ligands C6-AHL, **16b**, **17a**, **18a** and **19c** in the active site of the CviR protein.

The amino acids residues of the protein involved in the interaction with the C6-AHL, the native ligand, also participate in the binding of the most test compounds. There are hydrogen bond interactions of the polar groups of the compounds with the TRP84, ASP97 and SER155 residues. Additionally, there are π–π interactions of the phenyl ring on the thiazolines **17a** and **17b** with the phenyl group of TYR88, of the benzimidazoles **18a**–**b** with TYR80, and of the pyridine ring of compound **19c** with TRP111. Morever, hydrophobic interactions took place with most of the residues at the binding site, including ILE57, VAL59, MET72, LEU85, ILE99, PHE126, ALA130 and MET135. (Figure 9). 

**Figure 10:**
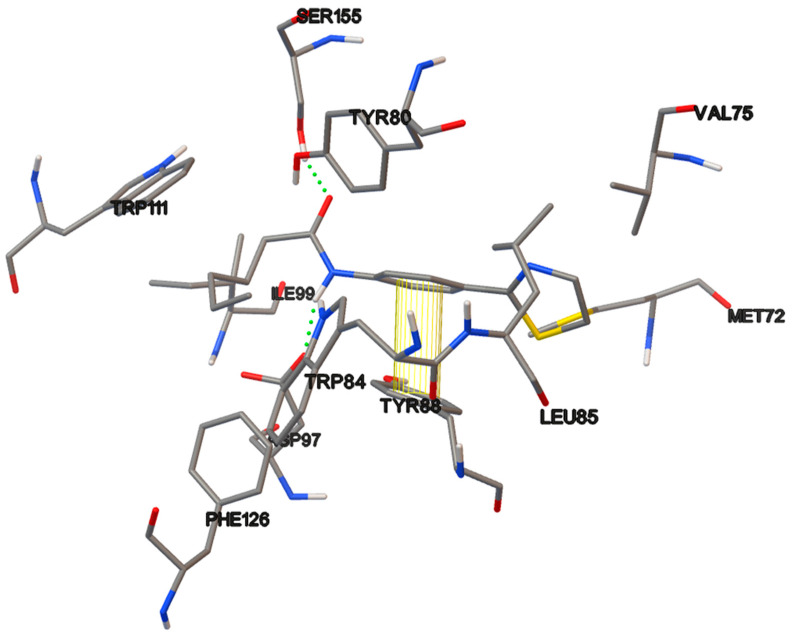
3 D plot conformation of non-covalent interactions in the ligand-protein complex containing compound **17b**.

**Figure 11:**
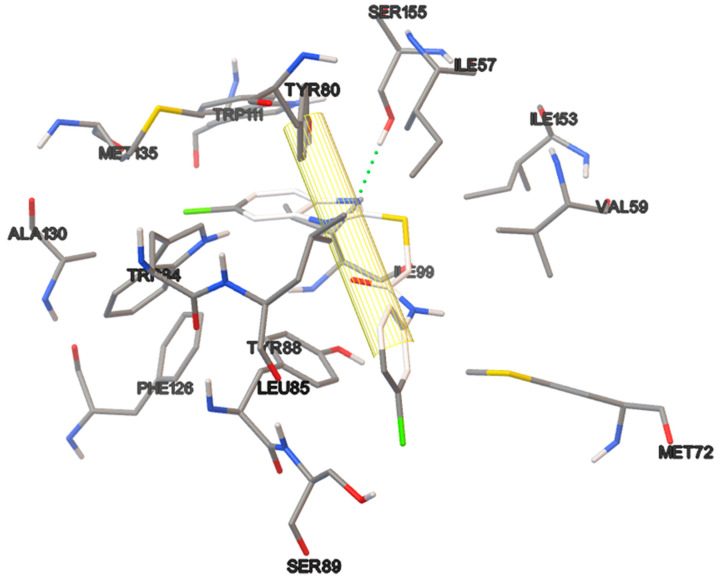
Non-covalent interactions of the ligand-protein complex containing **18a**.

**Figure 12:**
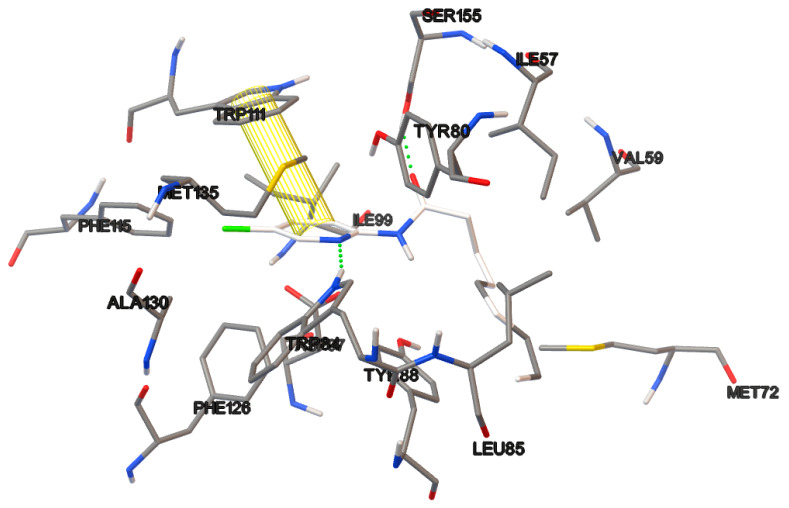
Non-covalent interactions of the ligand-protein complex containing compound **19c**.

The non-covalent interactions of the most active ligands evaluated (**17b**, **18a** and **19c**) are observed in the Figure 10, Figure 11 and Figure 12. The π–π interactions in the protein-compound complexes highlight the relevance of the phenyl moiety in the chemical structure of the test compounds. Other non-covalent interactions are involved in these complexes, such as hydrophobic and hydrogen bond interactions. 

## 5. Structure-Activity Relationship

The elements present in the bioisosteres of this work were a heterocycle, the amide group and the side chain. In one of the 5 bioisosteres, a phenyl ring was introduced as a linker between the amide and the heterocycle (**17**). 

The chain length is important for the activity, not because of the interactions that it establishes, since they are all hydrophobic, but rather because of the size that permits other interactions; thus the compound **16a** that has an aromatic ring, the thiazol, and the amide group but a chain of only 3C, did not display activity at any concentration. According to the Docking analysis this compound does not satisfy the size required to give all interactions like its homologous **16b** and **16c**. The latter compounds showed hydrogen bonding between the C=O of the amido group and TYR 80 and SER 155.

**Figure 13:**
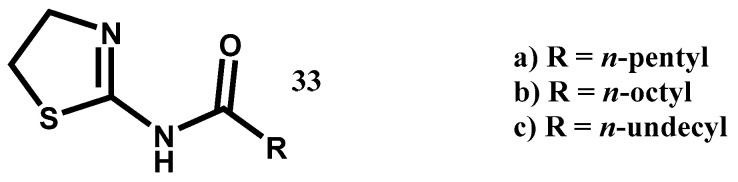
Inactive thiazolines as QSI.

Thiazolines **17** contain a thiazoline ring, a phenyl as a connector, the amido group, and propyl, pentyl and heptyl chains. They exhibited some degree of QS inhibition, except for **17a** at 1000 µM, showing antimicrobial activity. In an earlier work, our group synthesized thiazolines, **33a**–**c**, (Figure 13) like the thiazolines **17** but without the phenyl conector; all those thiazolines were inactive as QSI [29,49]. Thiazoline **17b** afforded a low activity as QSI, 23% and 37% at 10 and 100 µM, respectively, as well as 65% at 1000 µM. The phenyl linker enables a greater number of interactions than the aliphatic chains alone, and increases the length of the compound. The π–π interactions of the complex formed by compound **17b** and tyrosine 88 at the active site the CviR protein is illustrated in Figure 12. 

All benzimidazoles (**18**) with a thiol functionality display good QSI activity, **18a** and **18b**, so the compound **18a** exhibited 85% QSI at 100 µM and **18b** 95% at 1000 µM. Benzimidazol **18c** displayed only 17, 24 and 23% inhibitory activity at 10, 100 and 1000 µM, respectively. According to the Docking simulations, the benzimidazole ring occupies the space of the lactone and the amide of AHL, while thioacetamide engages the space of the aliphatic chain of AHL. The interactions of the protein with the benzimidazole rings were similar to those found with the lactone ring and the amido group of AHL Moreover, the phenyl ring of **18a** y **18b** presents π–π interactions with the receptor. Conversely, no π–π interactions were observed for **18c** because there is no phenyl ring in its structure, and the benzimidazol ring, which could give these interactions, establishes the interactions mentioned above. Benzimidazoles **18a**–**b** do not contain an aliphatic chain; but have a thioacetamide group, giving them greater length and flexibility. 

Since **18a** is QSI at 100 µM and antimicrobial at 1000 µM, it is possible to find a concentration that is able to restore sensitivity to an antibiotic. It is known that the QSI addition to an antibiotic can restore the sensitivity to the drug and increase its potency [11,50], therefore we consider that benzimidazol **18a** that behaves as very good QSI at one concentration and antimicrobial at another is very promising.

Pyridine **19a** with a propyl chain exhibited little QSI activity, 17% at 100µM and behaved as an antimicrobial at 1000 µM suggesting that chain was too short to properly couple to the protein. Among the pyridines, the best activity as a QSI was produced by **19c**, the compound with a nonyl chain. Its inhibitory activity was surprisingly similar at all three concentrations (52, 55 and 58% at 10, 100 and 1000 µM, respectively), which has no apparent explanation. Pyridines **19a** and **19b** generate the same hydrogen bond as AHL between NH and Asp 97. With **19b**, there is in an additional hydrogen bond of C=O of the amide group with SER 155 and TYR 80. It was observed that **19c** is rotated 180° in relation to AHL and forms two hydrogen bonds: one between the compound **19c** carbonyl and SER 155, and the other between N and TRP 84. All imidazolines displayed activity as QSI at 100 µM and 1000 µM, although only **32b** (at 1000 µM) showed good activity. Compound **32c**, containing a propyl lateral chain, gave rise to 34 and 46% QSI at 100 and 1000 µM respectively. 

## 6. Materials and Methods

### 6.1. General

All reagents and solvents were purchased from Sigma Aldrich (Toluca, Mexico) and used without further purification. Reactions were monitored by thin-layer chromatography (TLC) on Merck F253 silica gel aluminum sheets, and spots were revealed with ultraviolet (UV) light (254 nm). NMR experiments were carried out in Varian NMR System (500 MHz and 125 MHz), Varian Mercury (300 MHz and 75 MHz) and Bruker ASCEND (600 MHz and 150 MHz). ^1^H NMR and ^13^C spectra were assigned with the help of 2-D experiments (gHSQC and gHMBC). The chemical shifts (δ) are given in ppm. Mass spectra (MS) were recorded on a Bruker Amazon Speed (ESI). Infrared (IR) spectra were obtained on a Perkin Elmer FT-IR Spectrum 2000 spectrometer from the ENCB-IPN spectroscopy instrumentation center. Melting points were determined on an Electrothermal MEL-TEMP apparatus and are uncorrected. Microwave reactions were accomplished on a CEM Discovery SP apparatus. 

### 6.2. Procedure for the Synthesis of Ethyl 2-Aminothiazol-4-carboxylate, ***22***

Ethyl bromine pyruvate (7.69 mmol, 1 eq), thiourea (7.69 mmol, 1 eq) and 15 mL of EtOH were added to a flask equipped with a magnetic stirring bar and left to reaction mixture at rt for 16 h. At the end of this period, the solvent was evaporated under reduced pressure, followed by the addition of 40 mL of 20% aqueous potassium carbonate to the residue. The suspension formed was maintained under constant stirring for 30 min before subjecting it to vacuum filtration. The solid retained on the filter paper was washed with 40 mL of distilled water in two 20 mL portions and oven-dried at 40 °C. 

Ethyl 2-Aminothiazol-4-Carboxylate, **22**

Yield: 85%. White solid. ^1^H NMR (300 MHz, DMSO-*d*_6_) δ: 7.44 (s, 1H, H-5), 7.24 (s, 2H, NH), 4.17 (q, *J* = 7.10 Hz, 2H, H-8), 1.22 (t, *J* = 7.10 Hz, 3H, H-9). ^13^C NMR (75 MHz, DMSO-*d*_6_) δ 168.6 (C-2), 161.5 (C-6), 142.6 (C-4), 117.4 (C-5), 60.6 (C-8), 14.6 (C-9). DIP-MS (ESI; M + Na) *m*/*z* (calculated): 195.01, *m*/*z* (measured): 195.00.

### 6.3. Synthesis of Ethyl 2-Acylamidothiazol-4-carboxylate, ***16***

2-Amino thiazole (1.74 mmol, 1 eq) and 2 mL of carboxylic acid (butanoic acid at 150 °C, hexanoic acid at 150 °C, octanoic acid at 180 °C) were added to a reaction vial equipped with magnetic stirring, the reaction mixture was warmed for 90 min. It was then transferred to an aqueous solution of 20% potassium carbonate (40 mL) and allowed the mixture to stir for 30 min. Subsequently, the suspension was vacuum filtered and the solid obtained washed with distilled water (2 × 20 mL) and oven-dried at 40 °C.

Ethyl 2-Butyramidothiazol-4-Carboxylate, **16a**

Yield: 82%. Brownish solid; m.p. 145–148 °C. Lit. [30] 150–151 °C *R*_f_ = 0.6 (AcOEt). IR (CH_2_Cl_2_) $\bar{\upsilon }$ = 3256.8, 1720.1, 1690.4, 1546.0, 1215.1 cm^−1^. ^1^H NMR (300 MHz, CDCl_3_) δ: 7.81 (s, 1H, H-5′), 4.35 (q, *J* = 7.12 Hz, 2H, H-3″), 2.43 (t, *J* = 7.47 Hz, 2H, H-2), 1.70 (m, 2H, H-3), 1.35 (t, *J =* 7.12 Hz, 3H, H-4″), 0.94 (t, *J =* 7.47 Hz, 3H, H-4). ^13^C NMR (75 MHz, CDCl_3_) δ 171.9 (C-1), 161.4 (C-1″), 159.0 (C-2′), 141.1 (C-4′), 122.2 (C-5′), 61.4 (C-3″), 37.9 (C-2), 18.3 (C-3), 14.3 (C-4″), 13.6 (C-4). DIP-MS (ESI; M + Na^+^) *m*/*z* (calculated): 265.06, *m*/*z* (measured): 265.10.

Ethyl 2-Hexanamidothiazol-4-Carboxylate, **16b**

Yield: 85%. Brownish solid; m.p. 129–132 °C. Lit. [30] 135–136 °C *R*_f_ = 0.66 (AcOEt). IR (CH_2_Cl_2_) $\bar{\upsilon }$ = 3256.8, 1723.4, 1680.7, 1547.6, 1211.1 cm^−1^. ^1^H NMR (300 MHz, CDCl_3_) δ: 7.82 (s, 1H, H-5′), 4.35 (q, *J =* 7.13 Hz, 2H, H-3″), 2.44 (t, *J =* 7.59 Hz, 2H, H-2), 1.68 (m, 2H, H-3), 1.36 (t, *J =* 7.13 Hz, 3H, H-4″), 1.28 (m, 4H, H-4,5), 0.86 (m, 3H, H-6). ^13^C NMR (75 MHz, CDCl_3_) δ 171.9 (C-1), 161.4 (C-1″), 158.8 (C-2), 141.19 (C-4′), 122.18 (C-5′), 61.37 (C-3″), 36.05 (C-2), 31.21 (C-4), 24.6 (C-3), 22.3 (C-5), 14.3 (C-4″), 13.8 (C-6). DIP-MS (ESI; M + Na) *m*/*z* (calculated): 293.09, *m*/*z* (measured): 293.16. 

Ethyl 2-Octanamidothiazol-4-Carboxylate, **16c**

Yield: 80%. Brownish solid; m.p. 102–104 °C. Lit. [30] 109–111 °C. *R*_f_ = 0.68 (AcOEt). IR (CH_2_Cl_2_) $\bar{\upsilon }$ = 3245.9, 1725.1, 1689.5, 1544.2, 1214.2 cm^−1^. ^1^H NMR (300 MHz, CDCl_3_) δ: 10.63 (s, 1H, NH), 7.82 (s, 1H, H-5′), 4.35 (q, *J =* 7.11 Hz, 2H, H-3″), 2.44 (t, *J =* 4.54 Hz, 2H, H-2), 1.65 (m, 2H, H-3), 1.36 (t, *J =* 7.11 Hz, 3H, H-4″), 1.24 (m, 8H, H-4,5,6,7), 0.84 (t, *J =* 6.19 Hz, 3H, H-8). ^13^C NMR (75 MHz, CDCl_3_) δ 172.0 (C-1), 161.3 (C-1″), 158.9 (C-2′), 141.1 (C-4′), 122.2 (C-5′), 61.4 (C-3″), 36.1 (C-2), 31.6 (C-6), 29.0 (C-5), 28.9 (C-4), 24.9 (C-3), 22.5 (C-7), 14.31 (C-4″), 14.0 (C-8). DIP-MS (ESI; M + Na) *m*/*z* (calculated): 321.12, *m*/*z* (measured): 321.22.

### 6.4. Procedure for the Synthesis of 4-(Thiazolin-2-yl)–aniline, ***25***

4-aminobenzonitrile (4.23 mmol, 1 eq), cysteamine hydrochloride (6.34 mmol, 1.5 eq), potassium carbonate (21.16 mmol, 5 eq) and 5 mL of a EtOH/H_2_O (1:1) mixture were added to an ACE pressure tube equipped with a magnetic stirring bar. The reaction mixture was subjected to heating in a sand bath at 110 °C for 16 h. At the end of this period, it was transferred to a ball flask and the solvent was evaporated under reduced pressure. The residue was suspended in distilled water (20 mL) and liquid-liquid extractions were made with CH_2_Cl_2_ (3 × 15 mL), the combined organic phases were dried with anhydrous Na_2_SO_4_ and evaporated in a ball flask under reduced pressure. The residue was purified by chromatographic column, using silica gel as the stationary phase and an 8:2 hexane/AcOEt mixture as the mobile phase.

4-(Thiazolin-2-yl) Aniline, **25**

Yield: 82%. Pink solid. ^1^H NMR (300 MHz, CDCl_3_) δ: 7.64 (d, *J =* 8.57 Hz, 2H, H-3), 6.63 (d, *J =* 8.57 Hz, 2H, H-2), 4.38 (t, *J =* 8.2 Hz, 2H, H-4′), 3.96 (s, 2H, NH), 3.34 (t, *J =* 8.2 Hz, 2H, H-5′). ^13^C NMR (75 MHz, CDCl_3_) δ 167.9 (C-2′), 149.3 (C-1), 130.0 (C-3), 123.5 (C-4), 114.2 (C-2), 64.9 (C-4′), 33.5 (C-5′). DIP-MS (ESI; M + H) *m*/*z* (calculated): 179.06, *m*/*z* (measured): 179.06.

### 6.5. Synthesis of N-[4-(Thiazolin-2-yl)-phenyl]-carboxamides, ***17***

4-(Thiazolin-2-yl)-Aniline (1.96 mmol, 1 eq) and 2.5 mL of carboxylic acid (butanoic, hexanoic, octanoic acids) were added to a reaction vial equipped with a magnetic stirring bar. The vial was closed and the reaction mixture warmed (butanoic acid at 150 °C, hexanoic acid at 150 °C, octanoic acid at 180 ° C) for 90 min. At the end of that period, the reaction mixture was transferred to an aqueous solution of 20% potassium carbonate (40 mL) and left to stand under constant stirring for 30 min. The suspension was subjected to vacuum filtration and the solid retained on the filter paper was washed with 40 mL of distilled water in two 20 mL portions, then oven-dried at 40 °C. 

*N*-(4-(Thiazolin-2-yl) Phenyl) Butiramide, **17a**

Yield: 85%. Yellow solid; m.p. 138–140 °C. *R*_f_ = 0.3 (AcOEt). IR (CH_2_Cl_2_) $\bar{\upsilon }$ = 3296.6, 1667.6, 1598.3, 1527.5, 845.5 cm^−1^. ^1^H NMR (300 MHz, CDCl_3_) δ: 7.80 (s, 1H, NH), 7.76 (d, *J =* 8.58 Hz, 2H, H-3′), 7.58 (d, *J =* 8.58 Hz, 2H, H-2′), 4.42 (t, *J =* 8.28 Hz, 2H, H-4″), 3.39 (t, *J =* 8.28 Hz, 2H, H-5″), 2.32 (t, *J =* 7.42 Hz, 2H, H-2), 1.73 (m, 2H, H-3), 0.97 (t, *J =* 7.35 Hz, 3H, H-4). ^13^C NMR (75 MHz, CDCl_3_) δ 171.8 (C-1), 168.0 (C-2″), 140.7 (C-1′), 129.3 (C-3′), 128.7 (C-4′), 119.1 (C-2′), 65.0 (C-4″), 39.6 (C-2), 33.7 (C-5″), 19.0 (C-3), 13.7 (C-4). DIP-MS (ESI; M+H) *m*/*z* (calculated): 249.10, *m*/*z* (measured): 249.15.

*N*-(4-(Thiazolin-2-yl) Phenyl) Hexanamide, **17b**

Yield: 88%. Brownish solid; m.p. 129–131 °C. *R*_f_ = 0.46 (AcOEt). IR (CH_2_Cl_2_) $\bar{\upsilon }$ = 3301.0, 1669.4, 1598.7, 1529.8, 843.9 cm^−1^. ^1^H NMR (300 MHz, CDCl_3_) δ: 7.76 (d, *J =* 8.59 Hz, 2H, H-3′), 7.65 (s, 1H, NH), 7.58 (d, *J =* 8.59 Hz, 2H, H-2′), 4.42 (t, *J =* 8.3 Hz, 2H, H-4″), 3.39 (t, *J =* 8.3 Hz, 2H, H-5″), 2.34 (t, *J =* 7.55 Hz, 2H, H-2), 1.70 (m, 2H, H-3), 1.33 (m, 4H, H-4,5), 0.88 (t, *J =* 6.44 Hz, 3H, H-6). ^13^C NMR (75 MHz, CDCl3) δ 171.7 (C-1), 167.9 (C-2″), 140.6 (C-1′), 129.3 (C-3′), 128.7 (C-4′), 119.0 (C-2′), 65.0 (C-4″), 37.8 (C-2), 33.677 (C-5″), 31.4 (C-4), 25.2 (C-3), 22.4 (C-5), 13.9 (C-6). DIP-MS (ESI; M + Na) *m*/*z* (calculated): 299.11, *m*/*z* (measured): 299.20.

*N*-(4-(Thiazolin-2-yl) Phenyl) Octanamide, **17c**

Yield: 79%. Brownish solid; m.p. 68–70 °C. *R*_f_ = 0.51 (AcOEt). IR (CH_2_Cl_2_) $\bar{\upsilon }$ = 3301.9, 1667.5, 1597.6, 1530.8, 844.1 cm^−1^. ^1^H NMR (300 MHz, CDCl_3_) δ: 7.90 (1H, NH), 7.76 (2H, H-3′), 7.59 (2H, H-2′), 4.42 (2H, H-4″), 3.39 (2H, H-5″), 2.35 (2H, H-2), 1.69 (2H, H-3), 1.26 (8H, H-4,5,6,7), 0.86 (3H, H-8). ^13^C NMR (75 MHz, CDCl3) δ 171.7 (C-1), 167.9 C-2″), 140.7 (C-1′), 129.3 (C-3′), 118.9 (C-4′), 64.9 (C-4″), 33.7 (C-2), 31.6 (C-5″), 29.2, 29.0 28.9, 25.5, 22.6, 14.1 (C-8). DIP-MS (ESI; M + H) *m*/*z* (calculated): 305.16, *m*/*z* (measured): 305.27.

### 6.6. Procedure for the Synthesis of 5-Chloro-benzimidazole-2-thione, ***27***

4-Chloro-phenylenediamine (5.61 mmol, 1 Eq), carbon disulfide (7.23 mmol, 1.3 Eq), potassium hydroxide (16.83 mmol, 3 eq) and 5 mL of an ethanol/water mixture (7:3) were placed in an ACE pressure tube charged with magnetic stirring. The reaction mixture was heated at 80 °C for 5 h, then the solvent was evaporated under reduced pressure and 15 mL of a 5% aqueous HCl solution were added to the residue. The suspension was left with stirring for 15 min and subsequently it was vacuum filtered, the solid obtained was washed with 2 × 20 mL of distilled water, the solid was dried at 40 °C in an oven.

5-chloro-1,3-dihydro-2H-Benzimidazol-2-Thione, **27**

Yield: 88%. Brownish solid. ^1^H NMR (300 MHz, Acetone-*d*_6_) δ: 7.17 (dd, *J =* 1.86, 0.86 Hz, 1H, H-6), 7.15 (d, *J =* 0.86 Hz, 1H, H-4), 7.13 (d, *J =* 1.86 Hz, 1H, H-7). ^13^C NMR (75 MHz, Acetone-*d*_6_) δ 170.4 (C-2), 133.768 (C-4a), 131.7 (C-7a), 127.1 (C-5), 122.1 (C-6), 110.5 (C-7), 109.4 (C-4). DIP-MS (ESI; M − H) *m*/*z* (calculated): 182.97, *m*/*z* (measured): 182.88.

### 6.7. Alkylation of 5-Chloro-benzimidazole-2-thione, ***18***

Benzimidazol-2-thione (1.35 mmol, 1 eq), alkyl halide (1.35 mmol, 1 eq) and 15 mL of ethanol were placed in a 100 mL ball flask equipped with a magnetic stirring bar and the reaction mixture was left to stand for 16 h at 60 °C before evaporating the solvent. The solid was suspended in an aqueous solution of 20% potassium carbonate (30 mL) and vacuum filtered. This solid was washed with 2 × 20 mL of distilled water and oven-dried at 40 °C.

2-[(6-chloro-1H-Benzoimidazol-2-yl) thio)-*N-*(3-Chlorophenyl)] Acetamide, **18a**

Yield: 80%. Purple solid; m.p. 228–231 °C. *R*_f_ = 0.28 (hexane/AcOEt 7:3). IR (CH_2_Cl_2_) $\bar{\upsilon }$ = 1652.2, 1586.4, 1423.7 cm^−1^. ^1^H NMR (300 MHz, DMSO-*d*_6_) δ: 10.77 (s, 1H, NH), 7.77 (s, 1H, H-2′), 7.64 (d, *J =* 1.64 Hz, 1H, H-7″), 7.57 (d, *J =* 8.63 Hz, 1H, H-4″), 7.43 (d, *J =* 8.36 Hz, 1H, H-6′), 7.32 (m, 2H, H-5′,5″), 7.10 (d, *J =* 7.84 Hz, 1H, H-4′), 4.41 (s, 2H, H-2). ^13^C NMR (75 MHz, DMSO-*d*_6_) δ 166.1 (C-1), 152.3 (C-2″), 140.5 (C-1′), 137.2 (C-7a″), 134.9 (C-4a″), 133.5 (C-3′), 131.0 (C-5′), 128.3 (C-6′), 124.1 (C-5″), 123.8 (C-4′), 119.0 (C-2′), 118.0 (C-6′), 115.1 (C-4″), 113.8 (C-7″), 36.9 (C-2). DIP-MS (ESI; M-H) *m*/*z* (calculated): 349.99, *m*/*z* (measured): 349.93.

2-[(6-chloro-1H-Benzimidazol-2-yl) thio)-*N-*(4-chlorobenzyl] Acetamide, **18b**

Yield: 83%. Blue solid; m.p. = 115–118 °C. *R*_f_ = 0.15 (hexane/AcOEt 7:3); IR (CH_2_Cl_2_) $\bar{\upsilon }$ = 3735.7, 3649.3, 3283.2, 1640.0, 1557.9, 1393.9 cm^−1^. ^1^H NMR (300 MHz, DMSO-*d*_6_) δ: 8.87 (t, *J =* 5.95 Hz, 1H, NH), 7.54 (d, *J =* 1.98 Hz, 1H, H-7″), 7.48 (d, *J =* 8.64 Hz, 1H, H-4″), 7.23 (m, 5H, H-3′,4′,5″), 4.25 (d, *J =* 5.95 Hz, 2H, H-1′), 4.11 (s, 2H, H-2). ^13^C NMR (75 MHz, DMSO-*d*_6_) δ 167.5 (C-1), 152.1 (C-2″), 139.4 (C-7a″), 138.4 (C-2′), 136.9 (C-4a″), 131.7 (C-5′), 129.4 (C-3′), 128.5 (C-4′), 127.2 (C-6″), 123.0 (C-5″), 115.2 (C-4″), 114.0 (C-7″), 42.3 (C-1′), 35.5 (C-2). DIP-MS (ESI; M-H) *m*/*z* (calculated): 364.00, *m*/*z* (measured): 363.95.

6-chloro-2- (Nonylthio) -1*H*-Benzimidazole, **18c**

Yield: 70%. Pink solid; m.p. 73–76 °C. *R*_f_ = 0.66 (hexane/AcOEt 7:3). IR (CH_2_Cl_2_) $\bar{\upsilon }$ = 3032.6, 1389.6 cm^−1^. ^1^H NMR (300 MHz, CDCl_3_) δ: 9.891 (s, 1H, NH), 7.51 (d, *J =* 1.97 Hz, 1H, H-7), 7.42 (d, *J =* 8.56 Hz, 1H, H-4), 7.16 (dd, *J =* 8.56, 1.97 Hz, 1H, H-5), 3.28 (m, 2H, H-1′), 1.72 (m, 2H, H-2′), 1.28 (m, 12H, H-3′,4′,5′,6′,7′,8′), 0.85 (t, *J =* 6.83 Hz, 3H, H-9′). ^13^C NMR (75 MHz, CDCl_3_) δ 152.6 (C-2), 139.9 (C-7a), 137.9 (C-4a), 127.9 (C-6), 122.7 (C-5), 114.6 (C-4), 113.9 (C-7), 32.7 (C-1′), 31.8, 29.6 (C-2′), 29.4, 29.2, 29.1 (C-3′), 28.7 (C-4′), 22.7, 14.1 (C-9′). DIP-MS (ESI; M-H) *m*/*z* (calculated): 309.11, *m*/*z* (measured): 309.05.

### 6.8. Procedure for the Synthesis of Compounds, ***19***

In a vial for microwave reaction equipped with a magnetic stirring bar, a mixture was made of 5-chloro-2-aminopyridine (3.11 mmol, 1 eq) and 2.5 mL of carboxylic acid (butanoic, hexanoic, nonanoic acids). The vial was closed, and the reaction mixture heated for 90 min (butanoic acid at 150 °C, hexanoic acid at 150 °C, nonanoic acid at 180 °C). Upon completion of that period, the reaction mixture was transferred to a 20% potassium carbonate solution (40 mL) and left to stir for 30 min before being vacuum filtered. The solid was washed with 40 mL of distilled water in two portions of 20 mL and oven-dried at 40 °C. 

*N*-(5-Chloropyridin-2-yl) Butamide, **19a**

Yield: 91%. White solid; m.p. 102–104 °C. *R*_f_ = 0.64 (hexane/AcOEt 7:3). IR (CH_2_Cl_2_) $\bar{\upsilon }$ = 3300.9, 1697.8, 1582.4 cm^−1^. ^1^H NMR (300 MHz, CDCl_3_) δ: 8.41 (s, 1H, NH), 8.22 (d, *J =* 8.95 Hz, 1H, H-3′), 8.19 (d, *J =* 2.54 Hz, 1H, H-6′), 7.65 (dd, *J =* 8.95, 2.54 Hz, 1H, H-4′), 2.36 (t, *J =* 7.43 Hz, 2H, H-2), 1.74 (m, 2H, H-3), 0.98 (t, *J =* 7.38 Hz, 3H, H-4). DIP-MS (ESI; M + Na) *m*/*z* (calculated): 221.04, *m*/*z* (measured): 221.07.

*N*-(5-Chloropyridin-2-yl) Hexanamide, **19b**

Yield: 87%. White solid; m.p. 103–106 °C. *R*_f_ = 0.73 (hexane/AcOEt 7:3). IR (CH_2_Cl_2_) $\bar{\upsilon }$ = 3275.3, 1690.5, 1582.3 cm-1. NMR 1H (600 MHz, CDCl_3_) δ: 8.37 (s, 1H, NH), 8.23 (d, *J =* 8.9 Hz, 1H, H-3′), 8.21 (d, *J =* 2.5 Hz, 1H, H-6′), 7.66 (dd, *J =* 8.9, 2.5 Hz, 1H, H-4′), 2.49 (t, *J =* 7.58 Hz, 2H, H-2), 1.73 (m, 2H, H-3), 1.35 (m, 4H, H-4,5), 0.90 (t, *J =* 7.13 Hz, 3H, H-6). NMR 13C (125 MHz, CDCl_3_) δ 171.9 (C-1), 149.9 (C-2′), 146.3 (C-6′), 138.0 (C-4′), 126.5 (C-5′), 114.8 (C-3′), 37.7 (C-2), 31.3 (C-4), 25.0 (C-3), 22.4 (C-5), 13.9 (C-6). DIP-MS (ESI; M+Na) *m*/*z* (calculated): 249.07, *m*/*z* (measured): 249.11.

*N-*(5-Chloropyridin-2-yl) Nonamide, **19c**

Yield: 90%. White solid; m.p. 64–66 °C. *R*_f_ = 0.8 (hexane/AcOEt 7:3). IR (CH_2_Cl_2_) $\bar{\upsilon }$ = 3271.7, 1687.1, 1582.7 cm^−1^. ^1^H NMR (600 MHz, CDCl_3_) δ: 8.874 (s, 1H, NH), 8.25 (d, *J =* 8.88 Hz, 1H, H-3′), 8.19 (d, *J =* 2Hz, 1H, H-6′), 7.64 (dd, *J =* 8.88, 2 Hz, 1H, H-4′), 2.38 (t, *J =* 7.59 Hz, 2H, H-2), 1.70 (m, 2H, H-3), 1.28 (m, 10H, H-4,5,6,7,8), 0.85 (t, *J =* 6.88 Hz, 3H, H-9). ^13^C NMR (125 MHz, CDCl_3_) δ 172.1 (C-1), 150.1 (C-2′), 146.1 (C-6′), 138.7 (C-4′), 126.5 (C-5′), 115.0 (C-3′), 37.6 (C-2), 31.8, 29.3, 29.2, 29.1, 25.3 (C-3), 22.6, 14.0 (C-9). DIP-MS (ESI; M+Na) *m*/*z* (calculated): 291.12, *m*/*z* (measured): 291.19.

### 6.9. Procedure for the Synthesis of N-Substituted 2-(4-formylphenoxy) Acetamides, ***35***

In a 50 mL ball flask loaded with magnetic stirring bar, 15 mL of CH_2_Cl_2_, the corresponding amine (3.91 mmol, 1 eq) and bromine acetyl bromide (4.70 mmol, 1.2 eq) were added. The reaction mixture was stirred at rt for 20 min. Subsequently, 40 mL of a 20% aqueous sodium carbonate solution was added and separated by liquid-liquid extraction with CH_2_Cl_2_ (2 × 10 mL). The combined organic phases were dried with anhydrous Na_2_SO_4_ and filtered, then evaporated under vacuum. Once the residue was dry, it was transferred to a 100 mL ball flask equipped with a magnetic stirring bar and reacted with 4-hydroxybenzaldehyde (4.31 mmol, 1.1 eq) and DBU (3.91 mmol, 1 eq) at 70 °C for 6 h, employing acetonitrile (20 mL) as solvent. Upon completion of that period, the solvent was evaporated under reduced pressure, and 30 mL of a 5% HCl solution was added to the residue. Afterwards liquid-liquid extractions were carried out with AcOEt (3 × 10 mL) and the organic phases were dried with anhydrous Na_2_SO_4_ and filtered. The filtrate was evaporated under reduced pressure and the residue was purified by column chromatography, with silica gel as a stationary phase and a hexane/AcOEt mixture 7: 3 as the mobile phase.

N-(3-Chlorophenyl)-2-(4-Formylphenoxy) Acetamide, **35a**

Yield: 65%. White solid. ^1^H NMR (600 MHz, DMSO-*d*_6_) δ: 10.364 (s, 1H, NH), 9.89 (s, 1H, CHO), 7.90 (d, *J =* 8.77 Hz, 2H, H-3″), 7.85 (t, *J =* 1.81 Hz, 1H, H-2′), 7.55 (d, *J =* 8.11 Hz, 1H, H-6′), 7.35 (t, *J =* 8.11 Hz, 1H, H-5′), 7.20 (d, *J =* 8.77 Hz, 2H, H-2″), 7.14 (d, *J =* 8.11 Hz, 1H, H-4′), 4.89 (s, 2H, H-2). ^13^C NMR (150 MHz, DMSO-*d*_6_) δ 191,7 (CHO), 166.8 (C-1), 163.1 (C-1″), 140.2 (C-1′), 133.6 (C-3′), 132.2 (C-3″), 130.9 (C-5′), 130.6 (C-4″), 123.9 (C-4′), 119.7 (C-2′), 118.5 (C-6′), 115.6 (C-2″), 67.5 (C-2). DIP-MS (ESI; M-H) *m*/*z* (calculated): 288.04, *m*/*z* (measured): 288.05.

*N*-(4-Chlorobenzyl)-2-(4-Formylphenoxy) Acetamide, **35b**

Yield: 73%. White solid. ^1^H NMR (600 MHz, DMSO-*d*_6_) δ: 9.98 (s, 1H, CHO), 8.77 (t, *J =* 6 Hz, 1H, NH), 7.89 (d, *J =* 8.7 Hz, 2H, H-3″), 7.37 (d, *J =* 8.4 Hz, 2H, H-4′), 7.29 (d, *J =* 8.4 Hz, 2H, H-3′), 7.16 (d, *J =* 8.7 Hz, 2H, H-2″), 4.71 (s, 2H, H-2), 4.34 (d, *J =* 6 Hz, 2H, H-1′). ^13^C NMR (150 MHz, DMSO-*d*_6_) δ 191.3 (CHO), 167.2 (C-1), 162.5 (C-1″), 138.2 (C-2′), 131.6 (C-3″), 131.3 (C-5′), 130.1 (C-4″), 129.0 (C-3′), 128.1 (C-4′), 115.2 (C-2″), 66.9 (C-2), 41.1 (C-1′). DIP-MS (ESI; M-H) *m*/*z* (calculated): 302.05, *m*/*z* (measured): 302.09.

2-(4-Formylphenoxy)-*N*-propyl Acetamide, **35c**

Yield: 79%. White solid. ^1^H NMR (500 MHz, DMSO-*d*_6_) δ: 9.87 (s, 1H, CHO), 8.15 (s, 1H, NH), 7.87 (d, *J =* 8.7 Hz, 2H, H-3″), 7.13 (d, *J =* 8.7 Hz, 2H, H-2″), 4.60 (s, 2H, H-2), 3.08 (dd, *J =* 13.15, 6.84, 2H, H-1′), 1.44 (m, 2H, H-2′), 0.82 (t, *J =* 7.42 Hz, 3H, H-3′). ^13^C NMR (125 MHz, DMSO-*d*_6_) δ 191.786 (CHO), 167.3 (C-1), 163.1 (C-1″), 132.1 (C-3″), 130.5 (C-4″), 115.6 (C-2″), 67.5 (C-2), 40.6 (C-1′), 22.8 (C-2′), 11.7 (C-3′). DIP-MS (ESI; M + Na) *m*/*z* (calculated): 244.09, *m*/*z* (measured): 244.10.

### 6.10. Synthesis of N-Substituted 2-(4-(imidazolin-2-yl)-phenoxy) Acetamides, ***32***

Once the aldehydes (1.20 mmol, 1 eq) were obtained, they were placed in a 100 mL ball flask containing 50 mL of acetonitrile. Afterwards, they were subjected to a reaction with ethylenediamine (1.20 mmol, 1 eq) with ultrasound for 15 min. Upon completion of this time, NBS (1.45 mmol, 1.2 Eq) was added to the reaction mixture and it was reacted by ultrasound for 10 min. Then the solvent was evaporated under reduced pressure, followed by the addition of 30 mL of an aqueous solution of 20% NaOH. The resulting suspension was stirred for 20 min and vacuum filtered. The solid obtained was washed with 40 mL of distilled water (2 × 20 mL) and oven-dried at 40 °C. 

*N*-(3-Chlorophenyl)-2-(4-(Imidazolin-2-yl) Phenoxy) Acetamide, **32a**

Yield: 88%. Orange solid; m.p. 192–195 °C. *R*_f_ = 0.17 (MeOH/NH_4_OH 20:1). IR (CH_2_Cl_2_) $\bar{\upsilon }$ = 3584.6, 3203, 1655.9, 1532.8 cm–1. ^1^H NMR (500 MHz, DMSO-*d*_6_) δ: 10.47 (s, 1H, NH), 7.84 (t, *J =* 1.99 Hz, 1H, H-2′), 7.77 (d, *J =* 8.7 Hz, 2H, H-3″), 7.54 (m, 1H, H-6′), 7.33 (t, *J =* 8.12 Hz, 1H, H-5′), 7.13 (m, 1H, H-4′), 7.03 (d, *J =* 8.7 Hz, 2H, H-2″), 4.77 (s, 2H, H-2), 3.56 (s, 4H, H-7″). ^13^C NMR (125 MHz, DMSO-*d*_6_) δ 167.2 (C-1), 163.6 (C-5″), 159.7 (C-1″), 140.3 (C-1′), 133.5 (C-3′), 130.9 (C-5′), 129.1 (C-3″), 124.3 (C-4″), 123.8 (C-4′), 119.6 (C-2′), 118.5 (C-6′), 114.7 (C-2″), 67.4 (C-2), 49.9 (C-7″). DIP-MS (ESI; M-H_2_O + MeOH) *m*/*z* (calculated): 344.11, *m*/*z* (measured): 344.20.

*N*-(4-Chlorobenzyl)-2-(4-(Imidazolin-2-yl) Phenoxy) Acetamide, **32b**

Yield: 90%. Yellow solid; m.p. 149–155 °C. *R*_f_ = 0.2 (MeOH/NH_4_OH 20:1). IR (CH_2_Cl_2_) $\bar{\upsilon }$ = 3250.1, 1594.5, 1545.1, 1516.9, 839.1 cm^−1^. ^1^H NMR (500 MHz, DMSO-*d*_6_) δ: 8.74 (t, *J =* 6.14 Hz, 1H, NH), 7.79 (d, *J =* 8.78 Hz, 2H, H-3″), 7.38 (d, *J =* 8.4 Hz, 2H, H-4′), 7.29 (d, *J =* 8.4 Hz, 2H, H-3′), 7.02 (d, *J =* 8.78 Hz, 2H, H-2″), 4.62 (s, 2H, H-2), 4.35 (d, *J =* 6.14 Hz, 2H, H-1′), 3.59 (s, 4H, H-7″). ^13^C NMR (125 MHz, DMSO-*d*_6_) δ 168.1 (C-1), 163.6 (C-5″), 159.6 (C-1″), 138.8 (C-2′), 131. (C-5′), 129.6 (C-3′), 129.1 (C-3″), 128.6 (C-4′), 124.3 (C-4″), 114.8 (C-2″), 67.4 (C-2), 49.8 (C-7″), 41.7 (C-1′). DIP-MS (ESI; M+H) *m*/*z* (calculated): 344.11, *m*/*z* (measured): 344.02.

2-(4-(Imidazolin-2-yl) Phenoxy)-*N*-Propyl Acetamide, **32c**

Yield: 85%. White solid); m.p. 175–178 °C. *R*_f_ = 0.15 (MeOH/NH_4_OH 20:1). IR (CH_2_Cl_2_) $\bar{\upsilon }$ = 3376.1, 3202.8, 1659.4, 1615.4, 1537.3, 1519.5 cm-1. ^1^H NMR (500 MHz, DMSO-*d*_6_) δ: 8.10 (t, *J =* 5.36 Hz, 1H, NH), 7.75 (m, 2H, H-3″), 6.91 (m, 2H, H-2″), 4.50 (s, 2H, H-2), 3.56 (s, 4H, H-7″), 3.08 (m, 2H, H-1′), 1.43 (m, 2H, H-2′), 0.81 (t, *J =* 7.42 Hz, 3H, H-3′). ^13^C NMR (125 MHz, DMSO-*d*_6_) δ 167.7 (C-1), 163.6 (C-5″), 159.6 (C-1″), 129.0 (C-3″), 124.1 (C-4″), 114.7 (C-2″), 67.4 (C-2), 49.7 (C-7″), 40.5 (C-1′), 22.8 (C-2′), 11.7 (C-3′). DIP-MS (ESI; M+H) *m*/*z* (calculated): 262.15, *m*/*z* (measured): 262.04.

### 6.11. Preparation of Culture Media, Test Compounds, and Inoculum

The Luria Bertani (LB) broth was prepared in one liter of distilled water by adding 10 g peptone, 5 g yeast extract, and 5 g NaCl and then sterilized in an autoclave at 15 psi and 121 °C for 15 min. For the LB solid medium, 15 g of bacteriological agar was added and the solution was made up to a liter with distilled water. Thioglycolate medium BBL was prepared according to the fabricant specifications with distilled water and then sterilized in an autoclave at 15 psi and 121 °C for 15 min. *C. violaceum* CV026 was always grown in the presence of 30 μg/mL of kanamycin. 

The amount of each test compound required for a concentration of 100 mM Pleasewas weighed. With the resulting solution, serial 1:10 dilutions were prepared to obtain concentrations 1000 μM, 100 μM and 10 μM.

### 6.12. Preparation of CV026 Suspensions

*C. violaceum* CV026 from a cryovial was streaked onto L-agar plates containing 30 μg/mL kanamycin. Bacteria were incubated at 29 °C for 24 h. The next day an isolated colony was inoculated into 5 mL LB medium with 30 μg/mL kanamycin, followed by incubation at 29 °C and 200 rpm for 15 h.

### 6.13. Evaluation of Compounds as Quorum Sensing Inhibitors in Chromobacterium violaceum CV026

*C. violaceum* CV026 was cultured in 50 mL of thioglycolate medium until reaching an optical density of 0.12 to 600 nm, then 430 μL of an 80 μM C6-AHL solution were added (680 nM final concentration) to the broth. Subsequently 1980 μL of the suspension was transferred to a 5 mL tube and 20 μL of the dilutions of the test compounds were added until reaching the final concentration of 1000 μM, 100 μM, 10 μM. Tubes were incubated at 29 °C and 800 rpm for 16 h. Upon completion of the incubation time, cell density was determined by absorbance at 720 nm by using the thioglycolate medium as the blank. Finally, the absorbance of violacein was measured. 500 μL of the bacterial culture was placed in a 2 mL tube and then 500 μL of acetone were added. The tubes were vortexed and centrifuged at 15,000 rpm for 1 min. The specific production of violacein was calculated by dividing the value of the reading at 577 nm by that at 720 nm. Experiments with additional concentrations were performed in order to determinate the IC_50_ values. Each experiment was performed 6 times/compound, and the results were graphed. Statistical significance was analyzed by Student’s *t*-test. 

### 6.14. Modeling and Optimization of Ligands.

Docking studies of the target compounds were carried out on the CviR protein (PDB code: 3QP6).

Prior to docking, the ligands were drawn with the ACD/ ChemSketch program [51]. A geometric pre-optimization was created in 3D maintaining the stereochemistry. The 3D structures were submitted to a geometric and energetic optimization at the AM1 (Austin Model 1) semiempirical level using the Gaussian 09 program [52]. The output files were saved as (PDB).

### 6.15. Molecular Docking

The docking studies were performed on the AutoDock 4.2 program, which maintains the macromolecule rigid while allowing flexibility in the ligand. Polar hydrogens were added to the ligand, and protein atoms and partial charges were assigned to the receptor (Kollman) and ligand (Gasteiger). Blind docking was achieve using a grid box of 126 Å^3^ with a grid spacing of 0.375 Å^3^ using the Hybrid Genetic Algorithm of Lamarckian with an initial population of 100 randomized individuals and a maximum number of energy evaluations of 10^7^. The results of these docking simulations experiments were examined on AutoDockTools version 1.5.2 and PyMol visualizer to describe the non-bond interaction from the ligand-protein complex.

## 7. Conclusions

Compounds **16a**–**c**, **17a**–**c**, **18a**–**c**, **19a**–**c** and **32a**–**c** were synthesized in moderate to very good global yields, finding MW energy and ultrasound to be extremely useful for the corresponding reactions. The synthesis of the ethyl 2-acylamino thiazole-4-carboxylate **16a**–**c**, was improved using MW in the acylation of the electronically deactivated amino, obtaining higher yields than those reported previously. A methodology was established in the current contribution for the synthesis of *N*-[4-(thiazolin-2-yl) -phenyl]-carboxamides using MW as an energy source and obtaining 3 new compounds. Three novel benzimidazoles **18a**–**c** were synthesized. A new methodology was developed for the synthesis of *N*-(5-chloropyridin-2-yl) amides using MW, obtaining higher yields than those in reported procedures and in shorter times. A new methodology was established for the synthesis of α-phenoxy-imidazolin-2-yl acetamides, involving better yields and shorter times at all reaction stages and achieving 3 new compounds, **32a**–**c**. 

Docking and biological data were in good agreement, which confirms that **16b**–**c**, **17a**–**c**, **18a**–**c**, **19a**–**c**, and **32a**–**c** conduct as bioisosteres of AHL. Such behavior is not attributed to the atom-by-atom or group by group interaction of these compounds with the CviR protein, but rather to the total supramolecular interplay of the compounds with the receptor. Nevertheless, the importance of the π–π interactions emphasizes the fundamental role of the aromatic ring in the inhibitory activity. 

The inhibitory activity of *quorum sensing* shown by the active compounds ranges from low to good, highlighting the activities of benzimidazole **18a** and pyridine **19c**.

## Figures and Tables

**Table 1:**
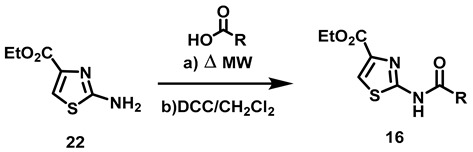
Results from the synthesis of thiazoles **16a**–**c**.

Experiment	R	Thiazole	Procedure	Temperature (°C)	Yield (%)
1	-C_3_H_7_	**16a**	A	150	82
2	-C_3_H_7_	**16a**	B	rt	12
3	-C_5_H_11_	**16b**	A	150	85
4	-C_5_H_11_	**16b**	B	rt	20
5	-C_7_H_15_	**16c**	A	180	80

Conditions: (A) 2-amine thiazole (1 eq), carboxylic acid (2 mL), 90 min. (B) 2-amine thiazole (1 eq), DCC (1 eq), carboxylic acid (1 eq), CH_2_Cl_2_ (20 mL), N_2_ atmosphere, 16 h.

**Table 2:**
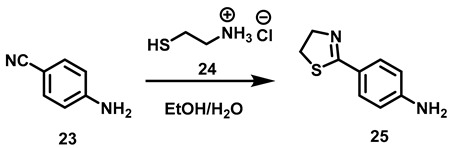
Results obtained to achieve 4-(4′5′-dihydrothiazol-2-yl) aniline **25**.

Experiment	Base (eq)	Time (h)	Temperature (°C)	Yield (%)
1	NaOH (0.2)	2	80	38
2	NaOH (0.2)	16	80	40
3	NaOH (1.5)	16	100	17
4	K_2_CO_3_ (5)	4	110	61
5	K_2_CO_3_ (5)	16	110	82

Conditions: 4-amine benzonitrile, (1 eq), cysteamine hydrochloride (1.5 eq), EtOH/H_2_O 1:1 (5 mL).

**Table 3:**
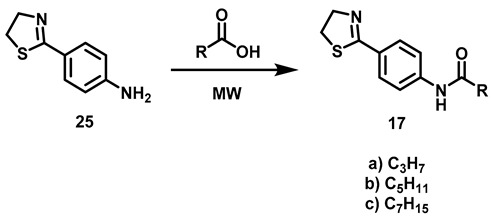
Synthesis of thiazolines **17a**–**c**.

Experiment	R	Compound	Temperature (°C)	Yield (%)
1	-C_3_H_7_	**17a**	150	82
2	-C_5_H_11_	**17b**	150	85
3	-C_7_H_15_	**17c**	180	80

Conditiones: 4-(thiazolin-2-yl)aniline (1 eq), carboxylic acid (2 mL), 90 min.

**Table 4:**
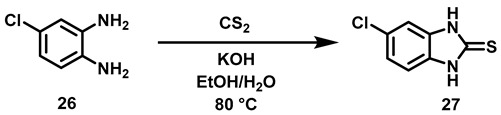
Synthesis of 5-chloro-1,3-dihidro-2*H*-benzo[*d*]imidazole-2-thione **27**.

Assay	Energy	Time (h)	Yield (%)
1	Conventional	3	52
2	MW	3	24
3	Conventional	5	88

Reactions conditions: Phenylenediamine (1 eq), CS_2_ (1.3 eq), KOH (3 eq), EtOH/H_2_O 7:3 (5 mL), 80 °C.

**Table 5:**
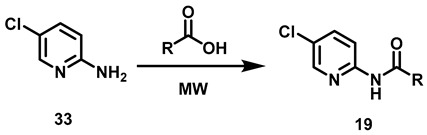
Synthesis of 5-chloropyridin-2-yl)alkylamides **19**.

Experiment	R	Compound	Temperature (°C)	Yield (%)
1	-C_3_H_7_	**19a**	150	91
2	-C_5_H_11_	**19b**	150	87
3	-C_8_H_17_	**19c**	180	90

Conditions: 5-chloropyridine-2-amine (1 eq), carboxylic acid (2 mL), 90 min.

**Table 6:**
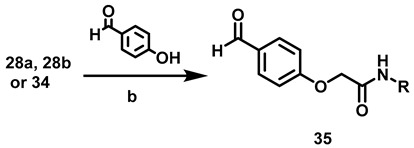
Results from the synthesis of aldehydes **35**.

Experiment	R	Compound	Time (h)	Yield (%)
1	-C_6_H_4_-3-Cl	**35a**	18^20^	63
2	-C_6_H_4_-3-Cl	**35a**	6	65
3	-CH_2_-C_6_H_4_-4-Cl	**35b**	6	73
4	-C_3_H_7_	**35c**	6	79

Conditiones: amide (1 eq), 4-hydroxybenzaldehyde (1.1 eq), DBU (1 eq), acetonitrile (20 mL).

**Table 7:** Affinity energies (∆G) and amino acid residues of the binding site for the ligands evaluated.

Ligand	∆G (kcal/mol)	Amino Acid Residues of the Binding Site
**16a**	−7.13	ILE57, VAL59, TYR80, TRP84 *, LEU85, TYR88, ASP97 *, ILE99, TRP111, PHE115, PHE126, MET135, SER155
**16b**	−7.5	VAL59, MET72, TYR80 *, TRP84, LEU85, TYR88, ASP97 *, ILE99, TRP111, PHE115, PHE126, ALA130, MET135, ILE153, SER155 *
**16c**	−7.19	MET72, TYR80 *, TRP84, LEU85, TYR88, SER89, MET100, ILE153, SER155 *
**17a**	−7.7	VAL75, TYR80 *, LEU85, TYR88 ^▲^, ASP97 *, ILE99, TRP111, SER155 *
**17b**	−8.51	MET72, VAL75, TYR80, TRP84, LEU85, TYR88 ^▲^, ASP97 *, ILE99, TRP111, PHE126, SER155 *
**17c**	−8.77	MET72, VAL75, TYR80, TRP84, LEU85, TYR88, ASP97, ILE99, TRP111, PHE115, PHE126, ALA130, MET135, ILE153, SER155
**18a**	−9.58	ILE57, VAL59, MET72, TYR80 ^▲^, TRP84, LEU85, TYR88, SER89, ILE99, TRP111, PHE126, ALA130, MET135, ILE153, SER155 *
**18b**	−9.51	ILE57, VAL59, MET72, VAL75, TYR80 ^▲^, TRP84, LEU85, TYR88, SER89, ILE99, TRP111, PHE126, ALA130, MET135, SER155 *
**18c**	−8.52	ILE57, VAL59, TYR80, LEU85, TYR88, SER89, ALA94, ILE99, TRP111, PHE126, ALA130, MET135, SER155
**19a**	−7.15	ILE57, VAL59, TYR80, TRP84 *, TYR88, ASP97 *, PHE115, PHE126, ALA130, MET135
**19b**	−7.96	TYR80, TRP84, TYR88 *, ASP97 *, ILE99, TRP111, PHE115, PHE126, MET135, SER155 *
**19c**	−8.09	ILE57, VAL59, MET72, TYR80, TRP84 *, LEU85, TYR88, ASP97, ILE99, TRP111 ^▲^, PHE115, PHE126, ALA130, MET135, SER155 *
**1** (R_1_ = H, R_2_ = C_3_H_7_)	−6.63	ILE57, TYR80, TRP84 *, LEU85, TYR88, ASP97 *, TRP111, PHE126, SER155

Hydrogen bond interaction (*), π–π interaction (^▲^).

## Supplementary Materials

Supplementary materials can be found at https://www.mdpi.com/1422-0067/21/24/9512/s1.

## Abbreviations, Acronyms and Symbols

QS	*quorum sensing*
QSI	*quorum sensing* inhibitor
QSIs	*quorum sensing* inhibitors
AHL	microwave irradiation
MW	microwave irradiation
∆G	affinity energy
UV	ultraviolet
IR	infrared
	Ultrasound
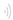
NMR	Nuclear Magnetic Resonance
DIP-MS	Direct Insertion Probe-Mass Spectrometry
ESI	Electrospray Ionization
