# Supplementary material for: N-Heterocycles Scaffolds as Quorum Sensing Inhibitors. Design, Synthesis, Biological and Docking Studies
Source: Int J Mol Sci. 2020 Dec 14;21(24):9512. doi: 10.3390/ijms21249512 (PMC7765044; doi:10.3390/ijms21249512)
Supplement: Supplementary file 1 [file ijms-21-09512-s001.pdf]

Supplementary Material

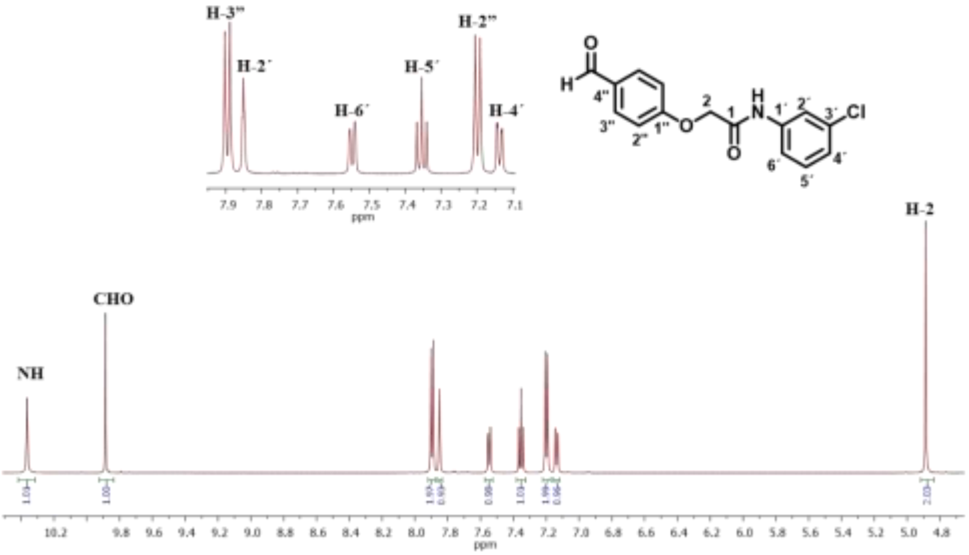

Figure S1. ^{1}H NMR spectrum of compound **35a** (600 MHz, DMSO-d_6).

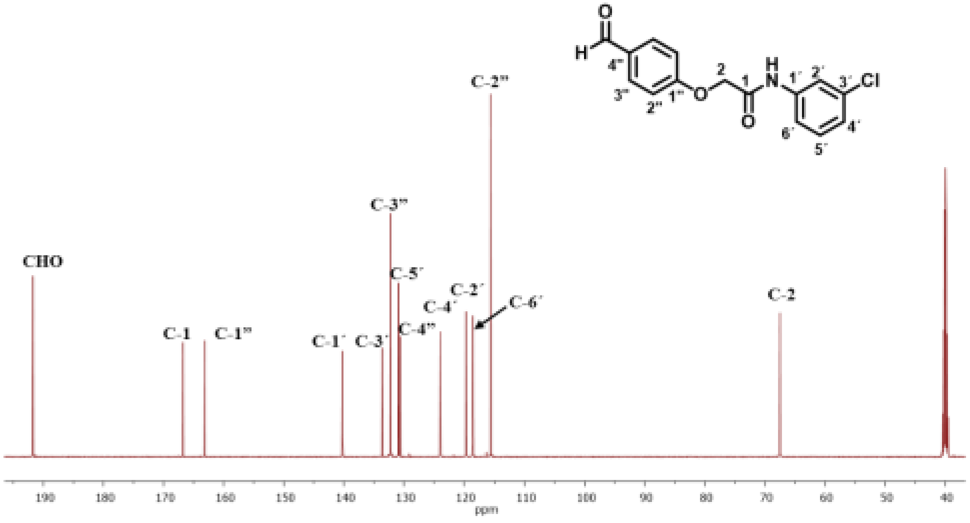

Figure S2. ^{13}C NMR spectrum of compound **35a** (150 MHz, DMSO-d_6).

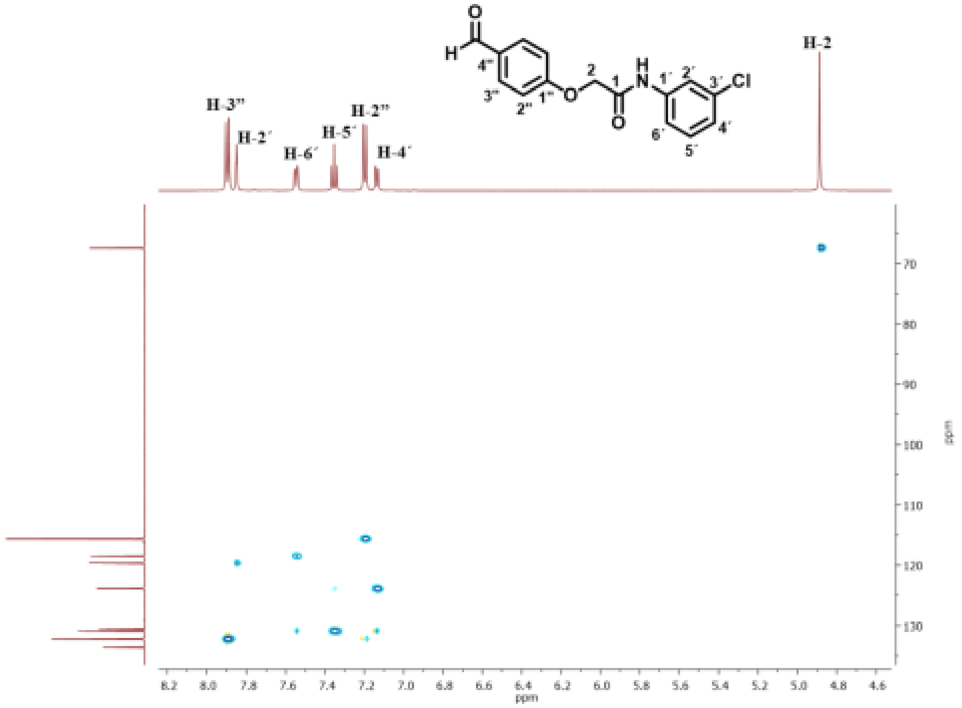

Figure S3. gHSQC spectrum of compound **35a**.

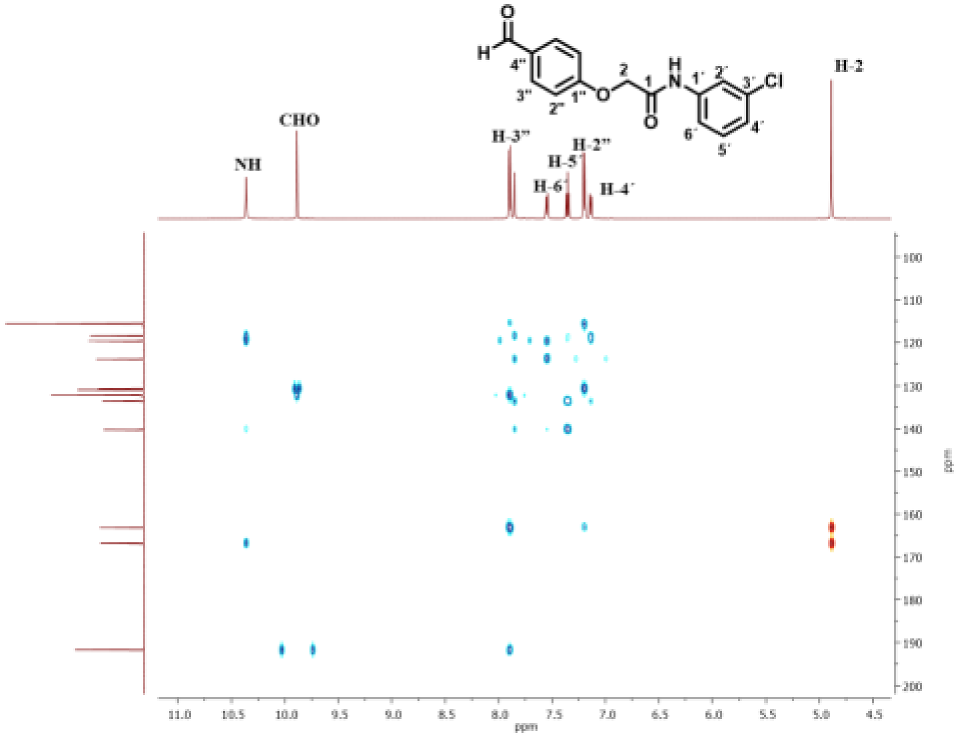

Figure S4. gHMBC spectrum of compound **35a**.

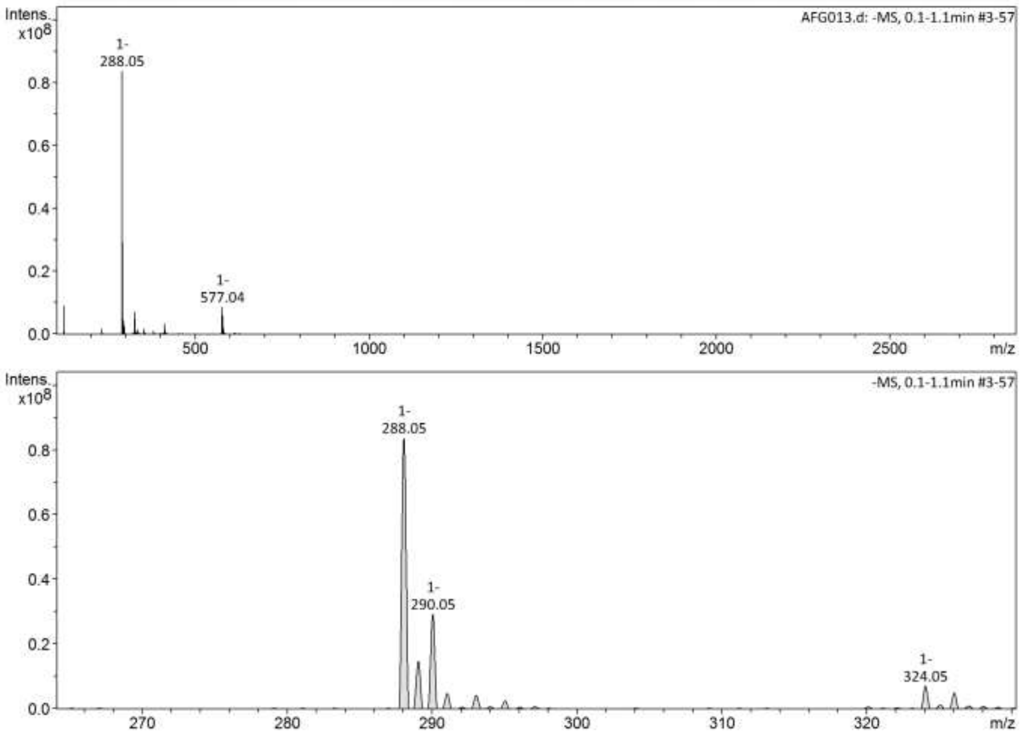

Figure S5. DIP-ESI-MS of compound **35a**.

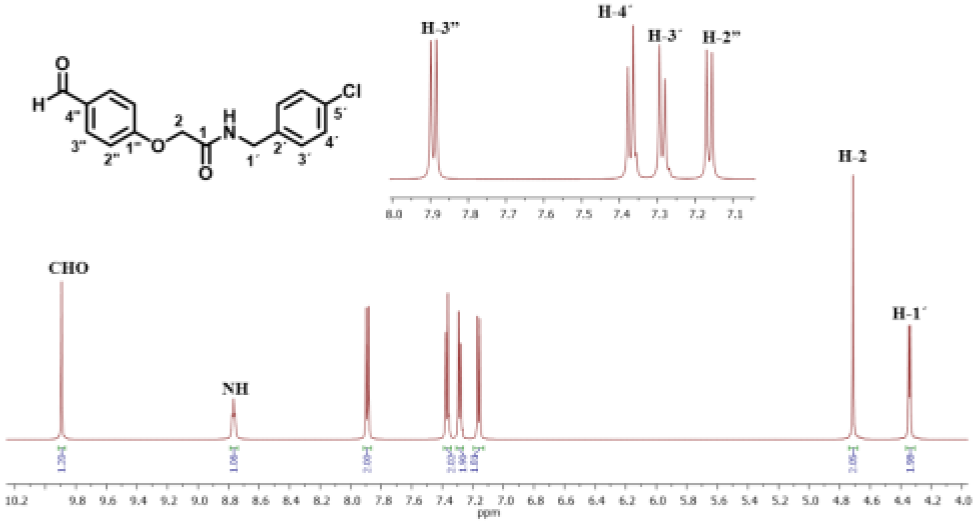

Figure S6. ^{1}H NMR spectrum of compound **35b** (600 MHz, DMSO-d_6).

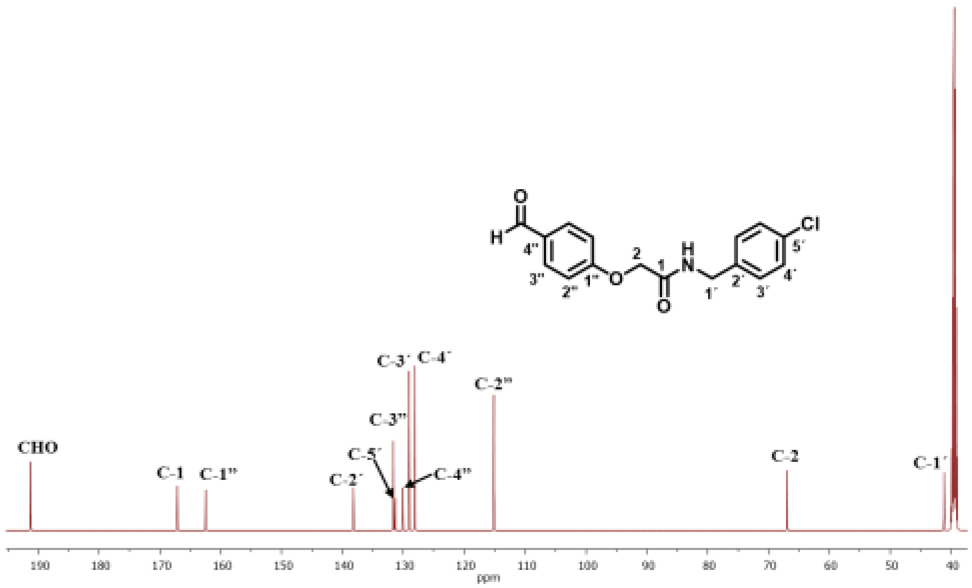

Figure S7. ^{13}C NMR spectrum of compound **35b** (600 MHz, DMSO-d$_6$).

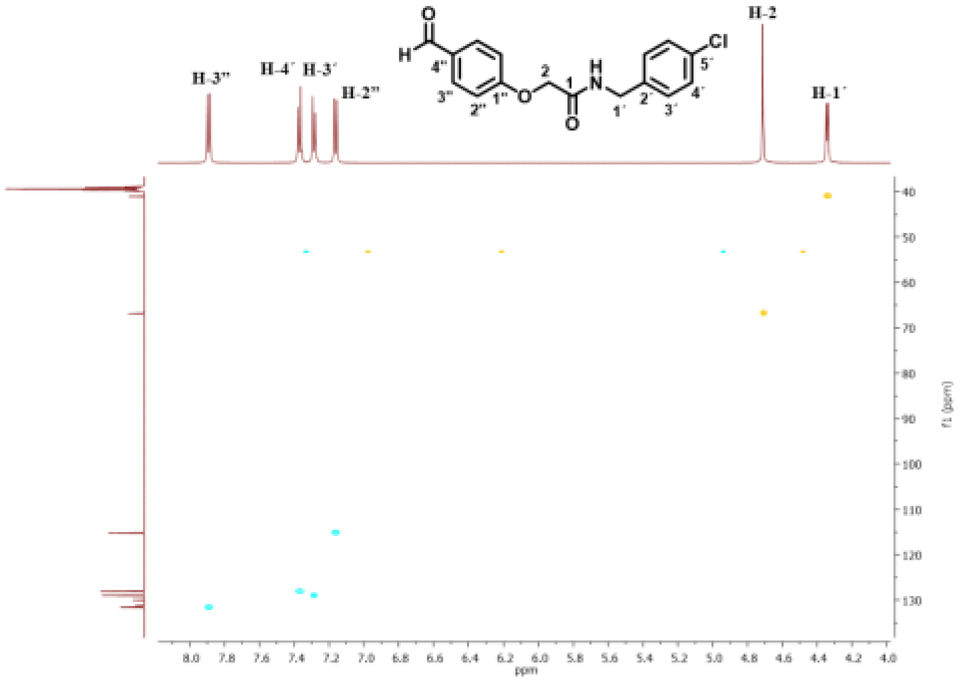

Figure S8. gHSQC spectrum of compound **35b**.

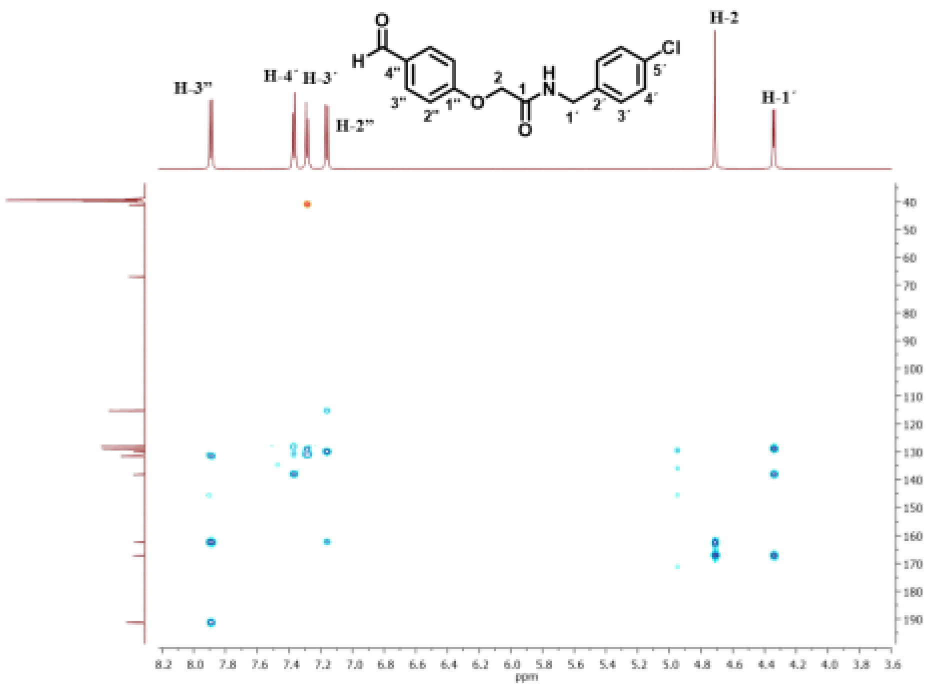

Figure S9. gHMBC spectrum of compound **35b**.

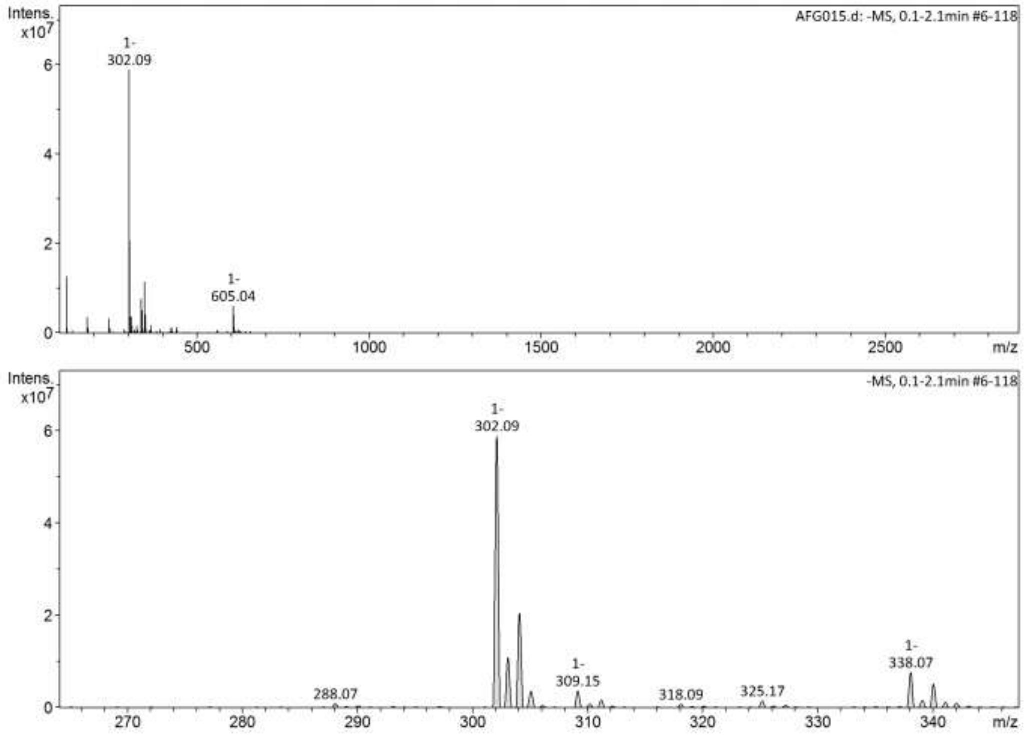

Figure S10. DIP-ESI-MS of compound **35b**.

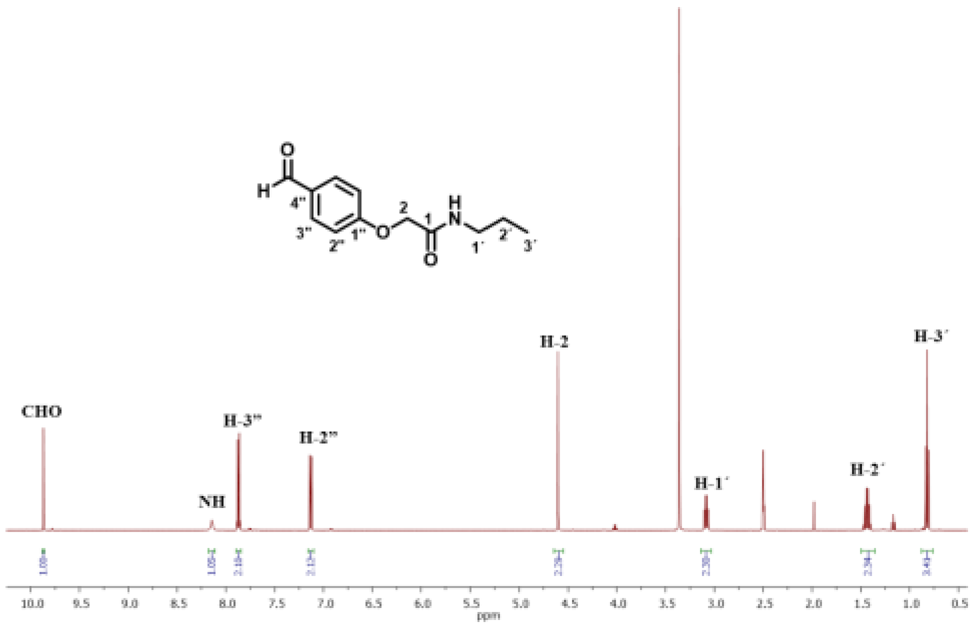

Figure S11. ^{1}H NMR spectrum of compound **35c** (500 MHz, DMSO-d_6).

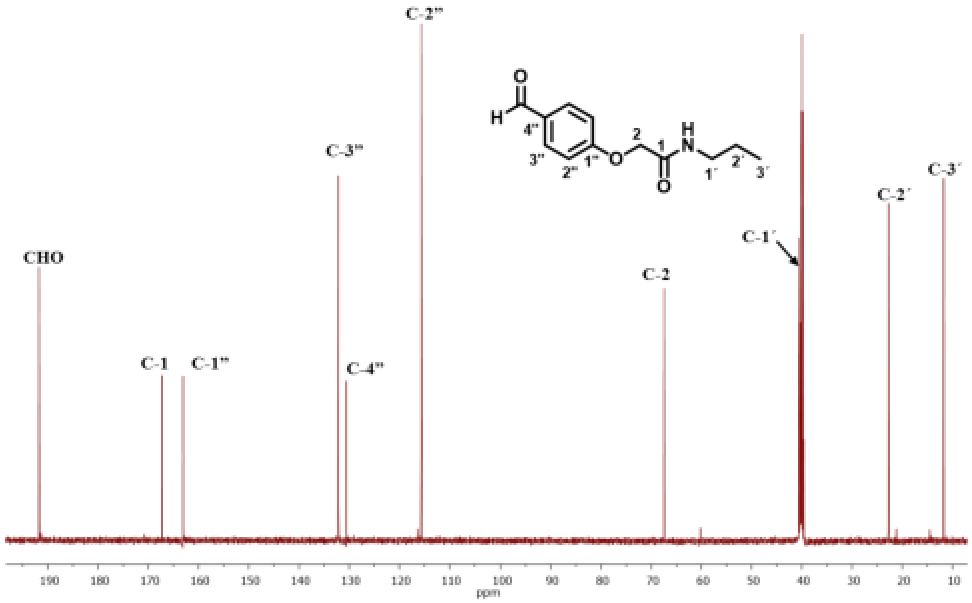

Figure S12. ^{13}C NMR spectrum of compound **35c** (125 MHz, DMSO-d_6).

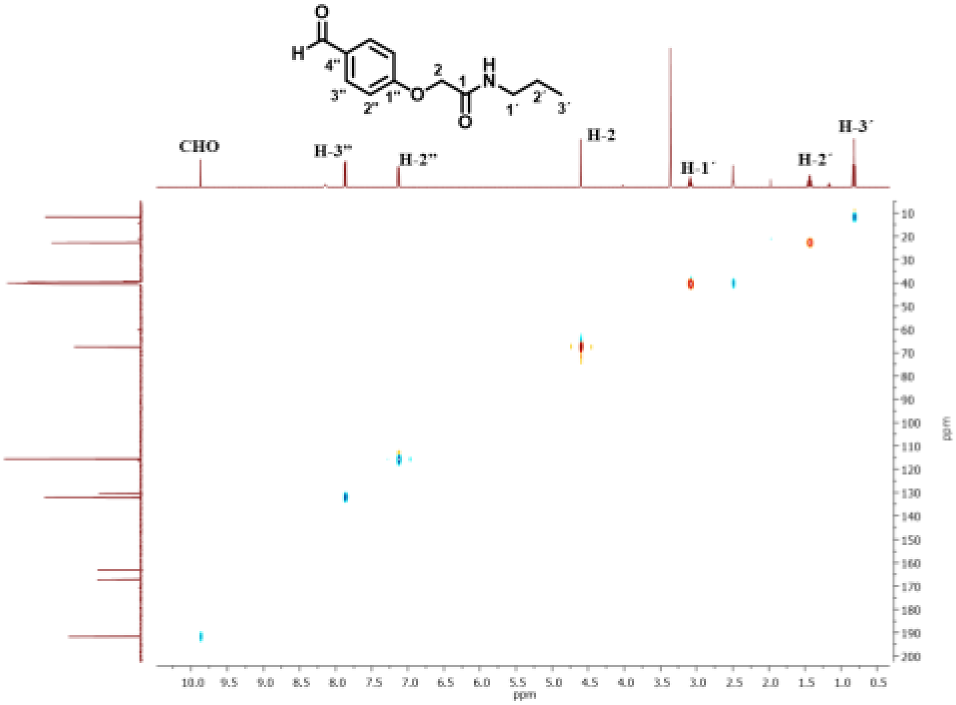

Figure S13. gHSQC spectrum of compound **35c**.

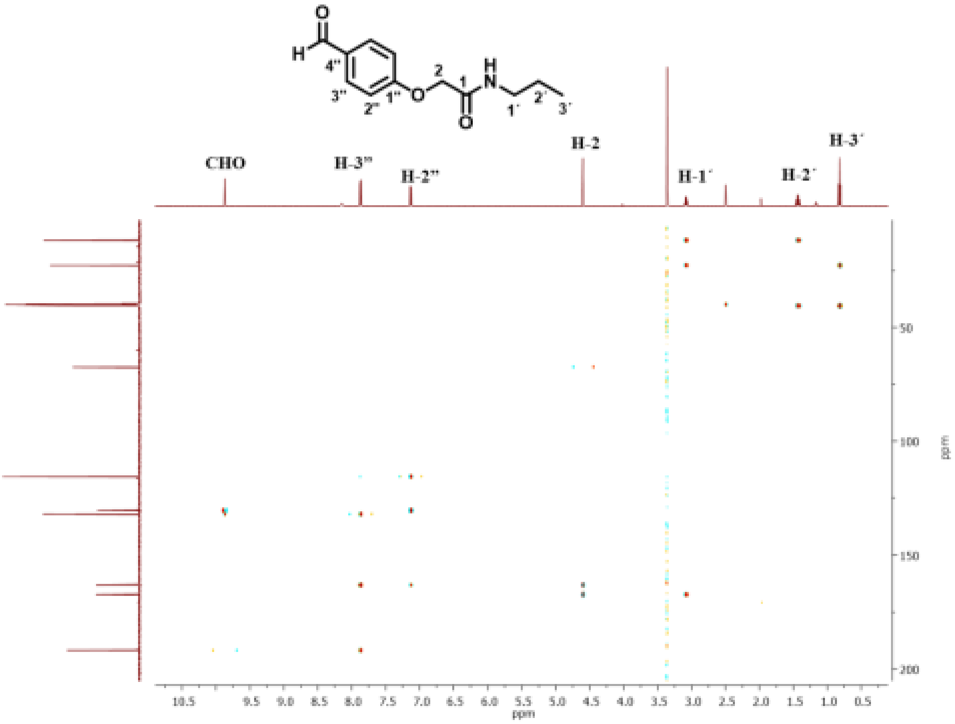

Figure S14. gHMBC spectrum of compound **35c**.

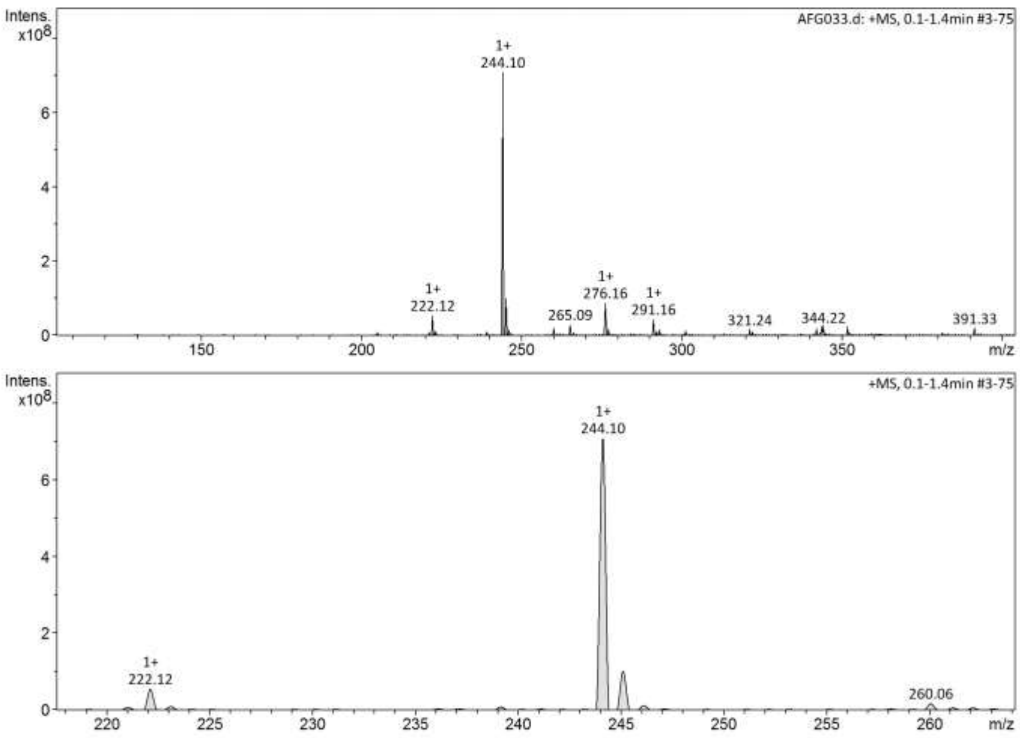

Figure S15. DIP-ESI-MS of compound **35c**.

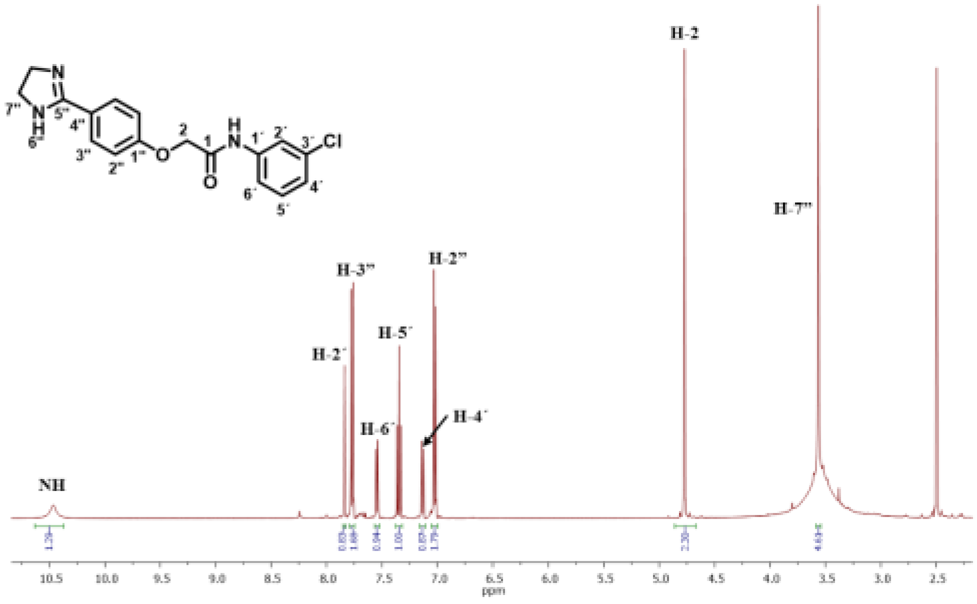

Figure S16. ^{1}H NMR spectrum of compound **32a** (500 MHz, DMSO-d$_6$).

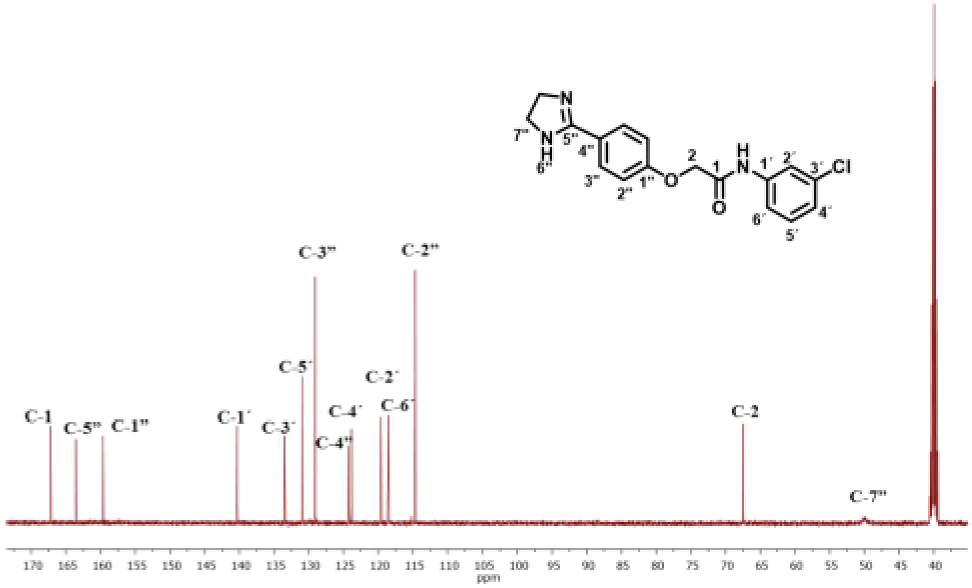

Figure S17. ^{13}C NMR spectrum of compound 3**2a** (125 MHz, DMSO-d$_6$).

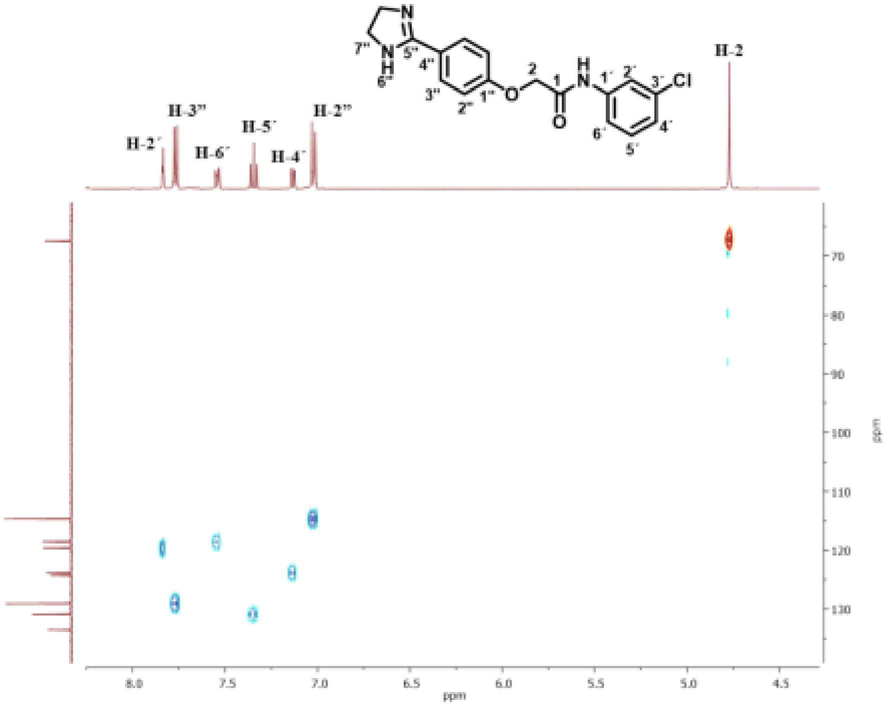

Figure S18. gHSQC spectrum of compound **32a**.

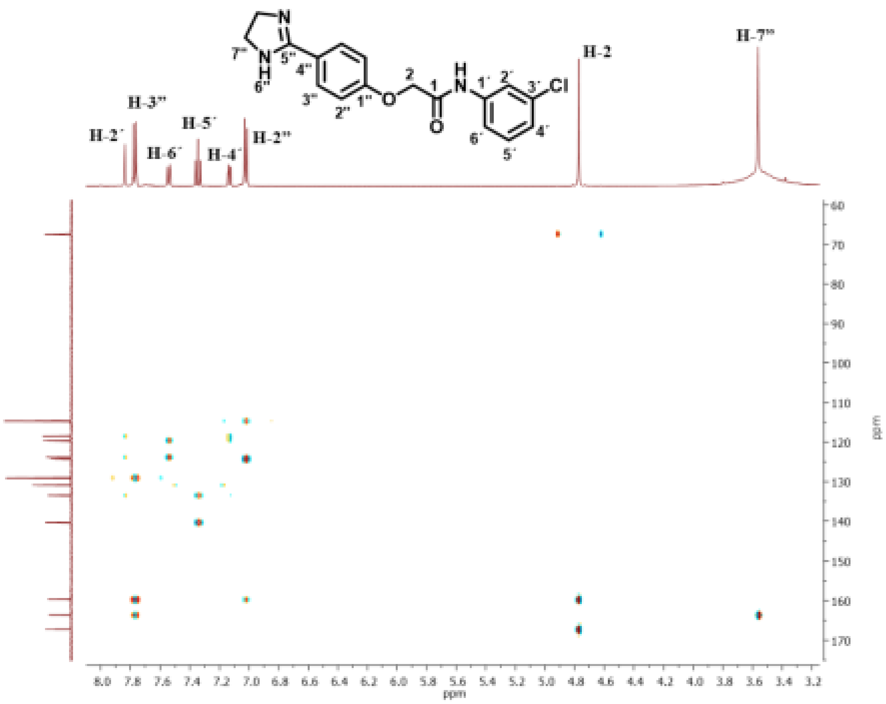

Figure S19. gHMBC spectrum of compound **32a**.

Central de Instrumentación de Espectroscopía ENCB-IPN

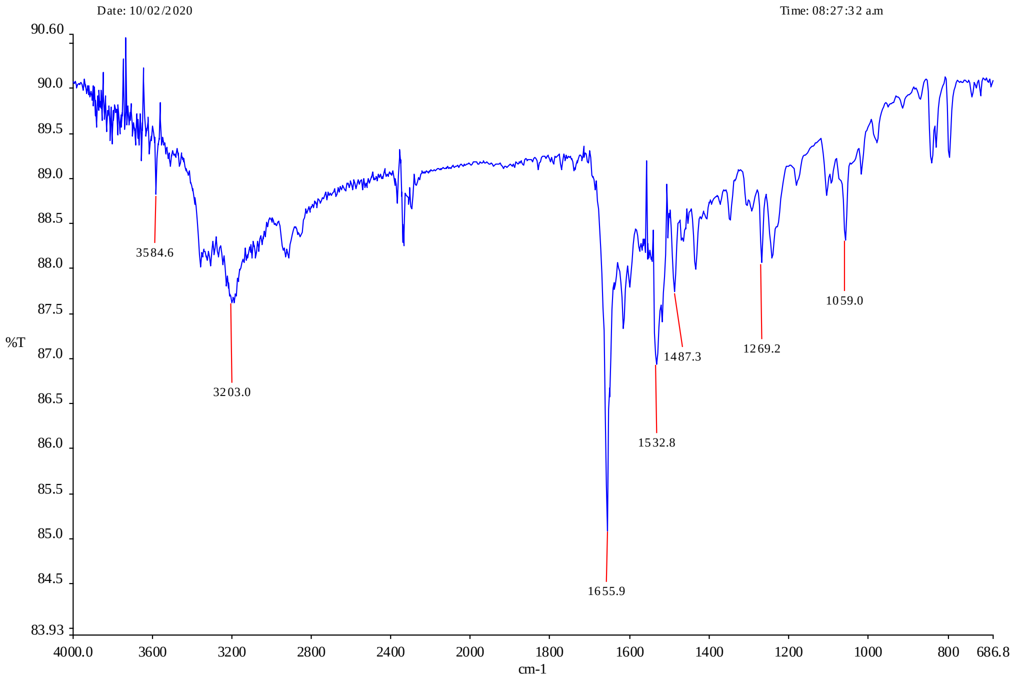

Figure S20. FT-IR spectrum of compound **32a**.

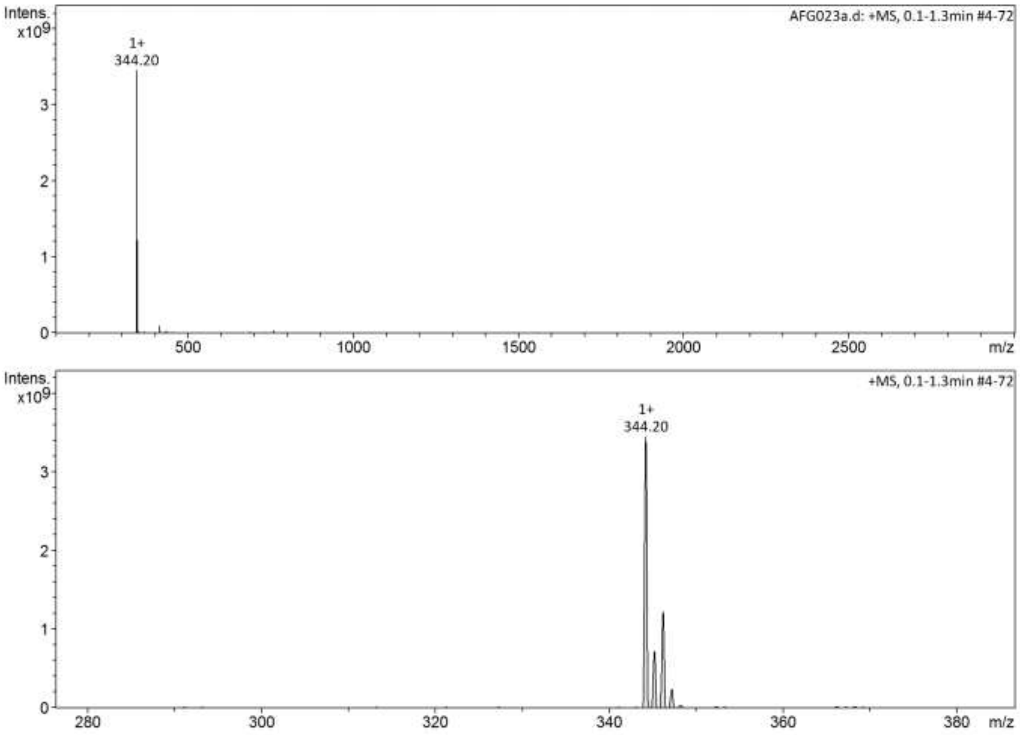

Figure S21. DIP-ESI-MS of compound **32a**.

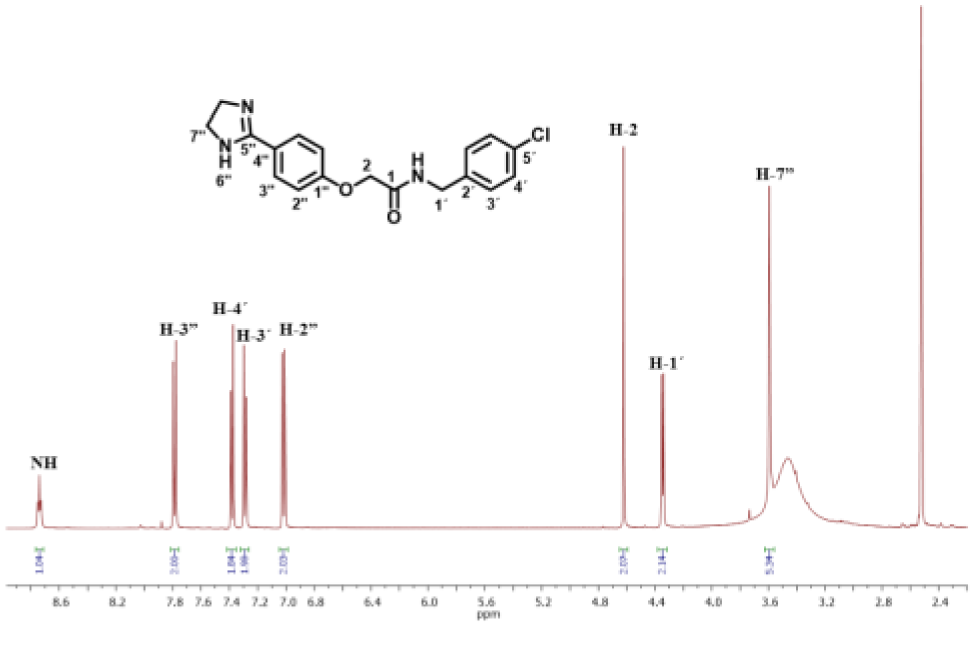

Figure S22. ^{1}H NMR spectrum of compound **32b** (500 MHz, DMSO-d_6).

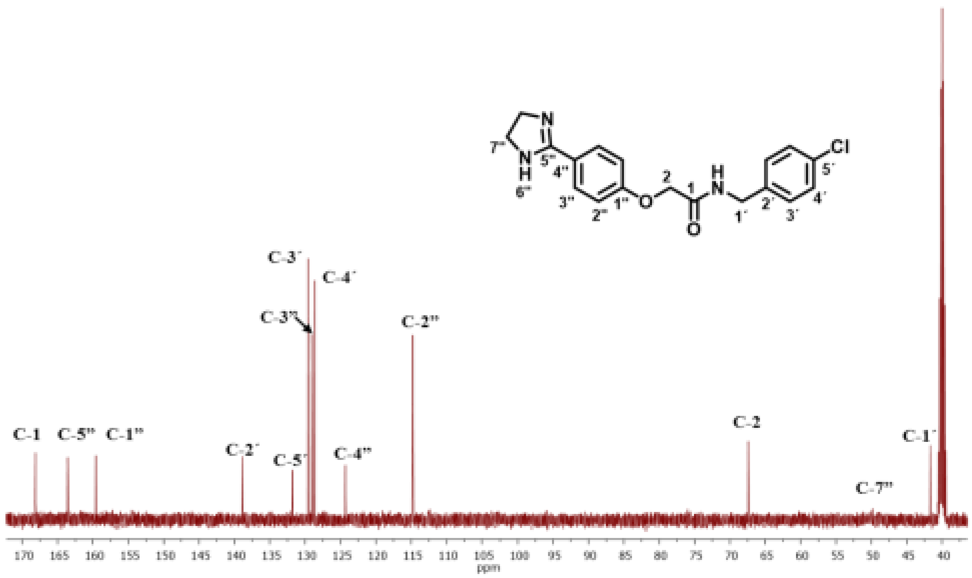

Figure S23. ^{13}C NMR spectrum of compound **32b** (125 MHz, DMSO-d_6).

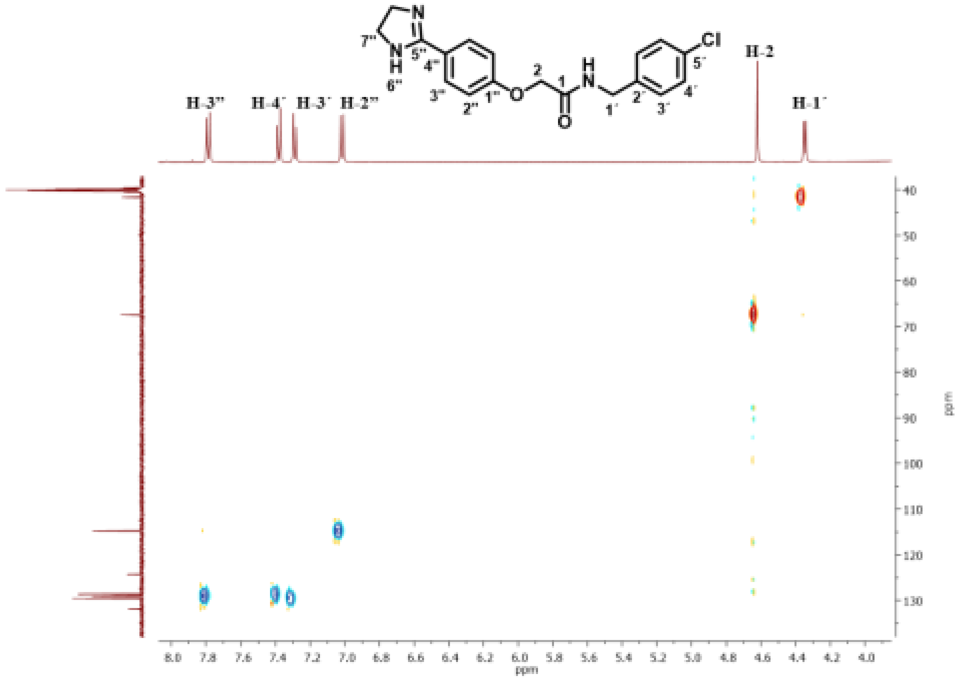

Figure S24. gHSQC spectrum of compound **32b**.

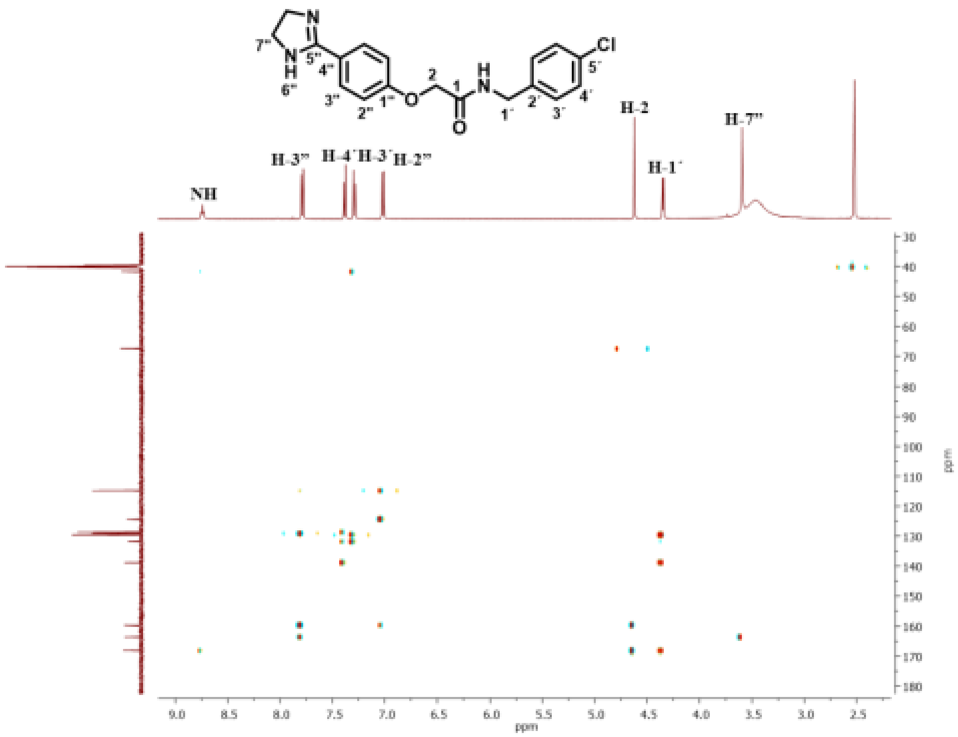

Figure S25. gHMBC spectrum of compound **32b**.

Central de Instrumentación de Espectroscopía ENCB-IPN

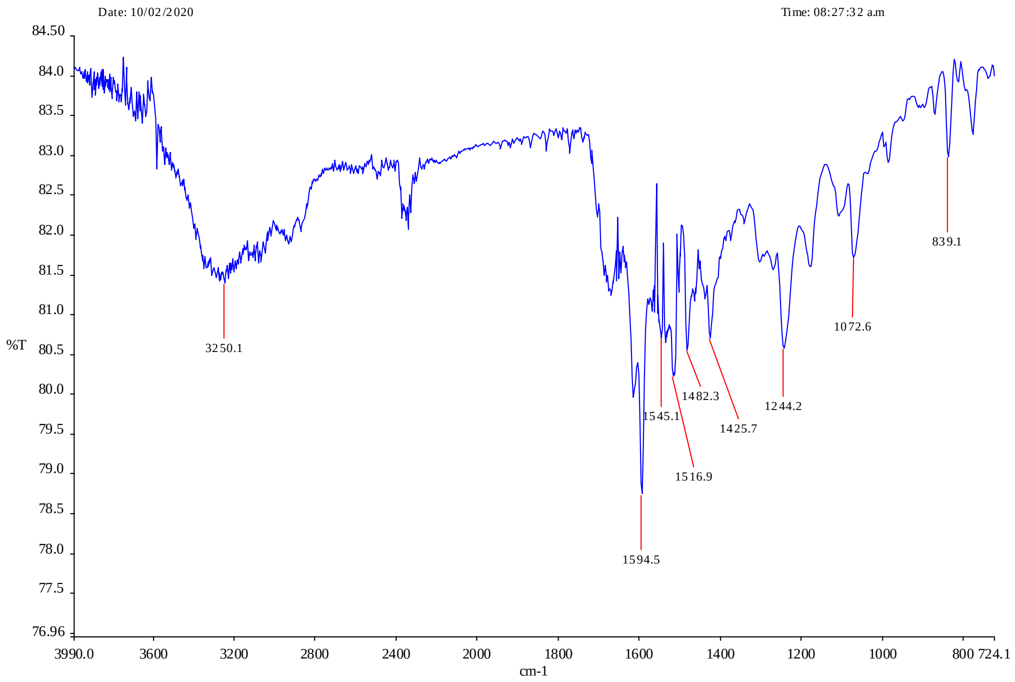

Figure S26. FT-IR spectrum of compound **32b**.

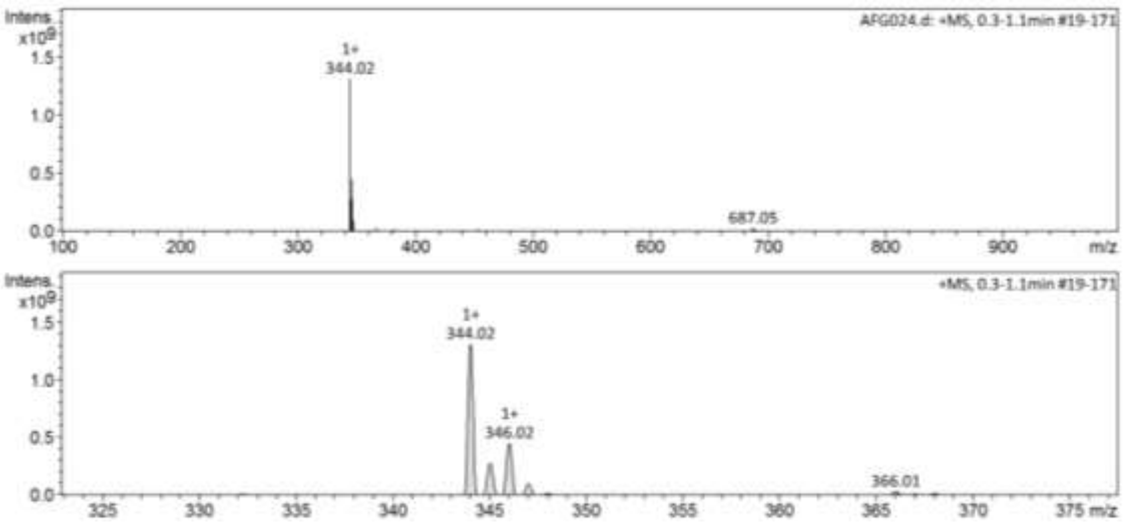

Figure S27. DIP-ESI-MS of compound **32b**.

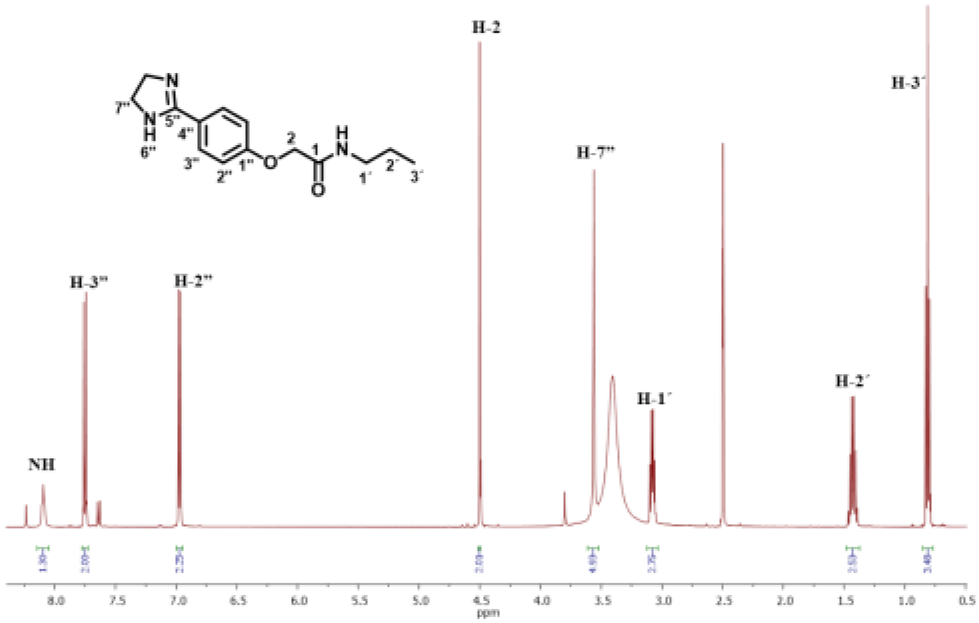

Figure S28. ^{1}H NMR spectrum of compound **32c** (500 MHz, DMSO-d_6).

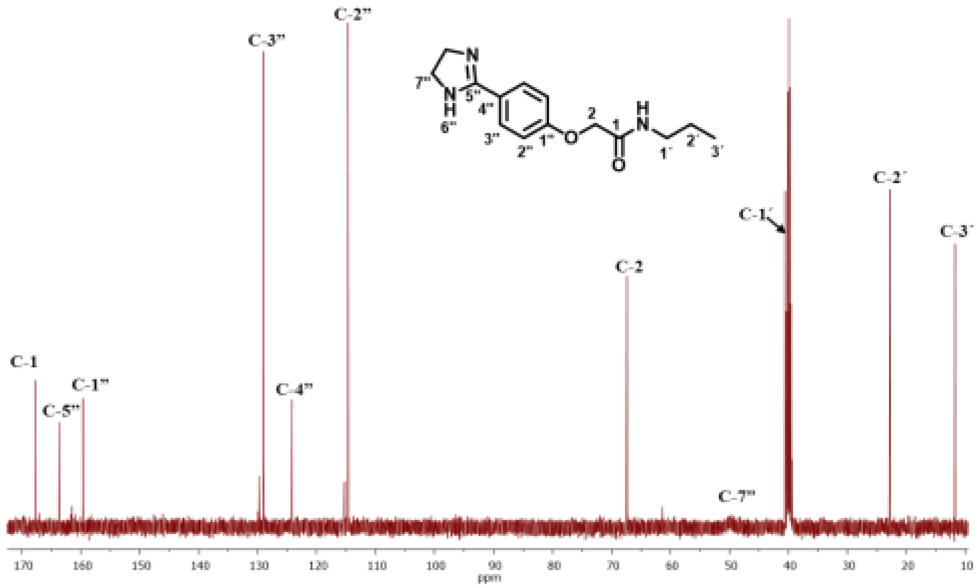

Figure S29. ^{13}C NMR spectrum of compound **32c** (125 MHz, DMSO-d_6).

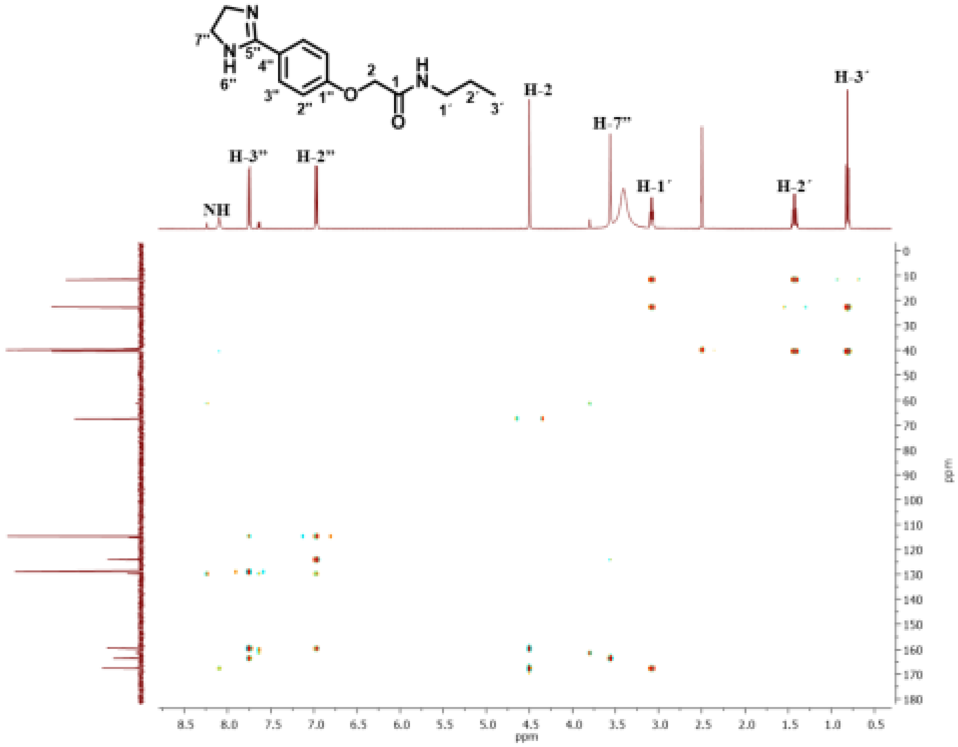

Figure S30. gHMBC spectrum of compound **32c**.

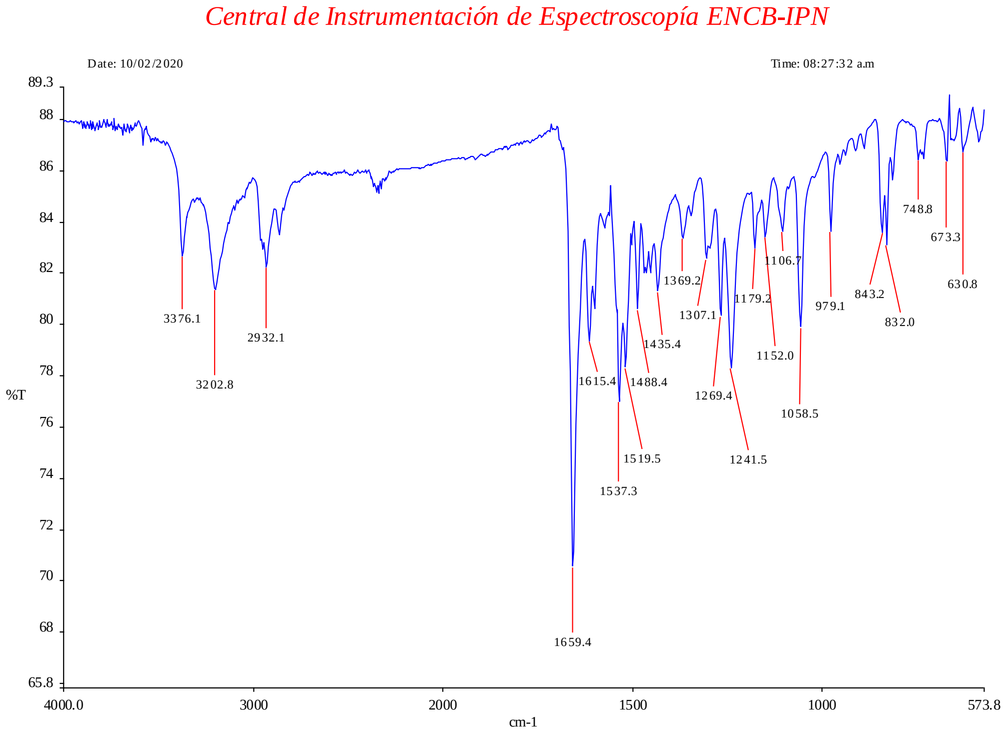

Figure S31. FT-IR spectrum of compound **32c**.

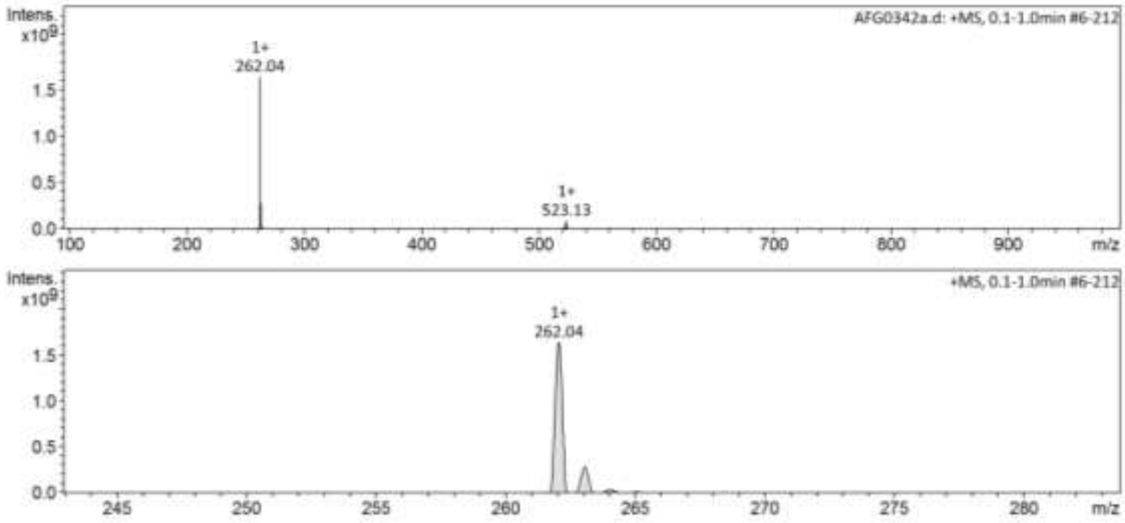

Figure S32. DIP-ESI-MS of compound **32c**.

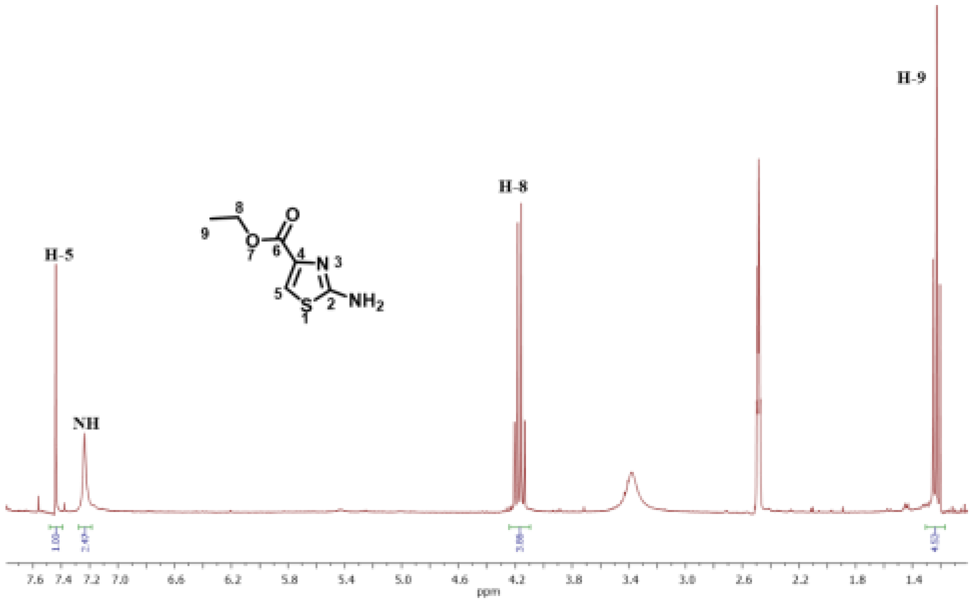

Figure S33. ^{1}H NMR spectrum of compound **22** (300 MHz, DMSO-d_6).

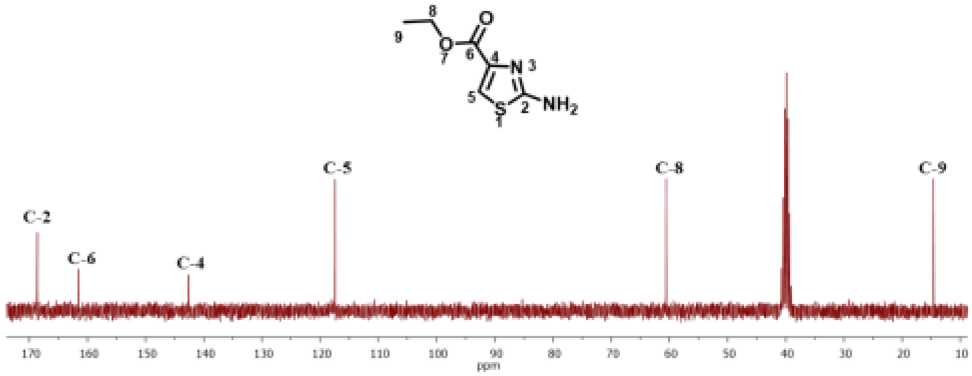

Figure S34. ^{13}C NMR spectrum of compound **22** (75 MHz, DMSO-d_6).

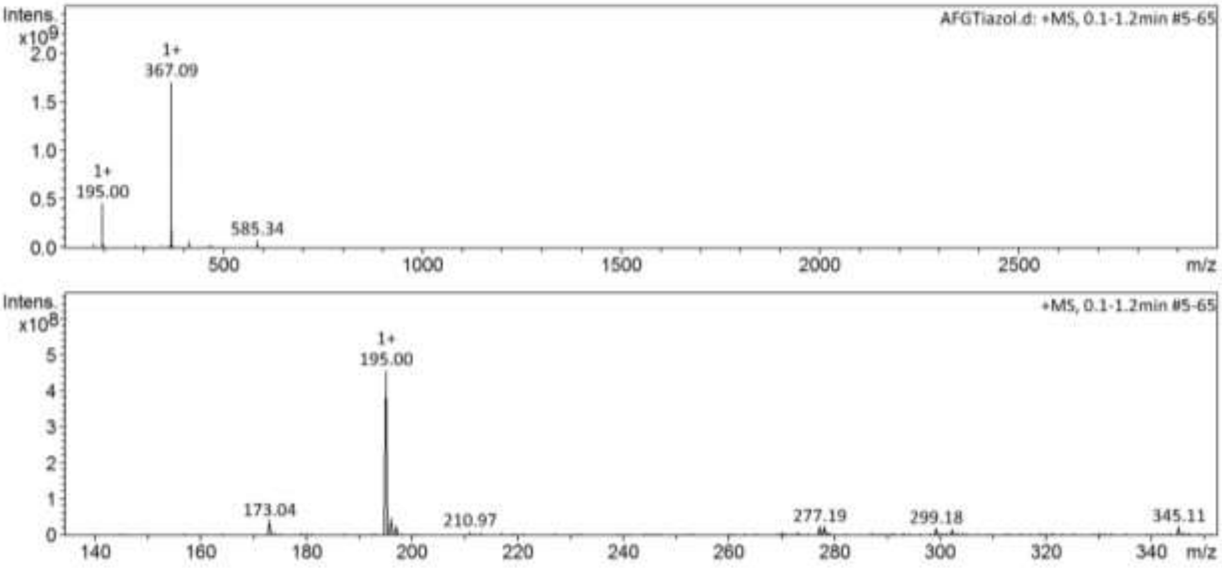

Figure S35. DIP-ESI-MS of compound **22**.

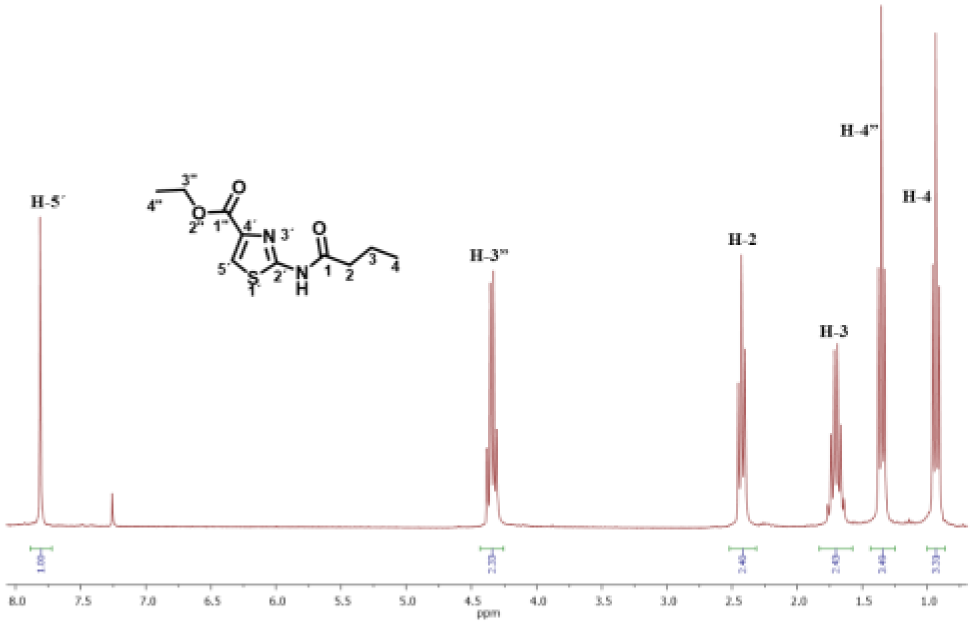

Figure S36. ^{1}H NMR spectrum of compound **16a** (300 MHz, $CDCl_3$).

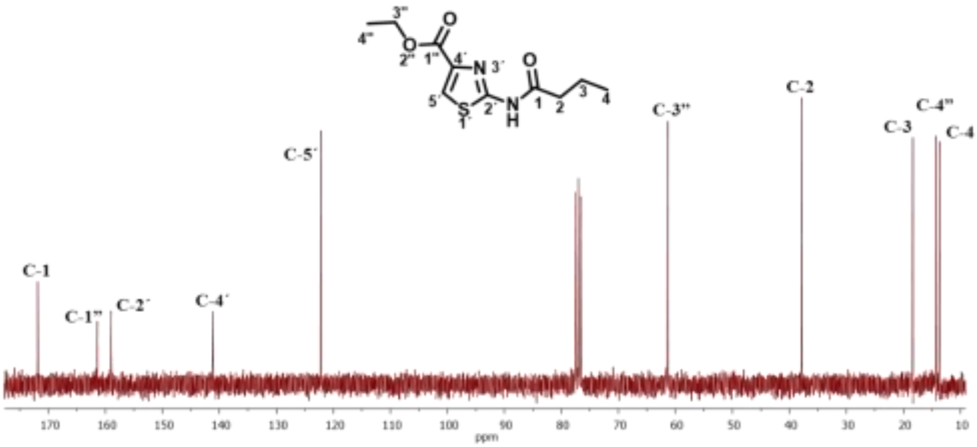

Figure S37. ^{13}C NMR spectrum of compound **16a** (75 MHz, $CDCl_3$).

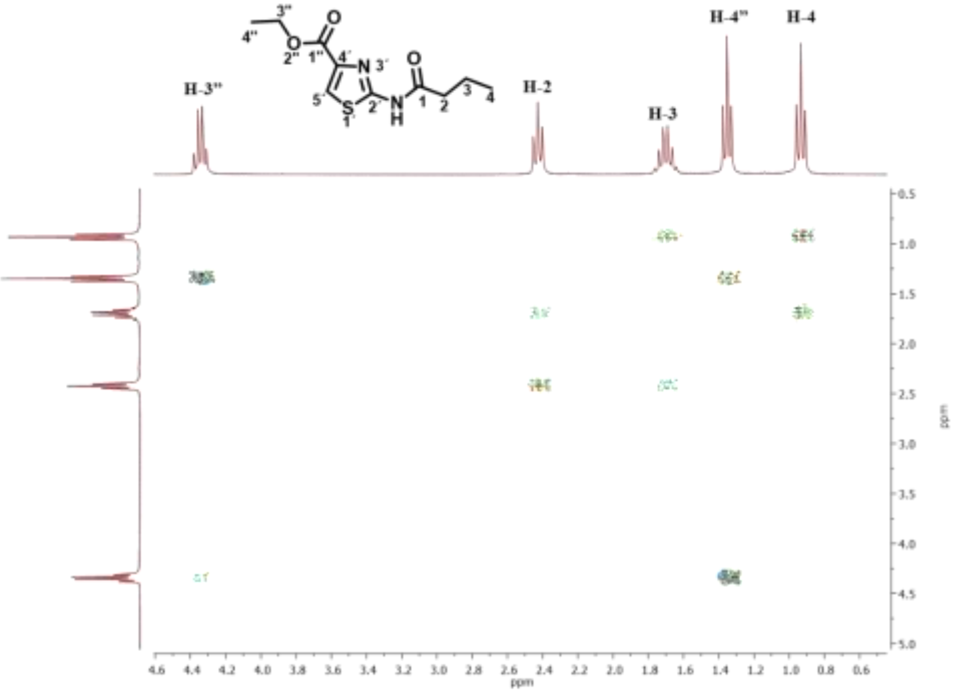

Figure S38. gCOSY spectrum of compound **16a**.

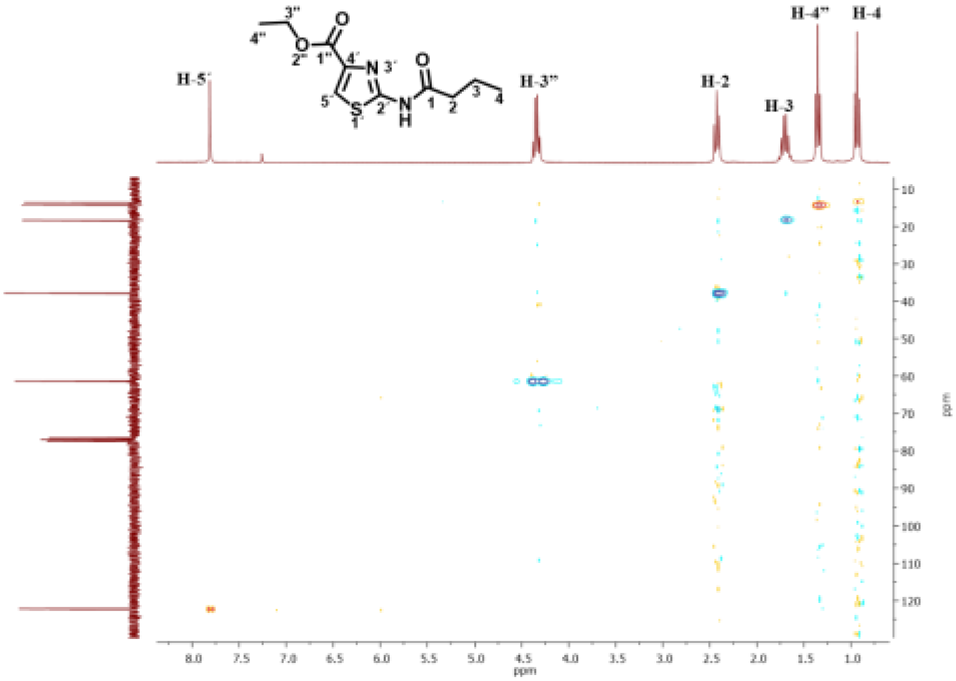

Figure S39. gHSQC spectrum of compound **16a**.

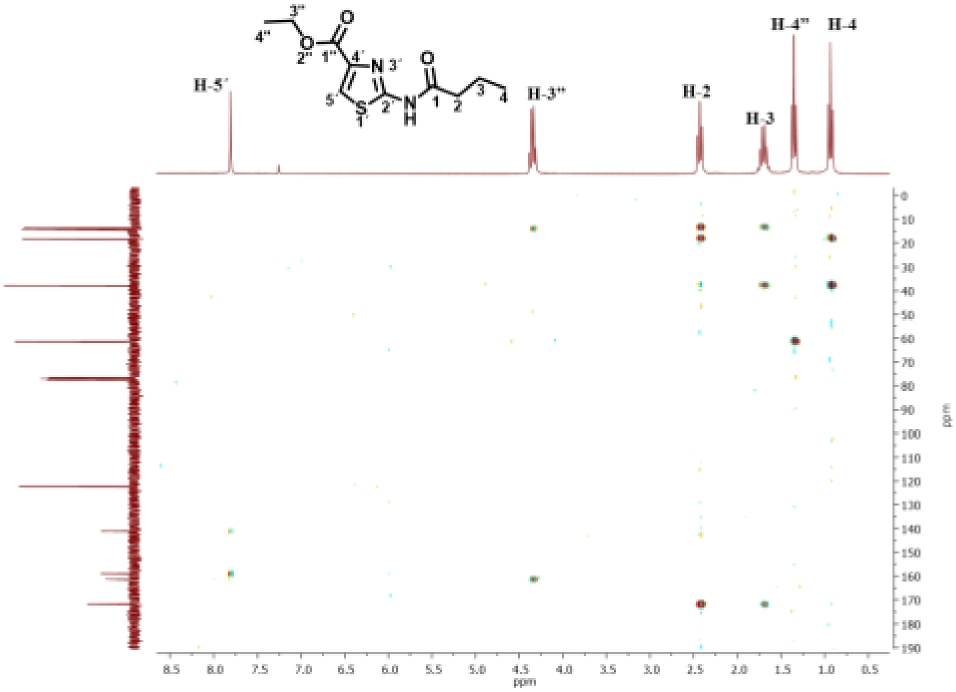

Figure S40. gHMBC spectrum of compound **16a**.

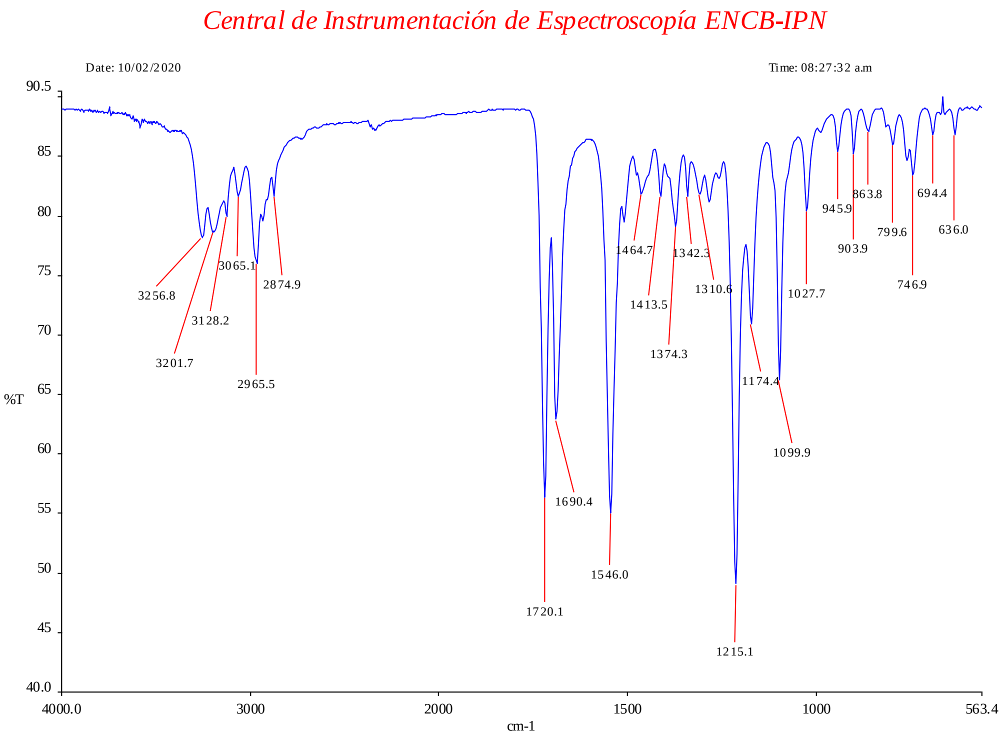

Figure S41. FT-IR spectrum of compound **16a**.

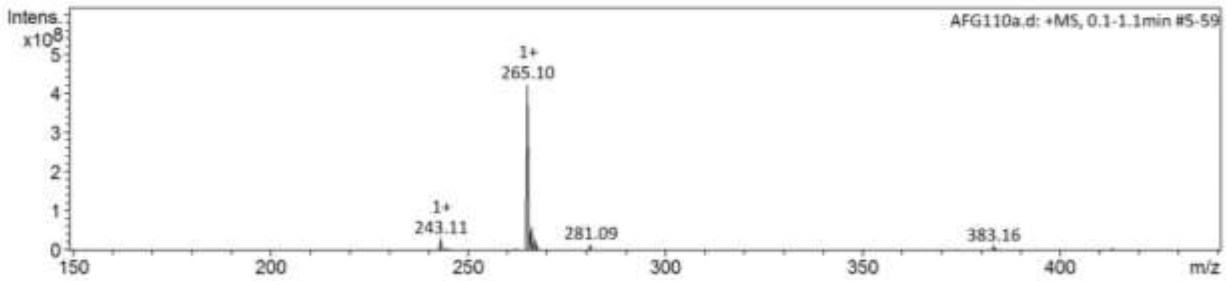

Figure S42. DIP-ESI-MS of compound **16a**.

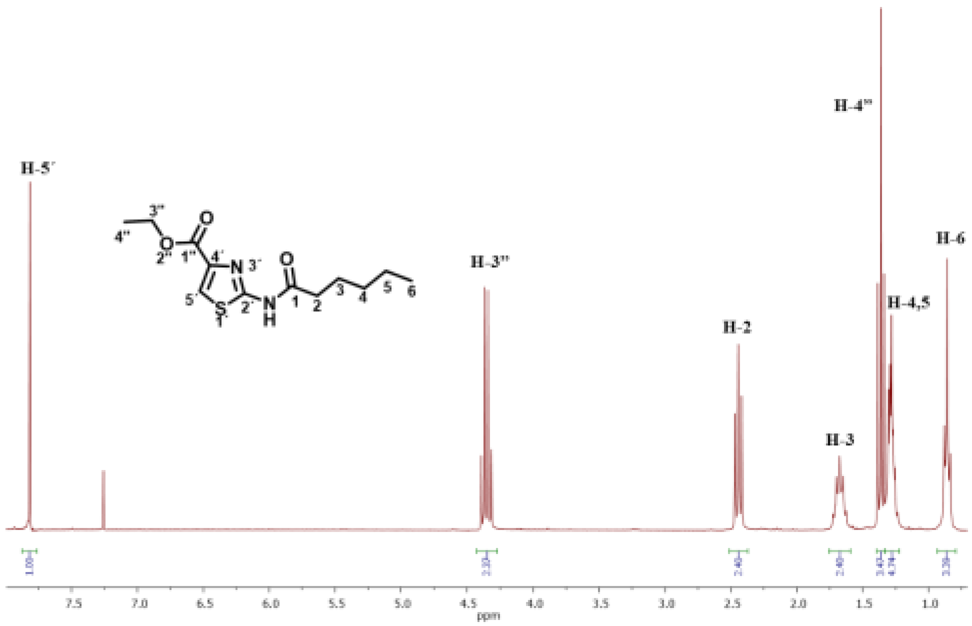

Figure S43. ^{1}H NMR spectrum of compound **16b** (300 MHz, $CDCl_3$).

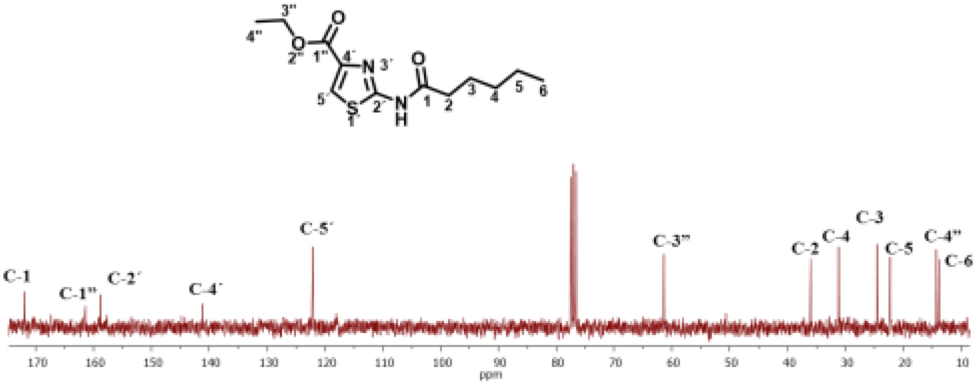

Figure S44. ^{13}C NMR spectrum of compound **16b** (75 MHz, $CDCl_3$).

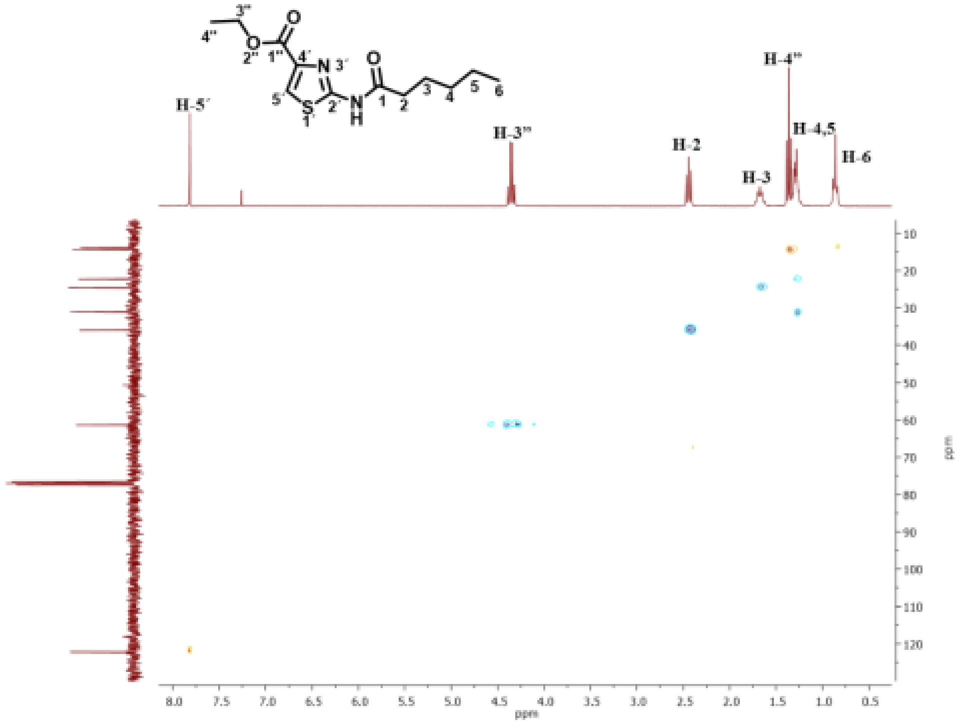

Figure S45. gHSQC spectrum of compound **16b**.

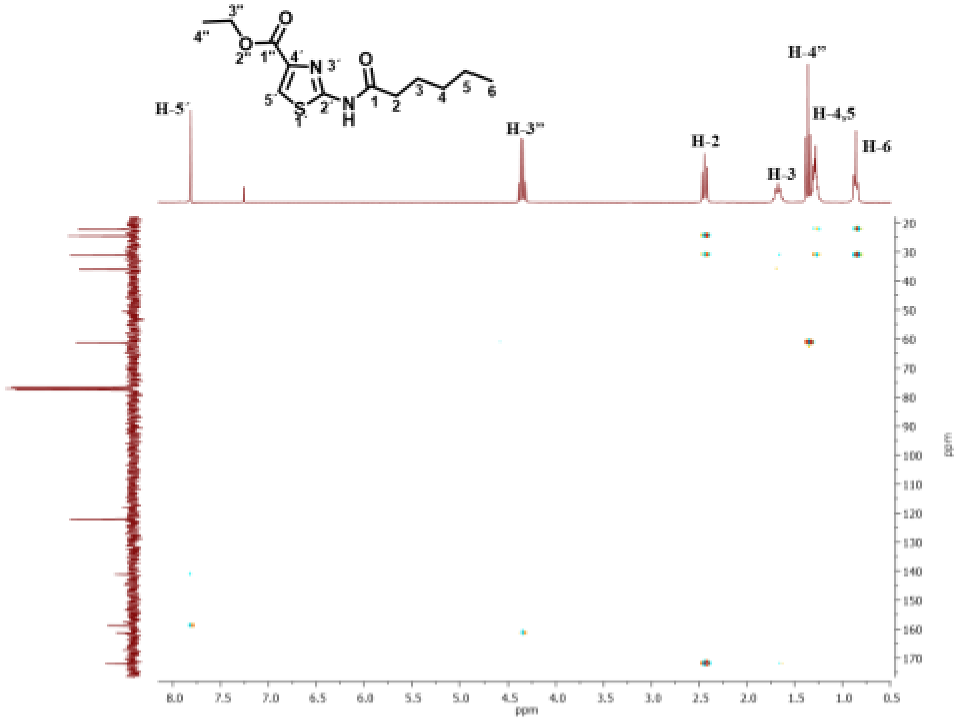

Figure S46. gHMBC spectrum of compound **16b**.

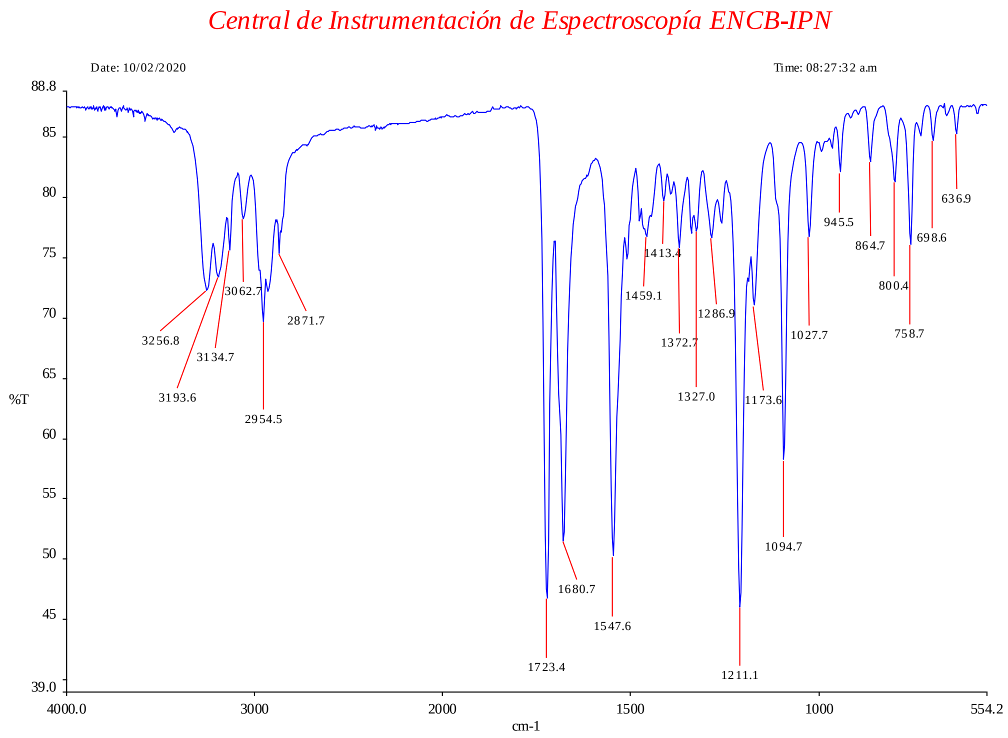

Figure S47. FT-IR spectrum of compound **16b**.

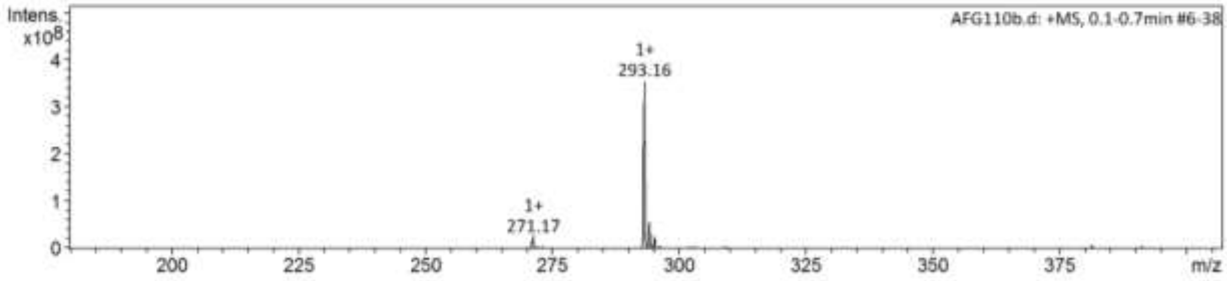

Figure S48. DIP-ESI-MS of compound **16b**.

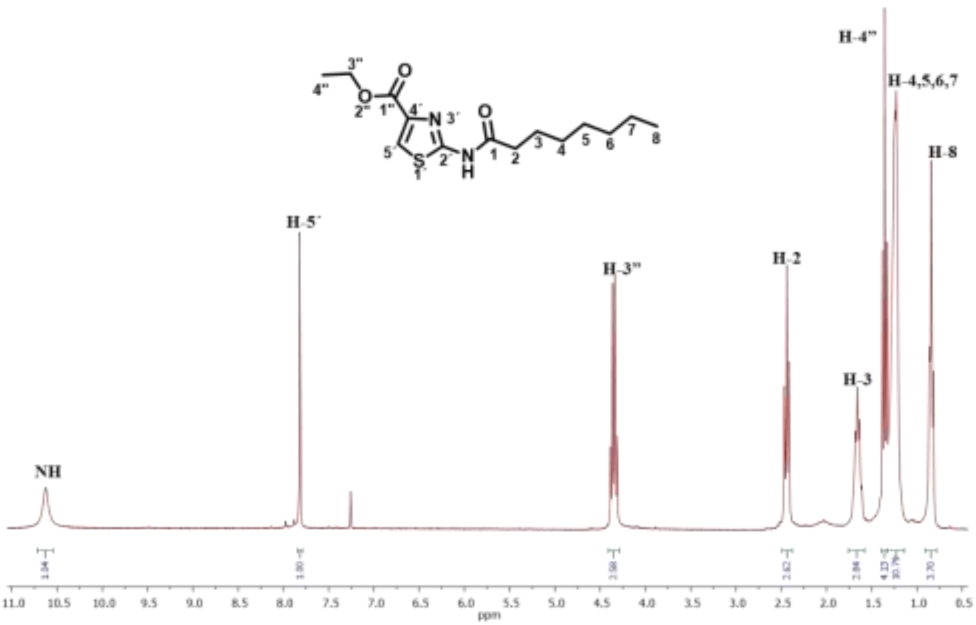

Figure S49. ^{1}H NMR spectrum of compound **16c** (300 MHz, $CDCl_3$).

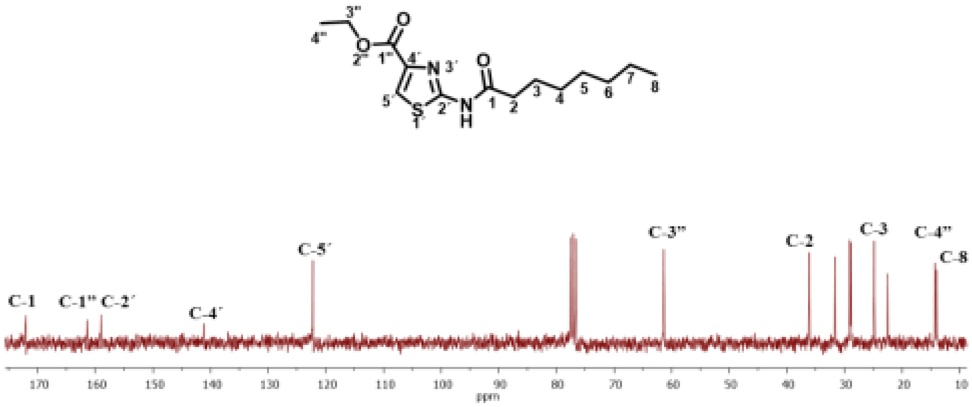

Figure S50. ^{13}C NMR spectrum of compound **16c** (75 MHz, $CDCl_3$).

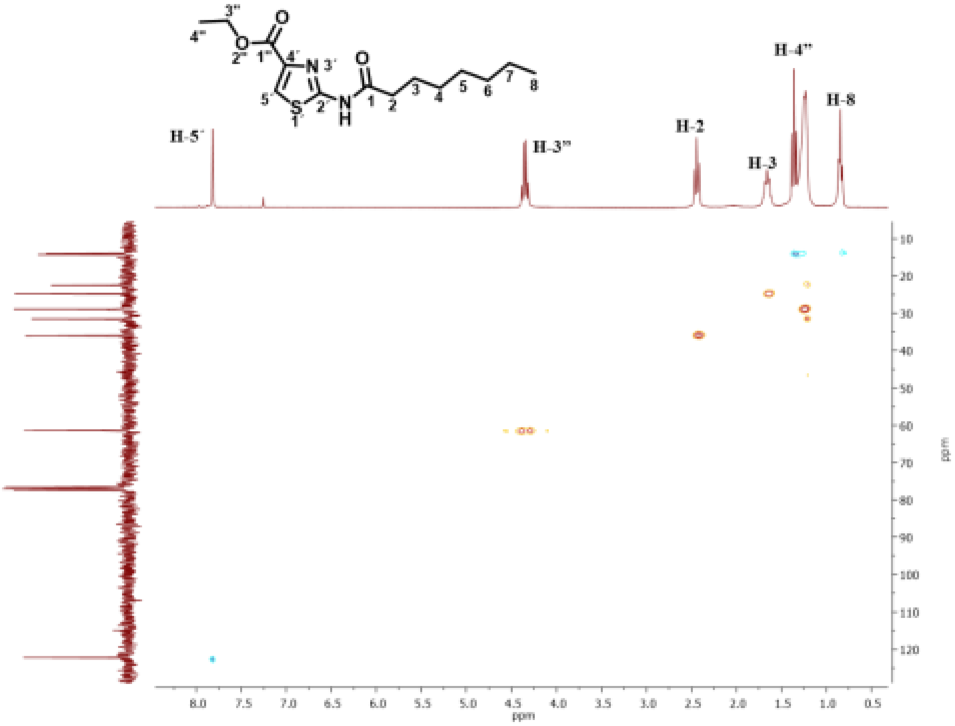

Figure S51. gHSQC spectrum of compound **16c**.

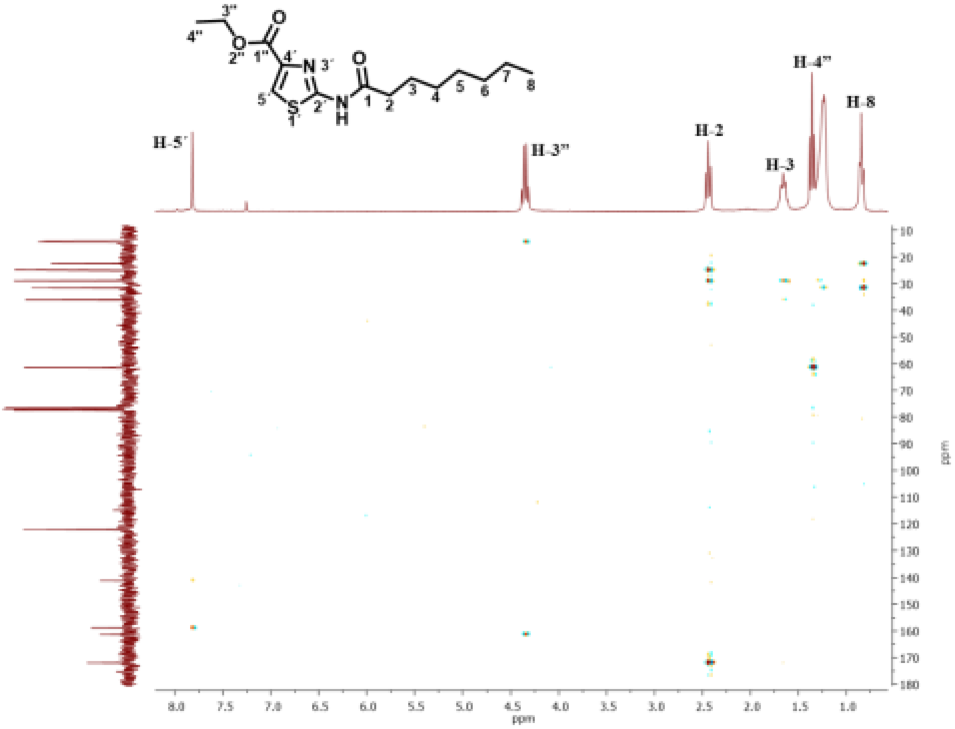

Figure S52. gHMBC spectrum of compound **16c**.

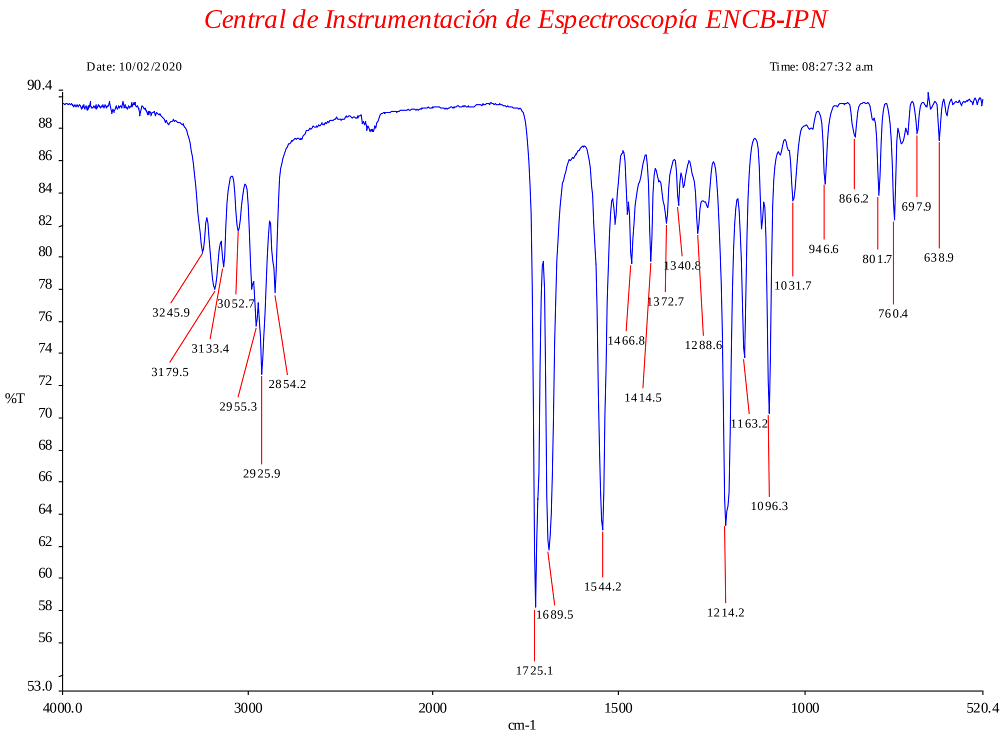

Figure S53. FT-IR spectrum of compound **16c**.

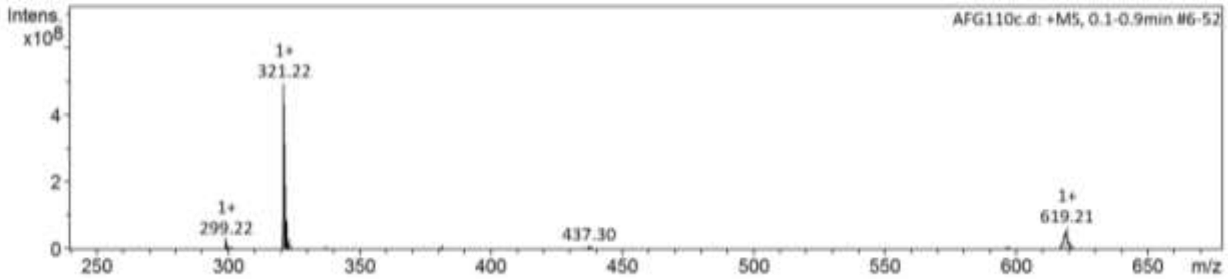

Figure S54. DIP-ESI-MS of compound **16c**.

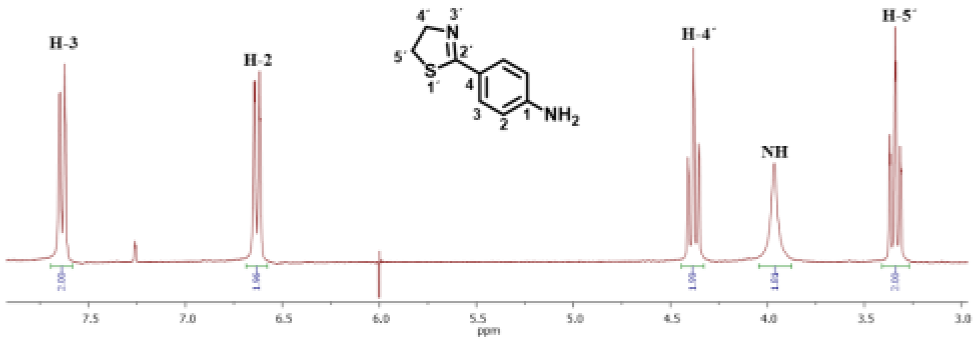

Figure S55. ^{1}H NMR spectrum of compound **25** (300 MHz, $CDCl_3$).

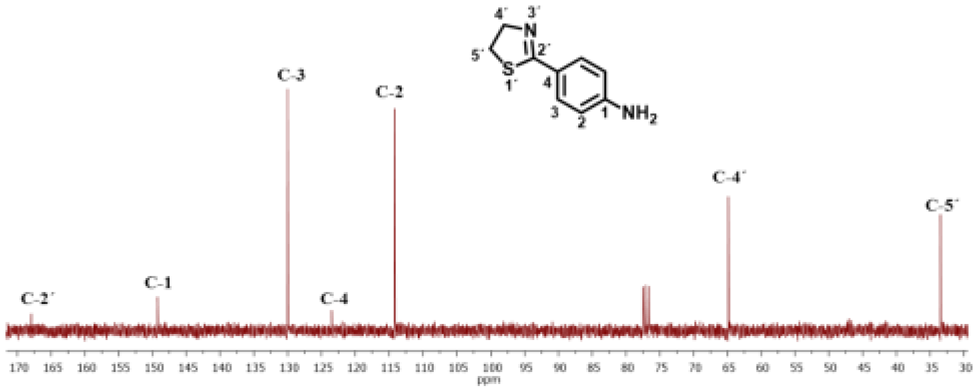

Figure S56. ^{13}C NMR spectrum of compound **25** (75 MHz, $CDCl_3$).

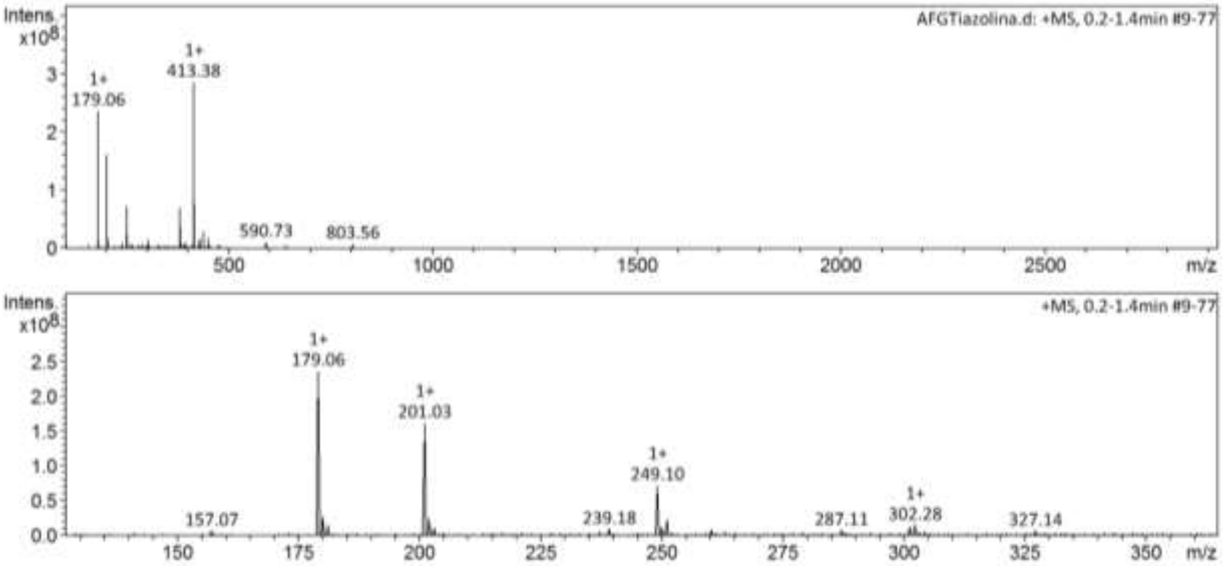

Figure S57. DIP-ESI-MS of compound **25**.

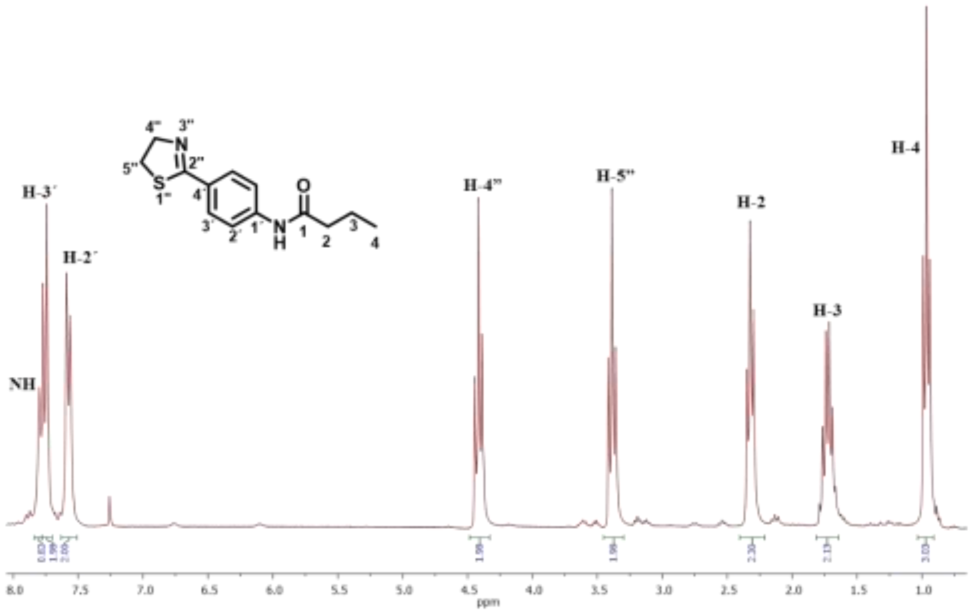

Figure S58. ^{1}H NMR spectrum of compound **17a** (300 MHz, $CDCl_3$).

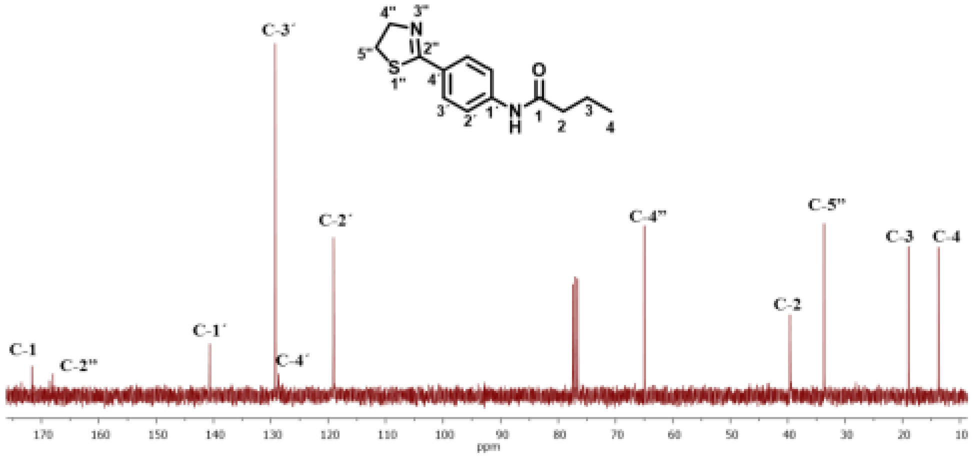

Figure S59. ^{13}C NMR spectrum of compound **17a** (75 MHz, $CDCl_3$).

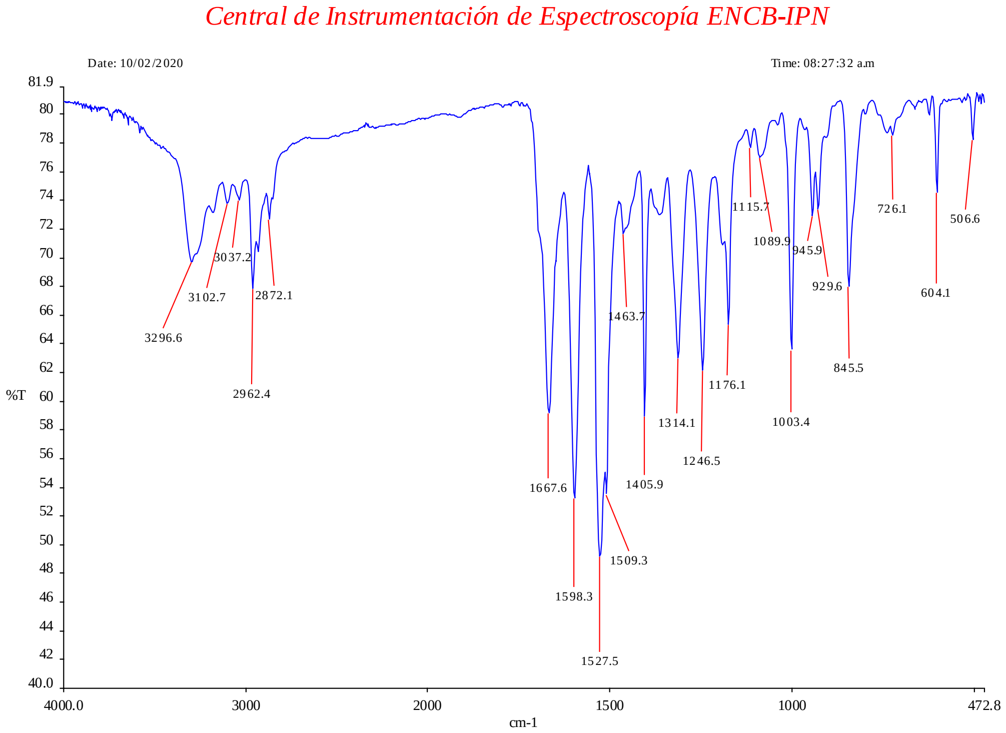

Figure S60. FT-IR spectrum of compound **17a**.

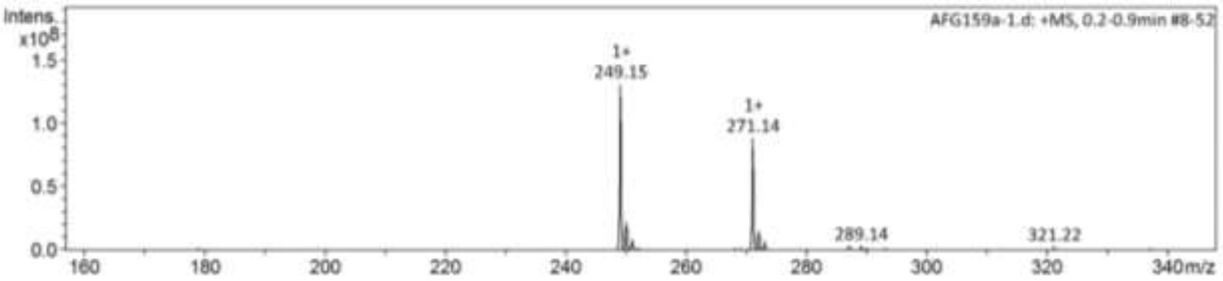

Figure S61. DIP-ESI-MS of compound **17a**.

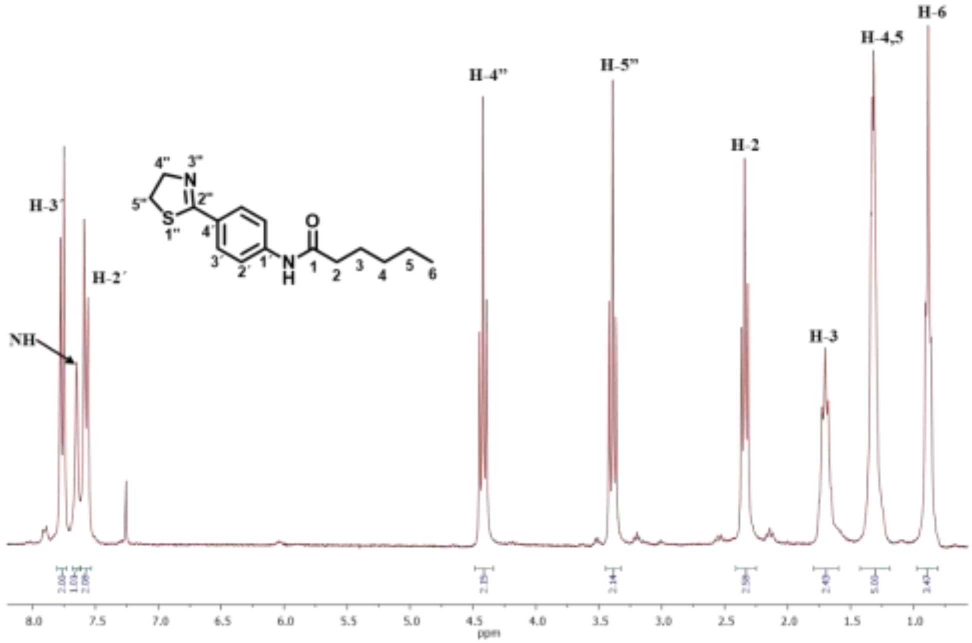

Figure S62. ^{1}H NMR spectrum of compound **17b** (300 MHz, $CDCl_3$).

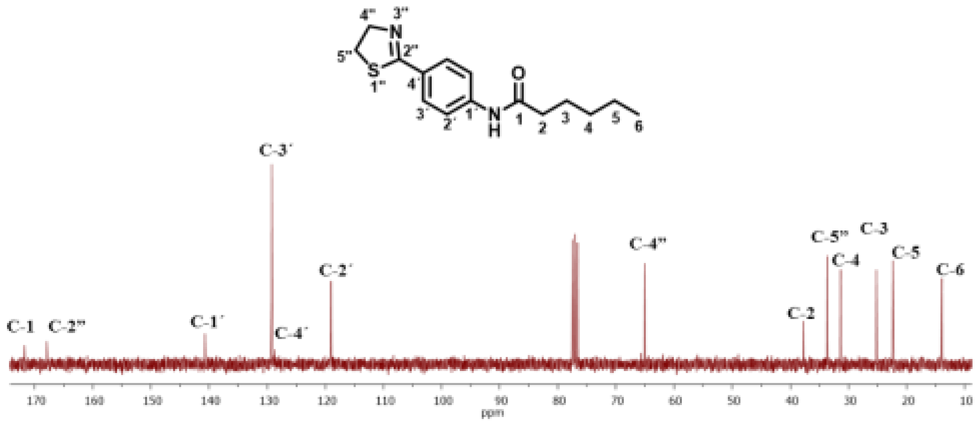

Figure S63. ^{13}C NMR spectrum of compound **17b** (75 MHz, $CDCl_3$).

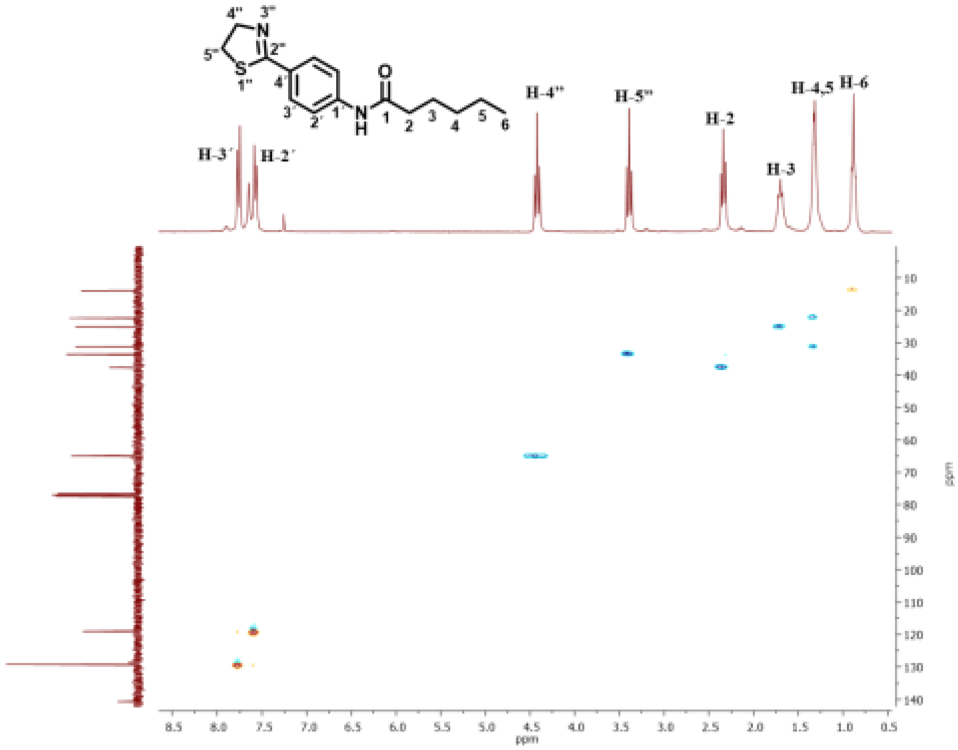

Figure S64. gHSQC spectrum of compound **17b**.

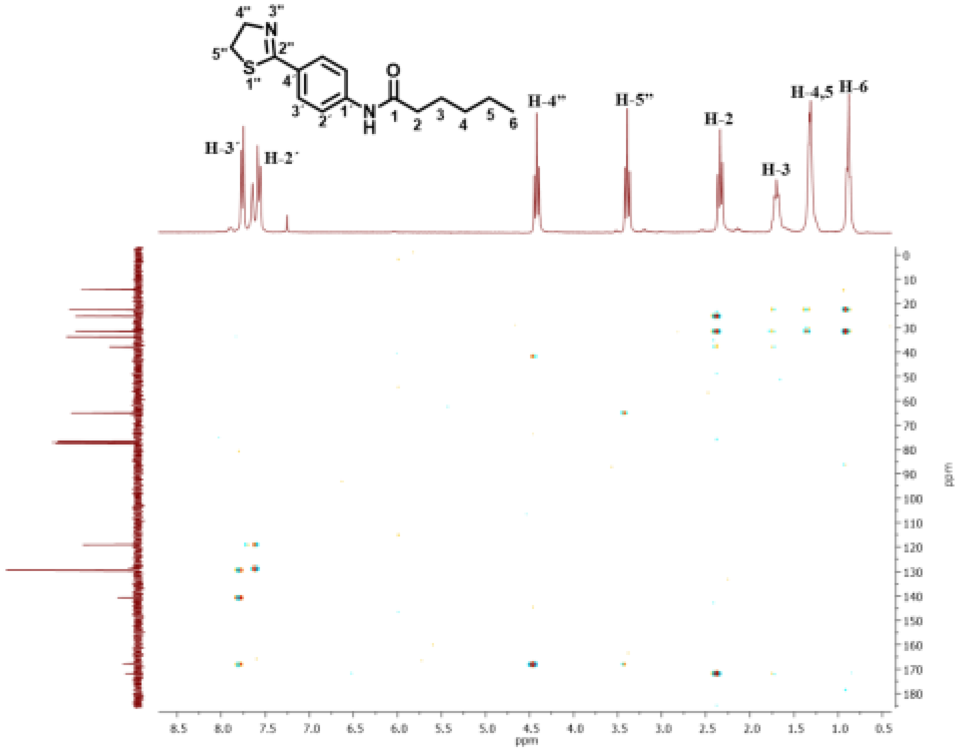

Figure S65. gHMBC spectrum of compound **17b**.

Central de Instrumentación de Espectroscopía ENCB-IPN

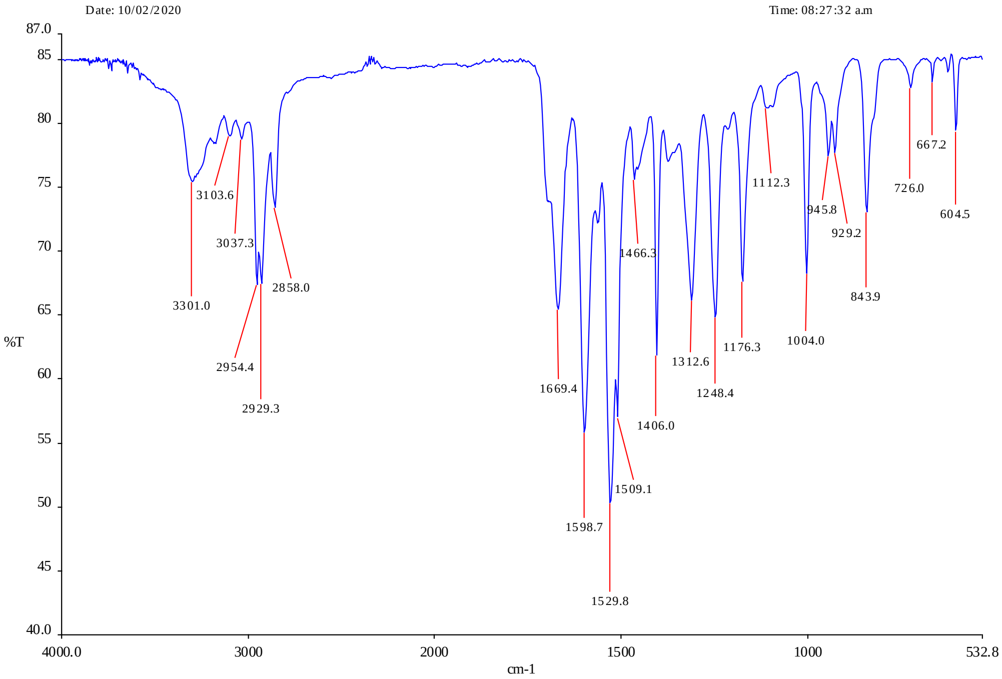

Figure S66. FT-IR spectrum of compound **17b**.

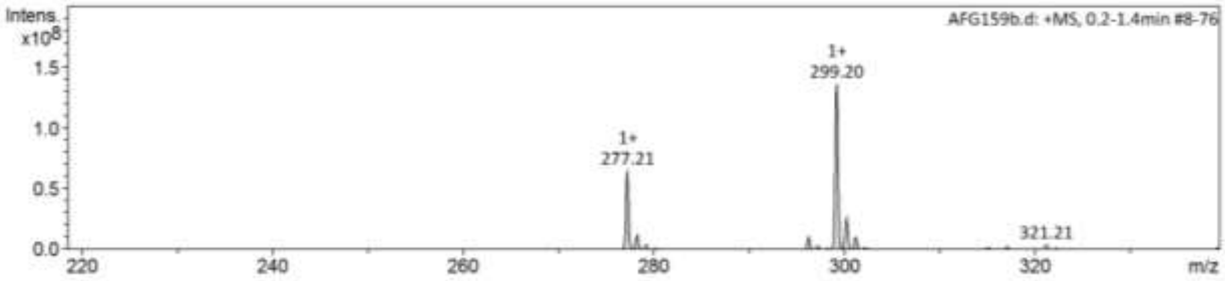

Figure S67. DIP-ESI-MS of compound **17b**.

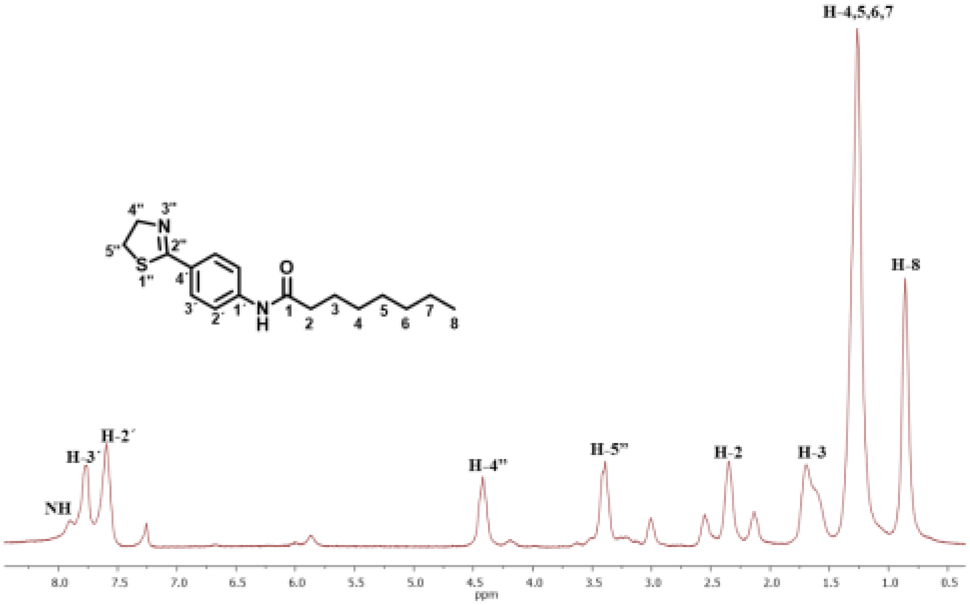

Figure S68. ^{1}H NMR spectrum of compound **17c** (300 MHz, $CDCl_3$).

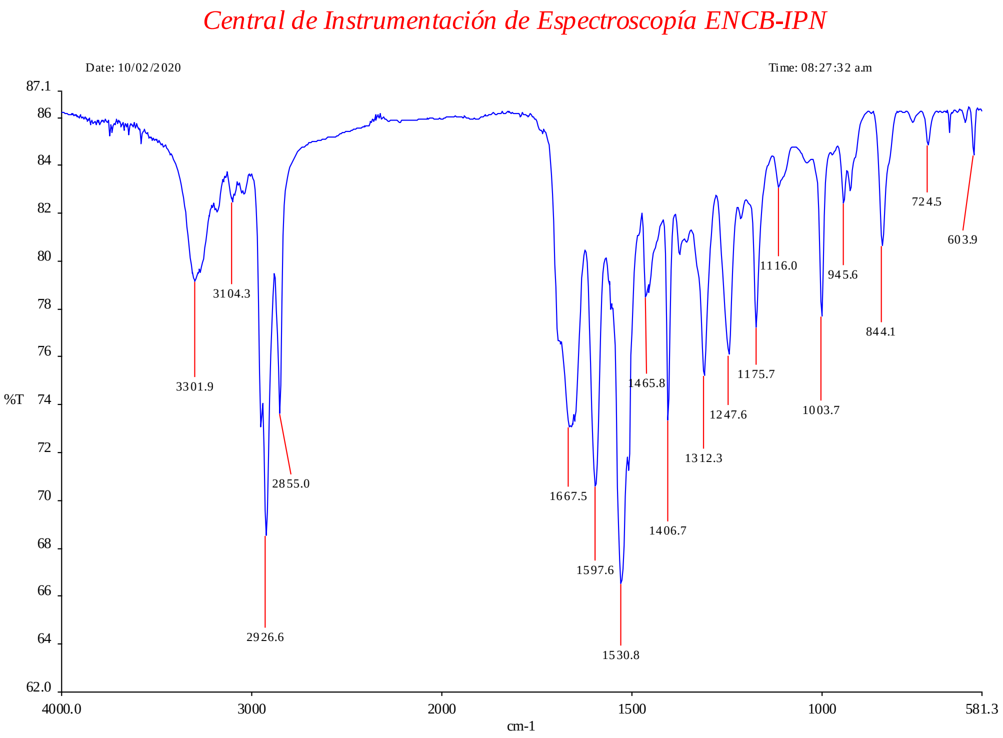

Figure S69. FT-IR spectrum of compound **17c**.

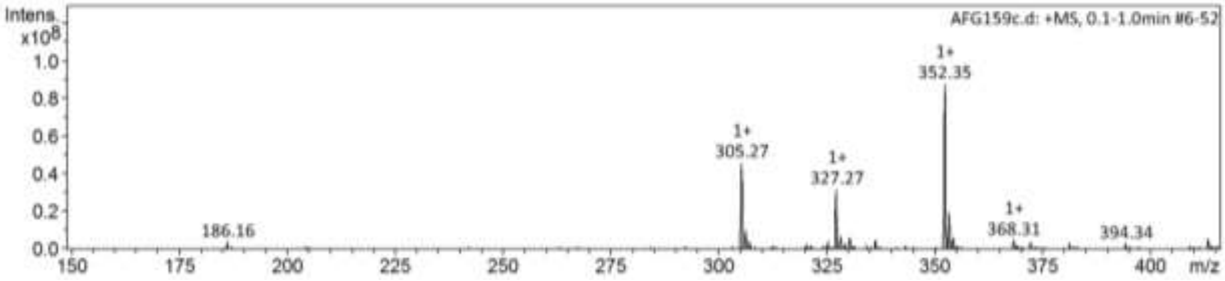

Figure S70. DIP-ESI-MS of compound **17c**.

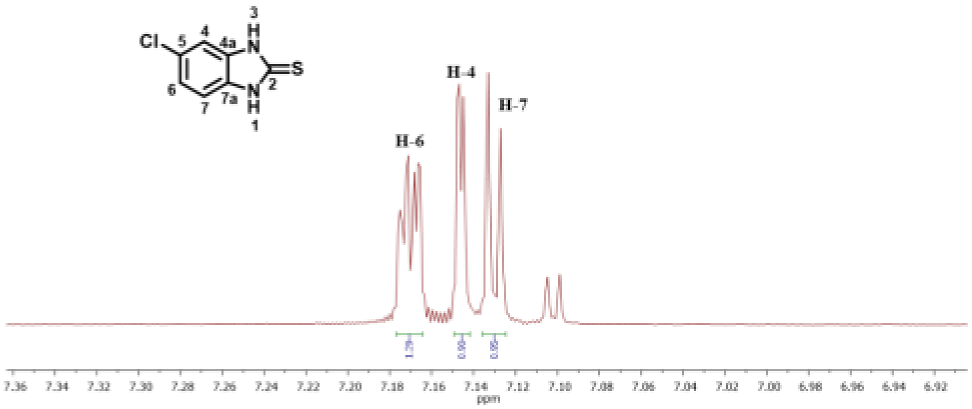

Figure S71. ^{1}H NMR spectrum of compound **27** (300 MHz, Acetona-d_6).

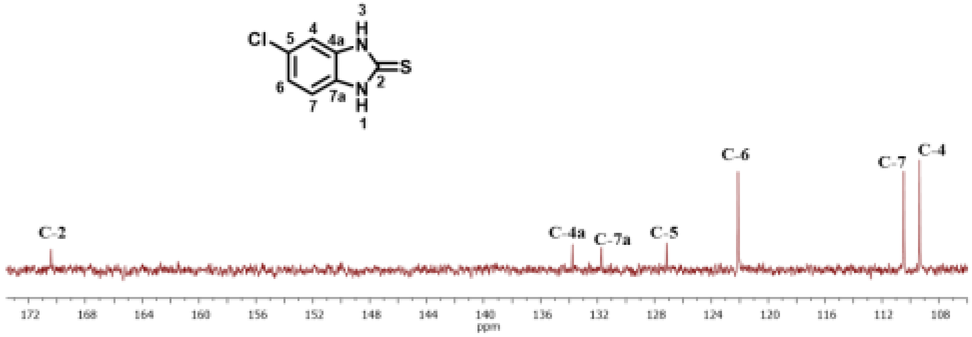

Figure S72. ^{13}C NMR spectrum of compound **27** (75 MHz, Acetona-d_6).

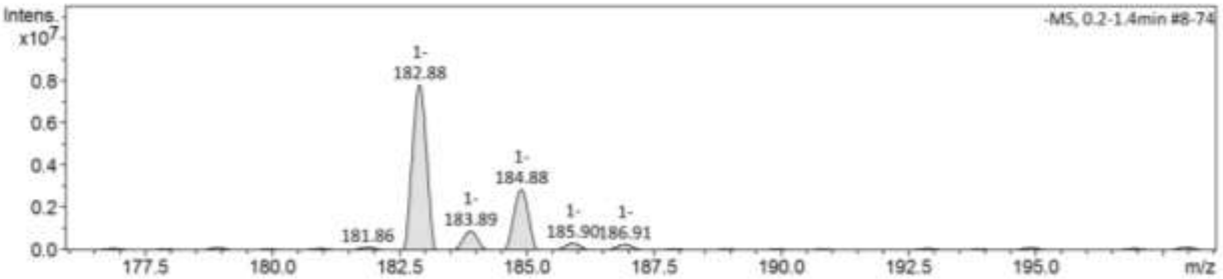

Figure S73. DIP-ESI-MS of compound **27**.

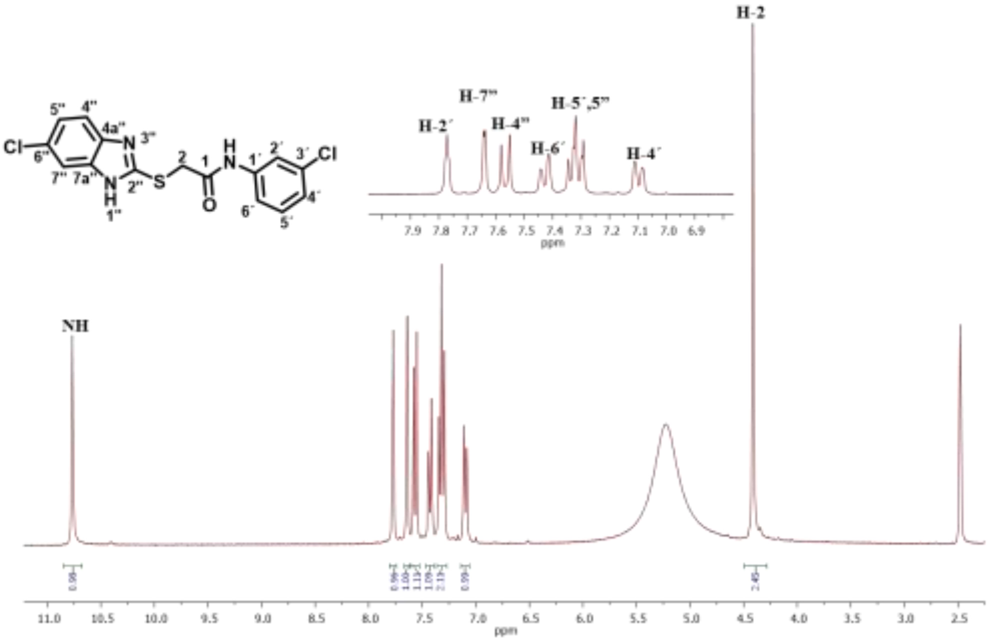

Figure S74. ^{1}H NMR spectrum of compound **18a** (300 MHz, DMSO-d$_6$).

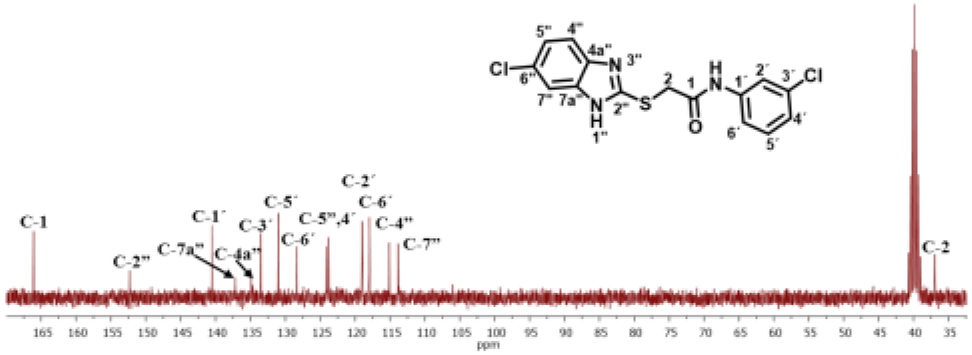

Figure S75. ^{13}C NMR spectrum of compound **18a** (75 MHz, DMSO-d_6).

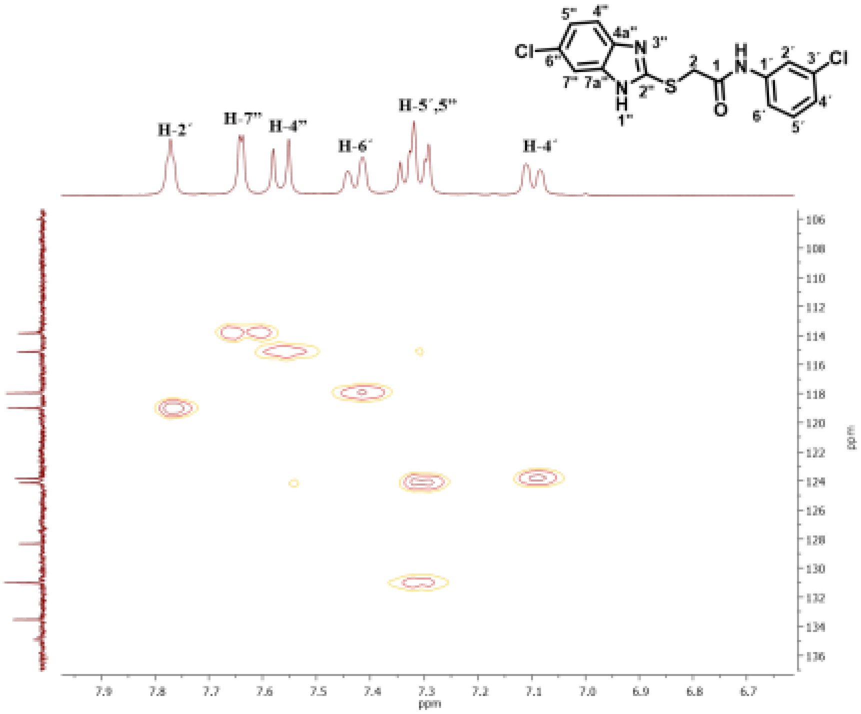

Figure S76. gHSQC spectrum of compound **18a**.

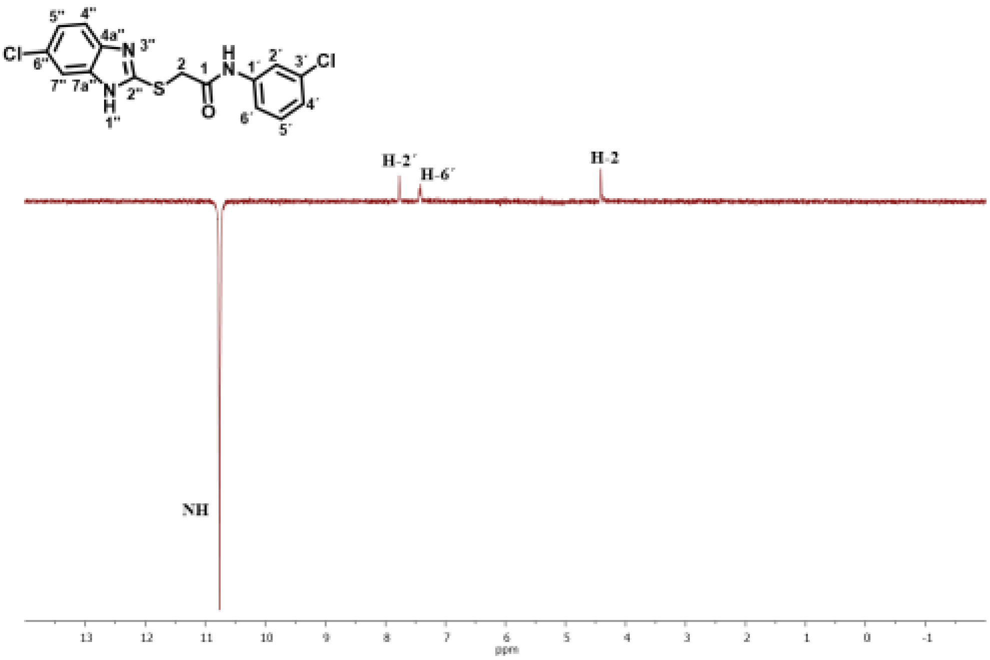

Figure S77. NOESY 1D experiment of compound **18a**.

Central de Instrumentación de Espectroscopía ENCB-IPN

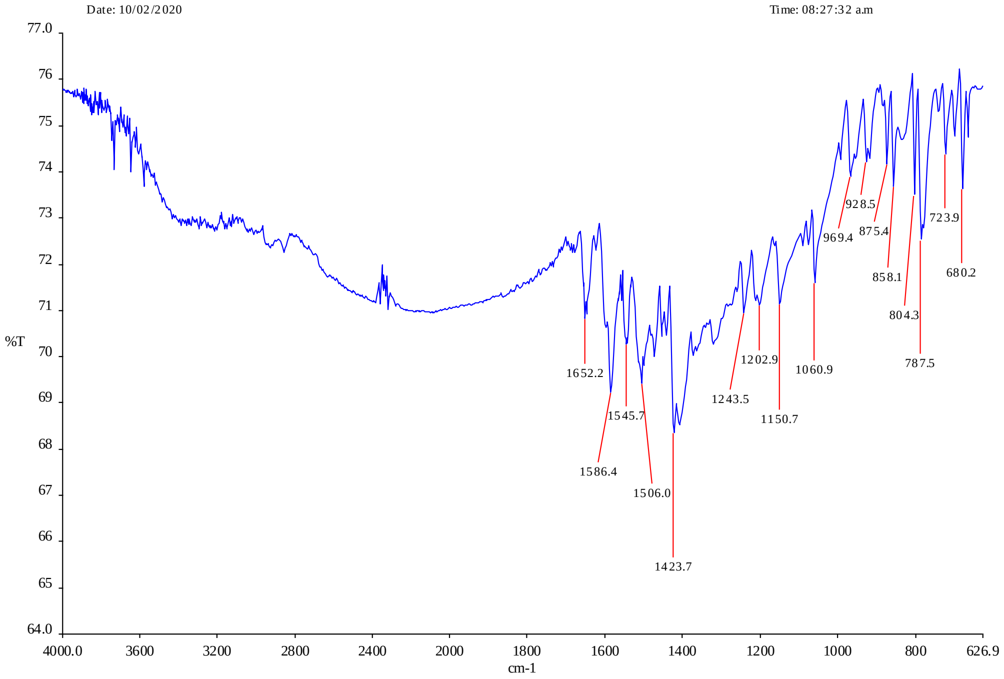

Figure S78. FT-IR spectrum of compound **18a**.

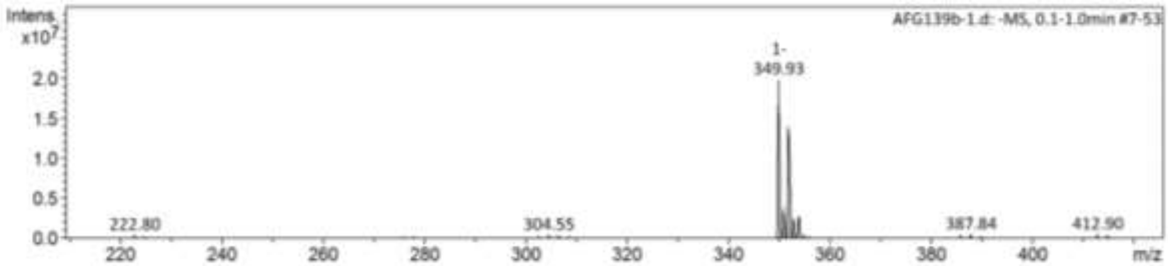

Figure S79. DIP-ESI-MS of compound **18a**.

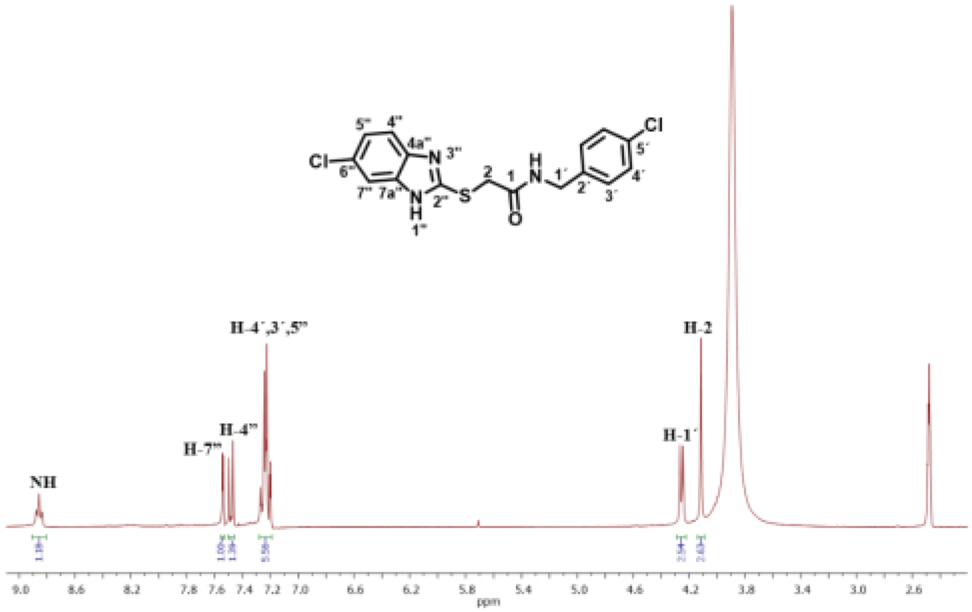

Figure S80. ^{1}H NMR spectrum of compound **18b** (300 MHz, DMSO-d_6).

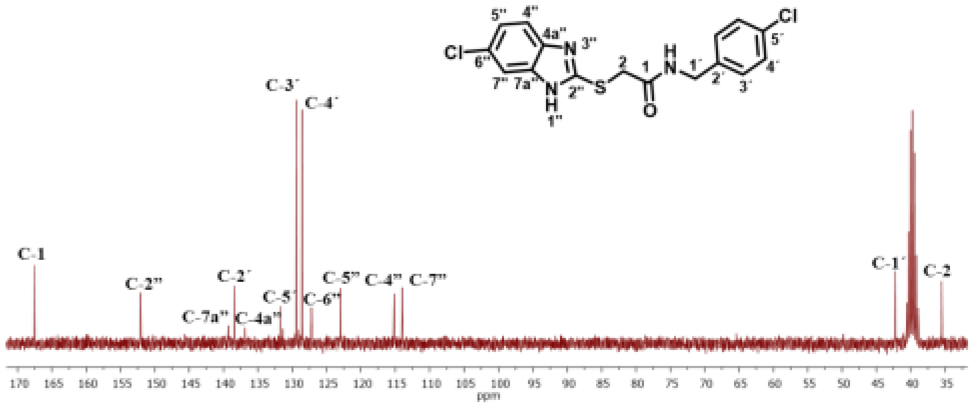

Figure S81. ^{13}C NMR spectrum of compound **18b** (75 MHz, DMSO-d_6).

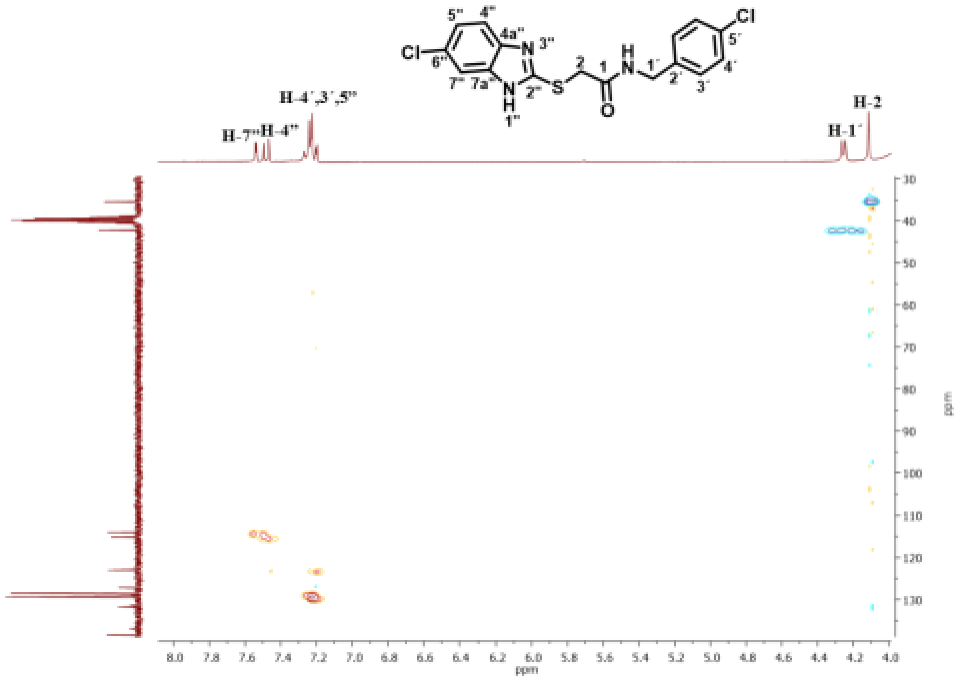

Figure S82. gHSQC spectrum of compound **18b**.

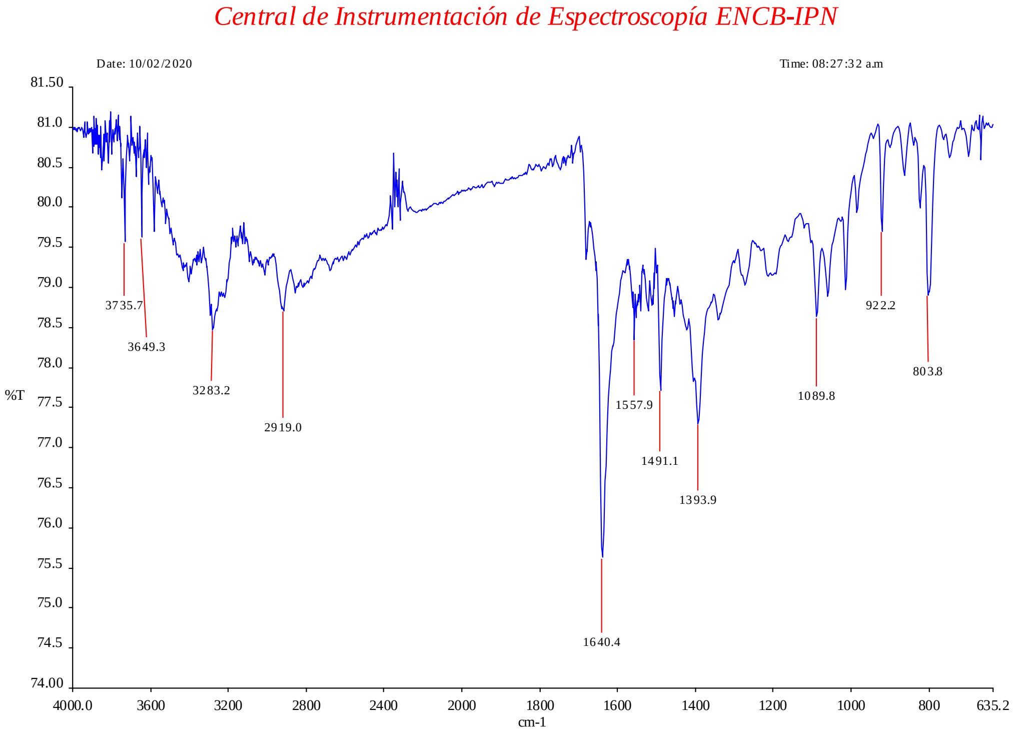

Figure S83. FT-IR spectrum of compound **18b**.

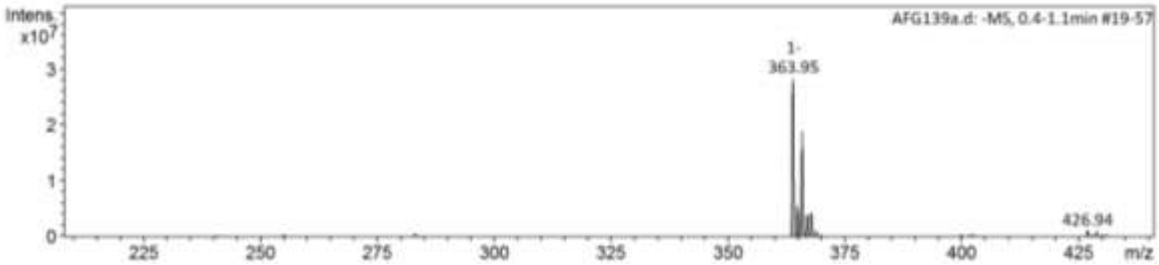

Figure S84. DIP-ESI-MS of compound **18b**.

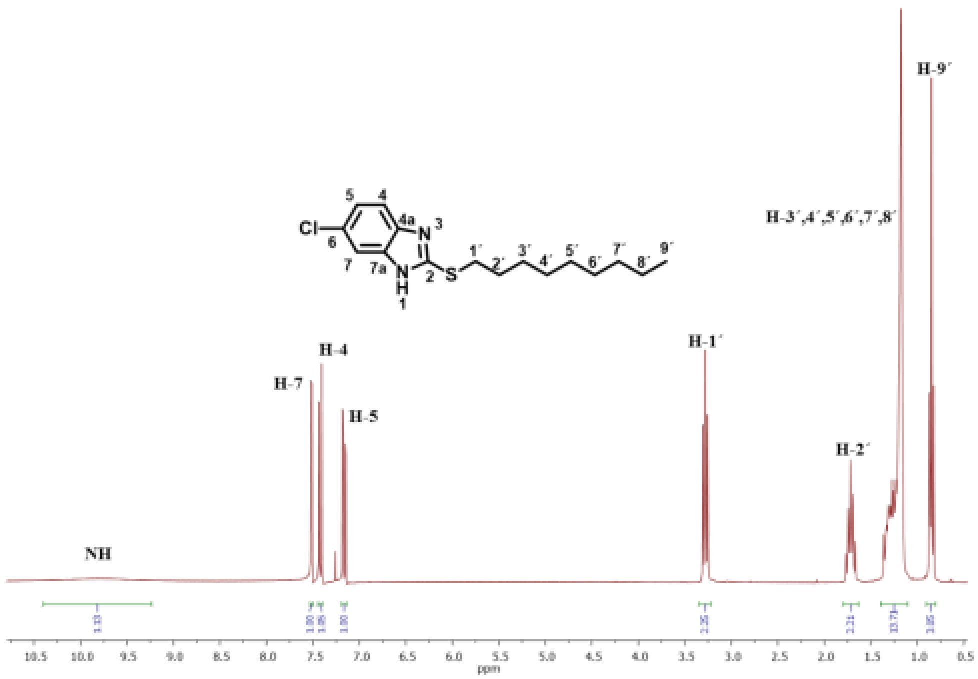

Figure S85. ^{1}H NMR spectrum of compound **18c** (300 MHz, $CDCl_3$).

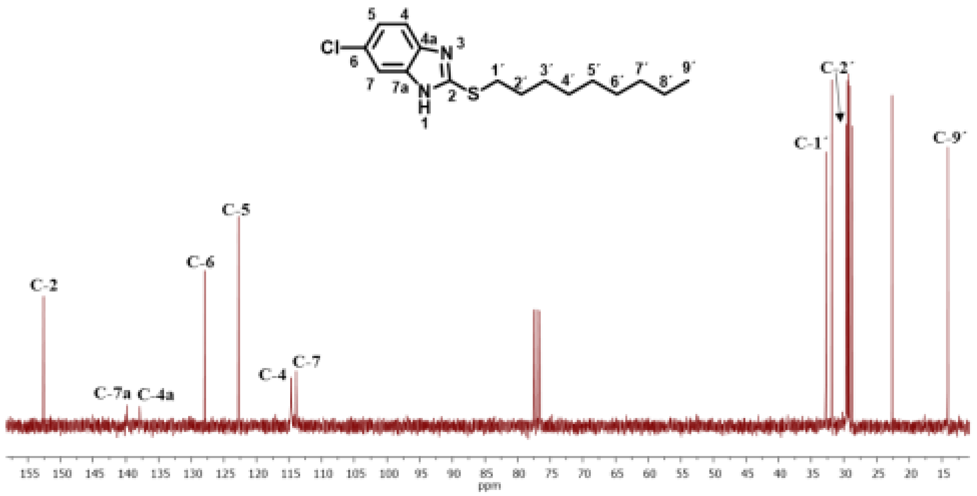

Figure S86. ^{13}C NMR spectrum of compound **18c** (75 MHz, $CDCl_3$).

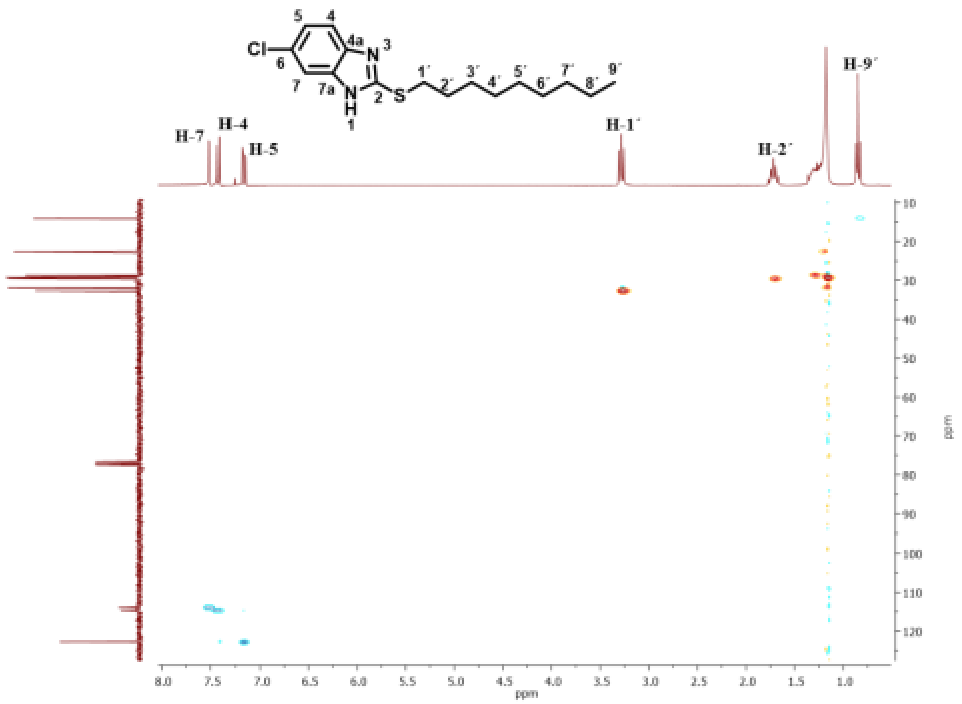

Figure S87. gHSQC spectrum of compound **18c**.

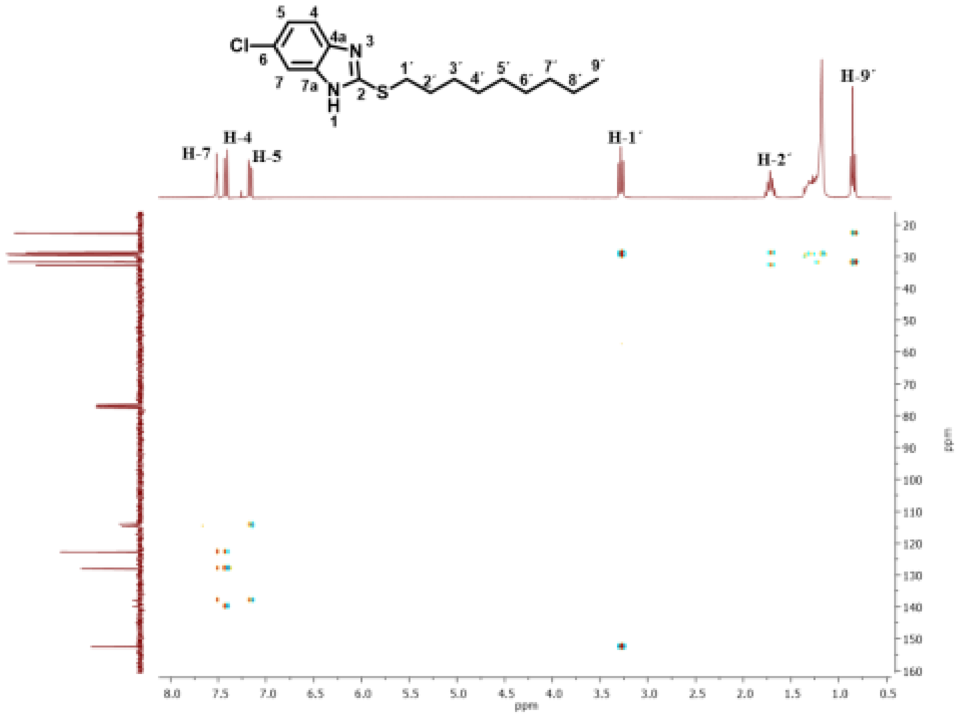

Figure S88. gHMBC spectrum of compound **18c**.

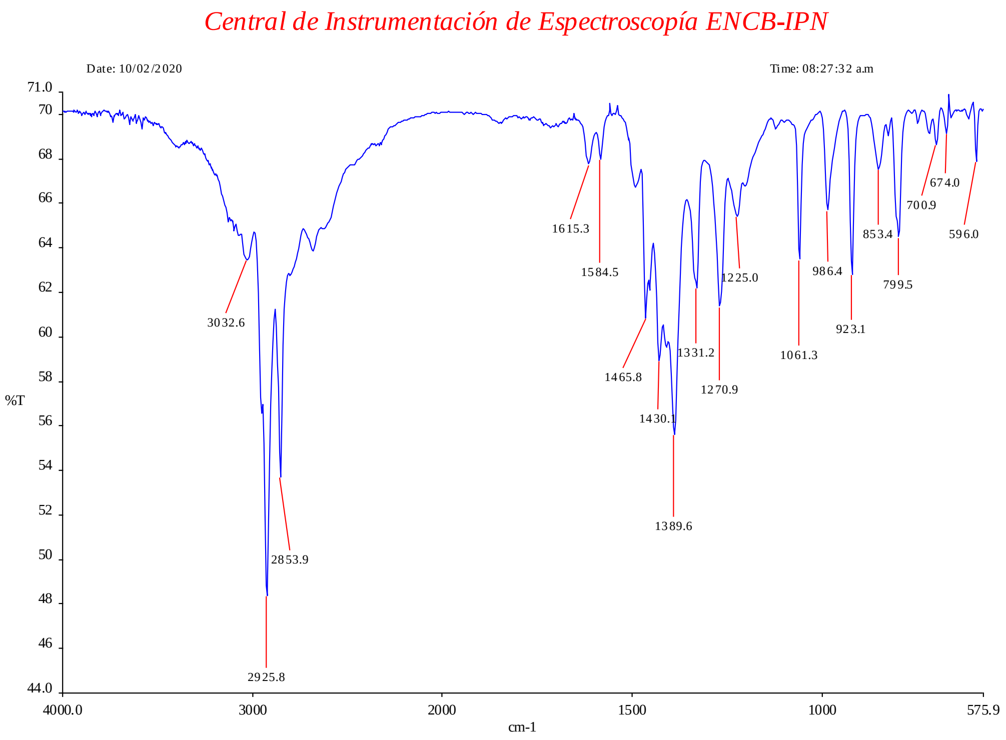

Figure S89. FT-IR spectrum of compound **18c**.

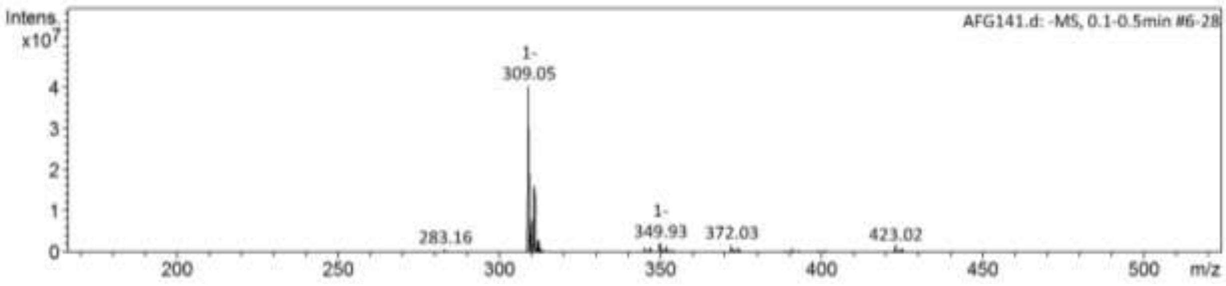

Figure S90. DIP-ESI-MS of compound **18c**.

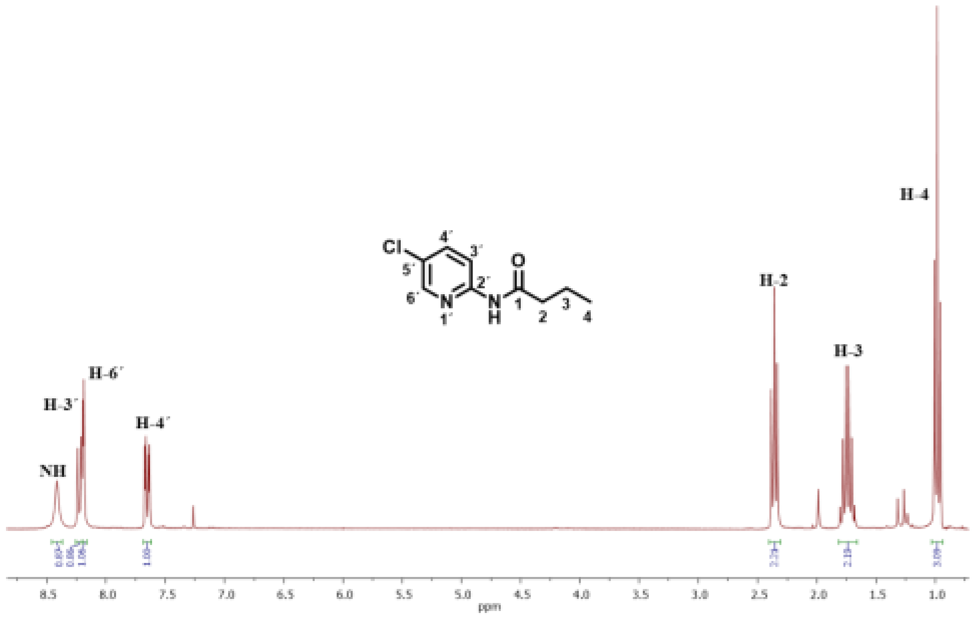

Figure S91. ^{1}H NMR spectrum of compound **19a** (300 MHz, $CDCl_3$).

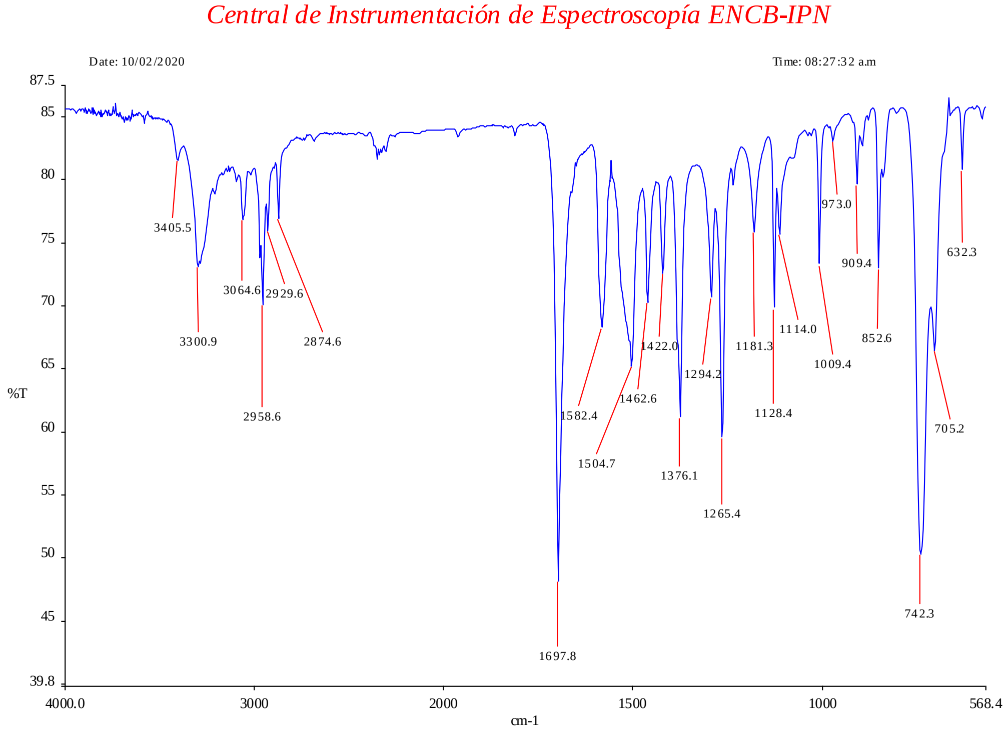

Figure S92. FT-IR spectrum of compound **19a**.

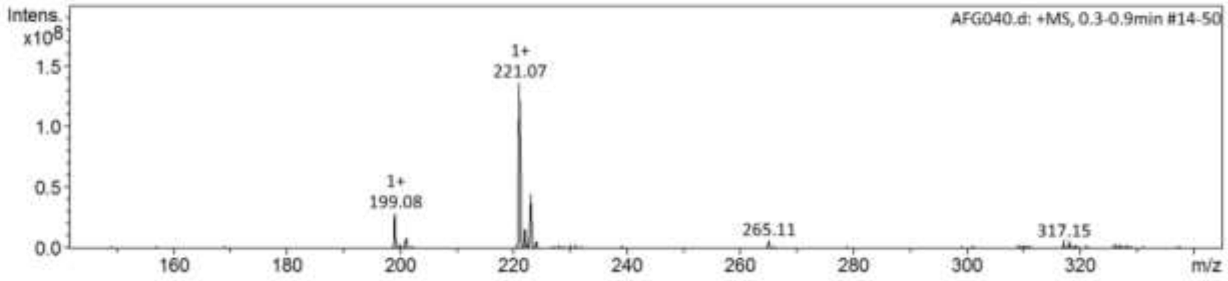

Figure S93. DIP-ESI-MS of compound **19a**.

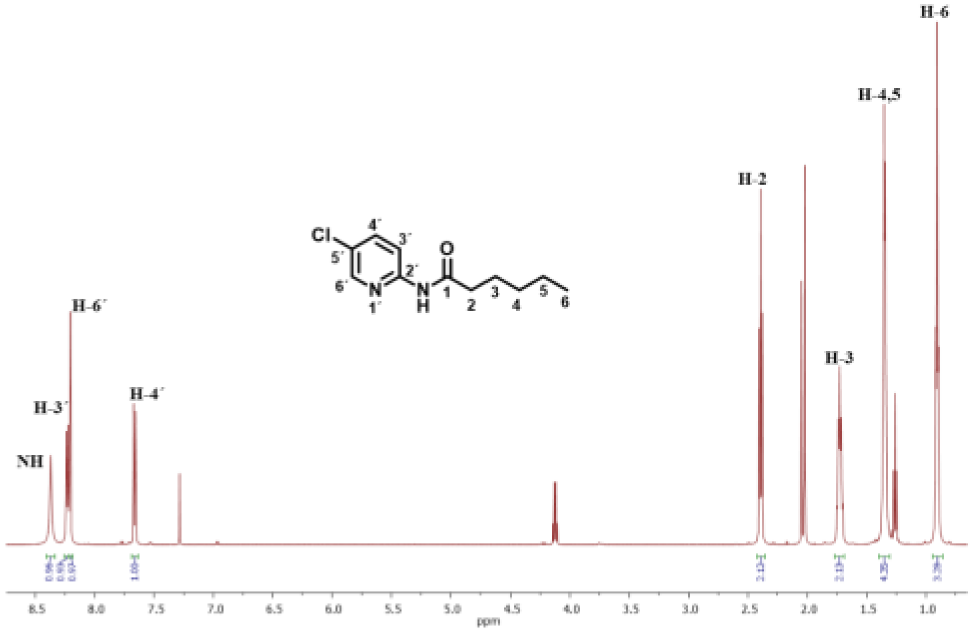

Figure S94. ^{1}H NMR spectrum of compound **19b** (600 MHz, $CDCl_3$).

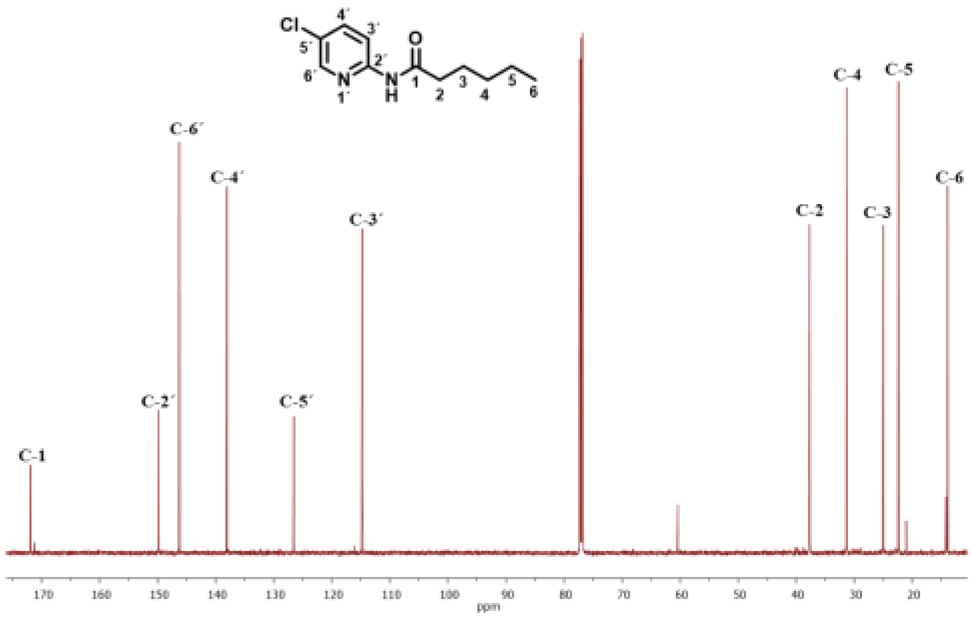

Figure S95. ^{13}C NMR spectrum of compound **19b** (150 MHz, $CDCl_3$).

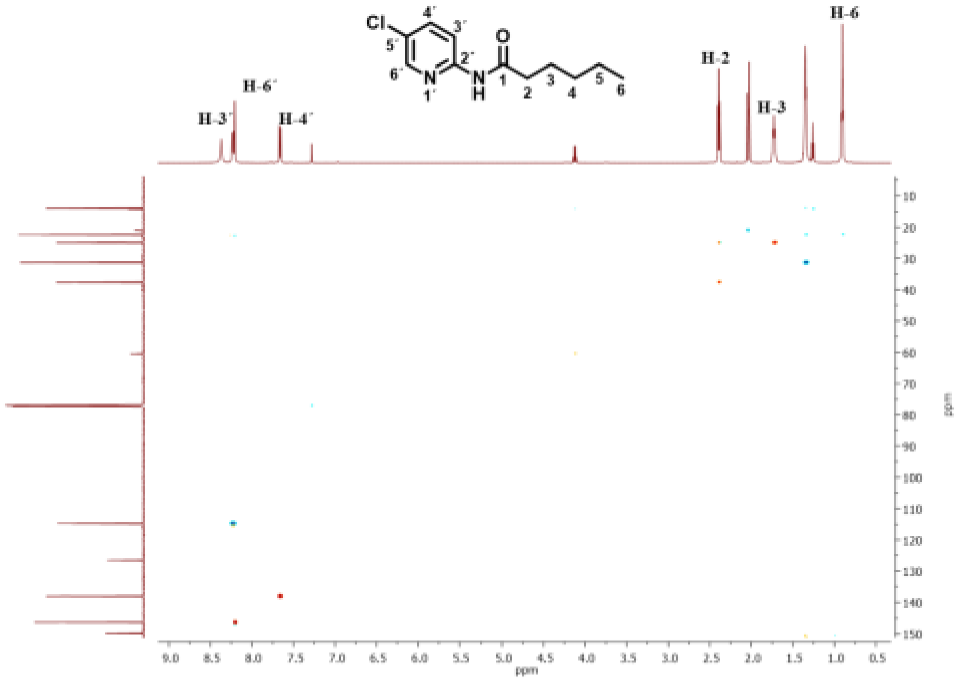

Figure S96. gHSQC spectrum of compound **19b**.

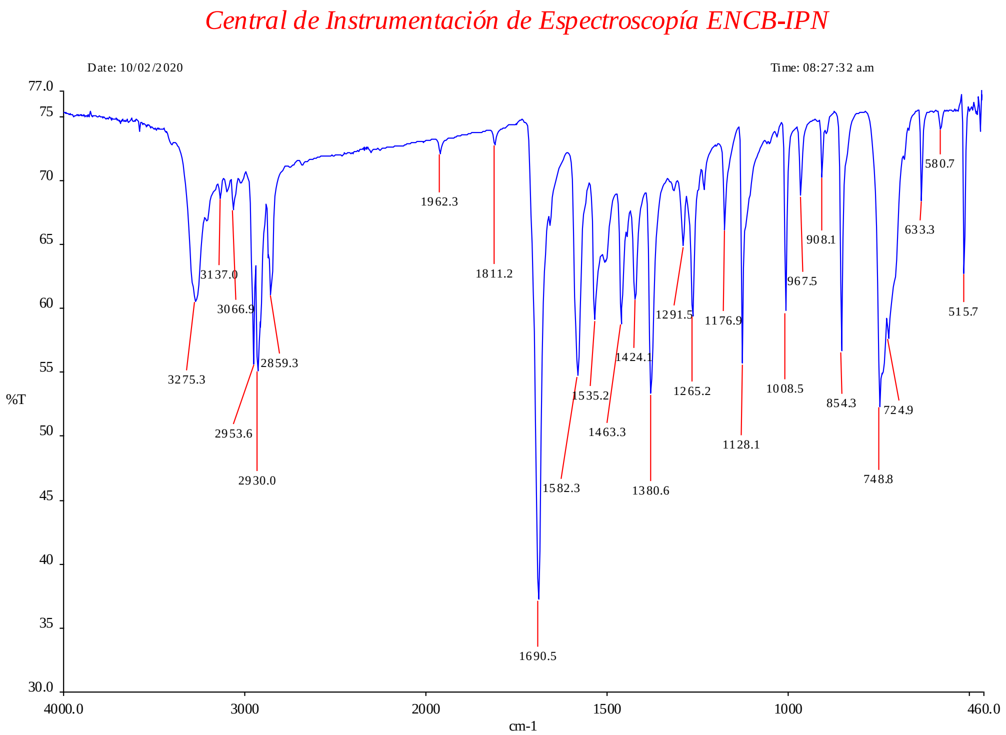

Figure S97. FT-IR spectrum of compound **19b**.

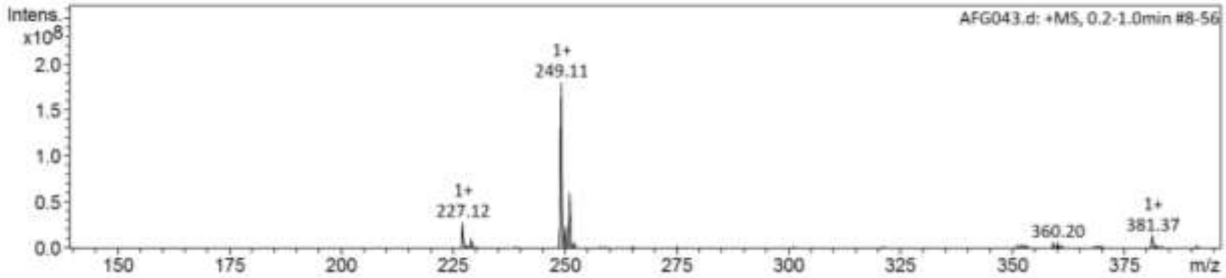

Figure S98. DIP-ESI-MS of compound **19b**.

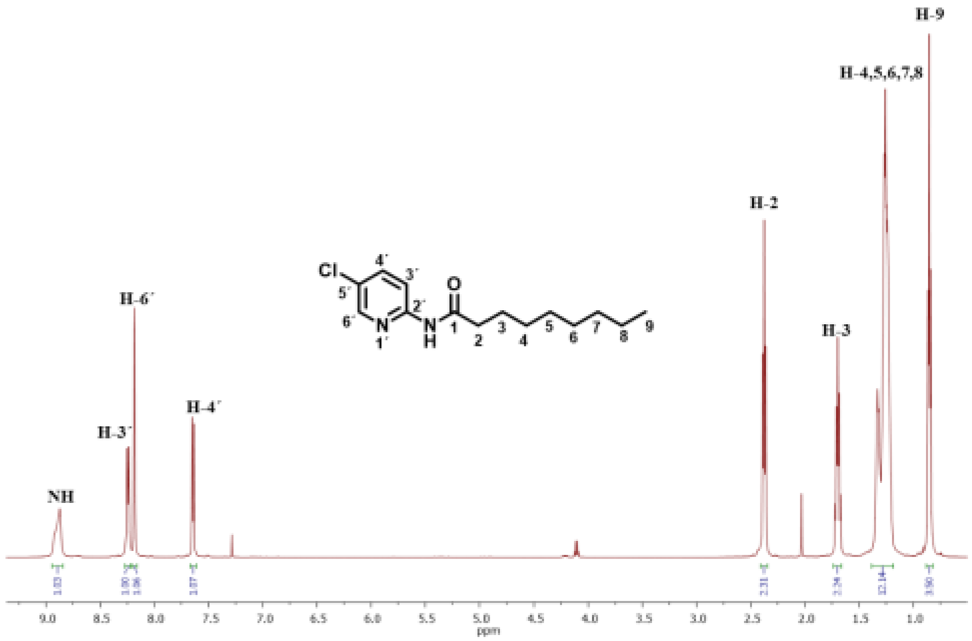

Figure S99. ^{1}H NMR spectrum of compound **19c** (600 MHz, $CDCl_3$).

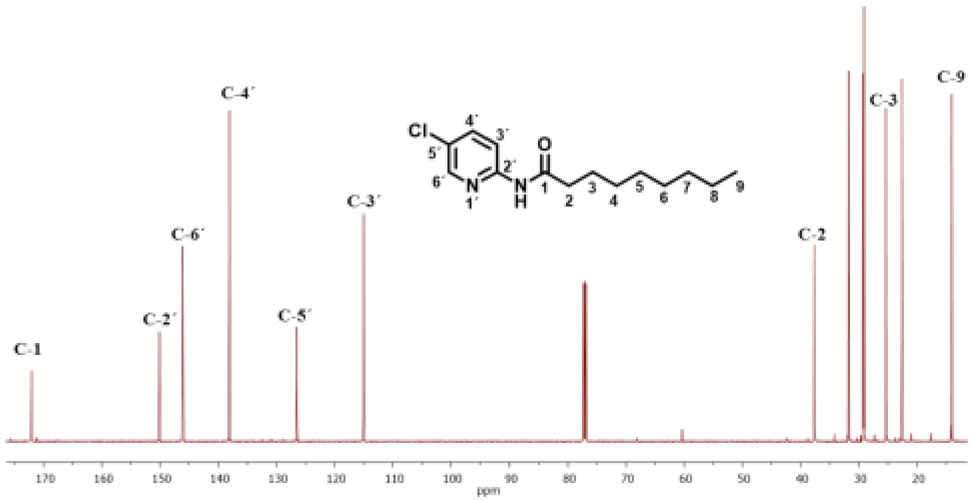

Figure S100. ^{13}C NMR spectrum of compound **19c** (150 MHz, $CDCl_3$).

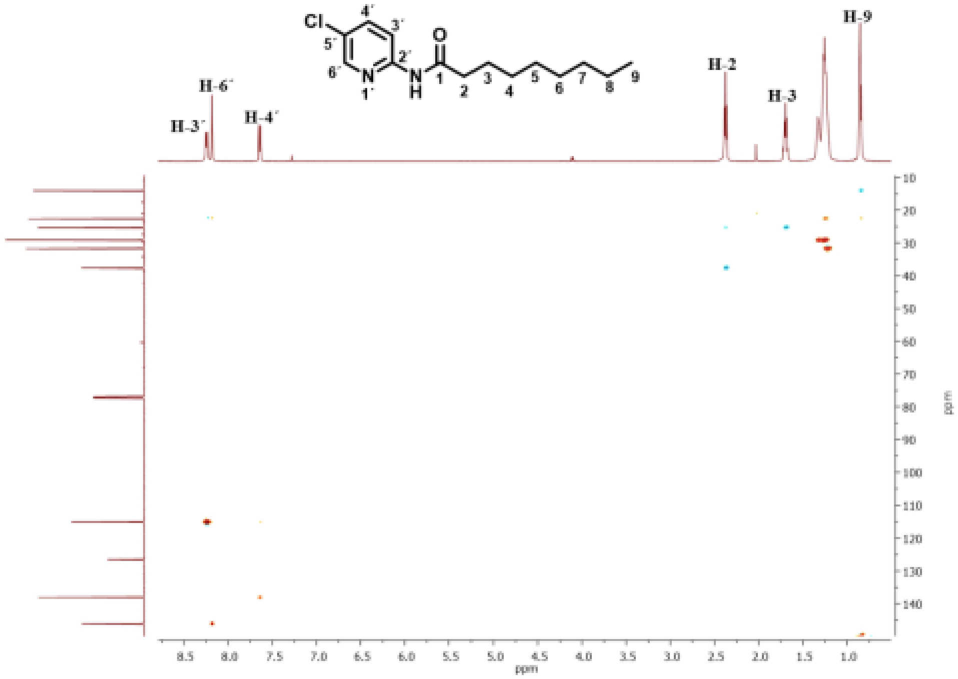

Figure S101. gHSQC spectrum of compound **19c**.

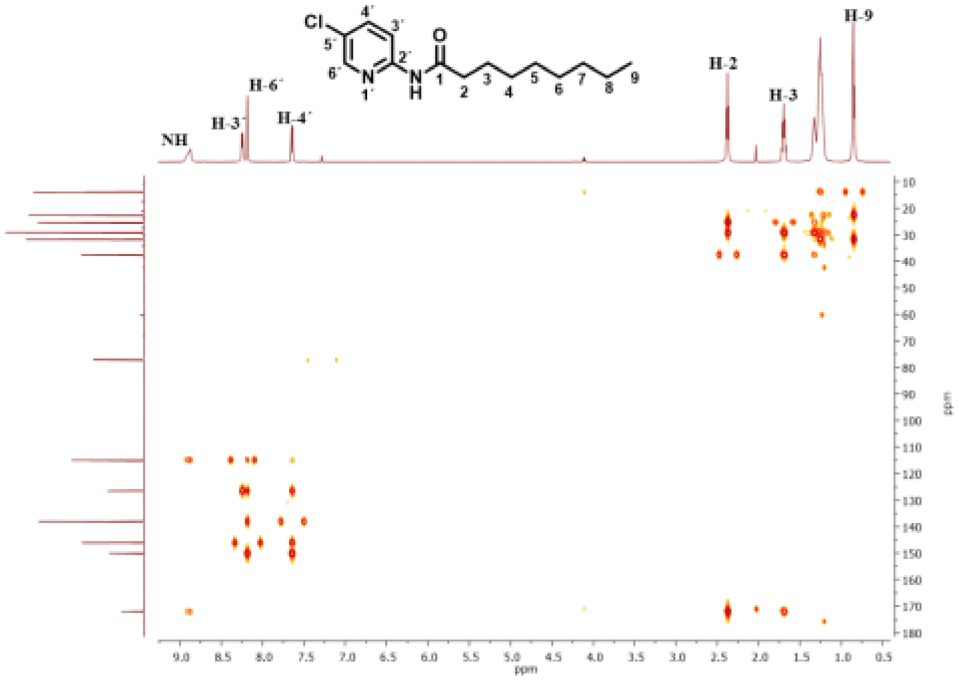

Figure S102. gHMBC spectrum of compound **19c**.

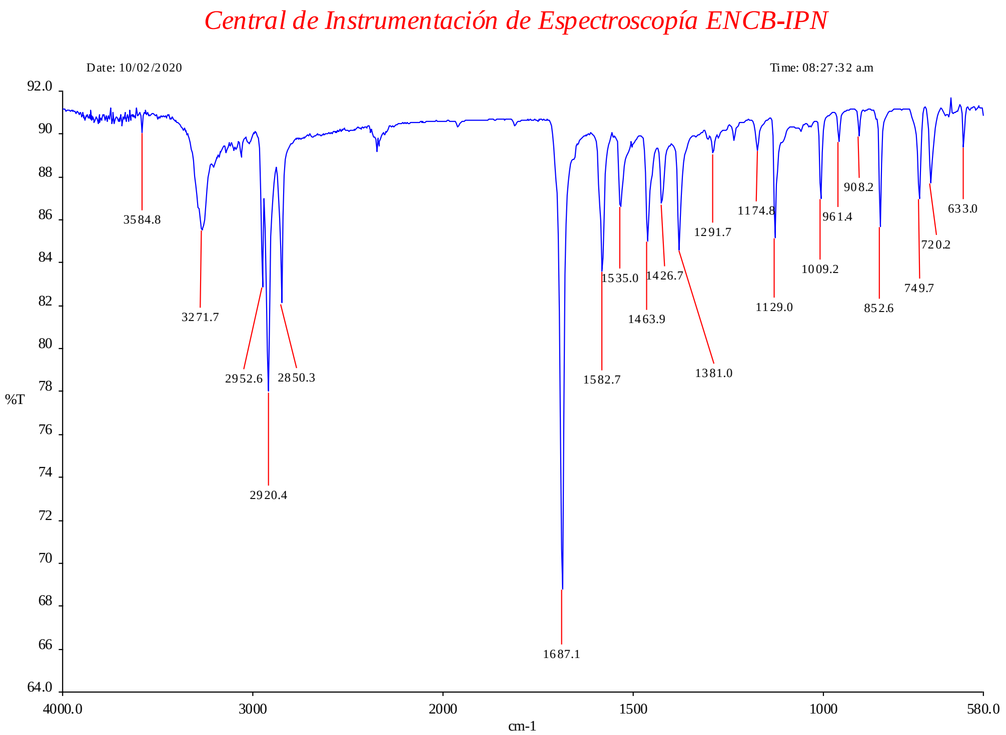

Figure S103. FT-IR spectrum of compound **19c**.

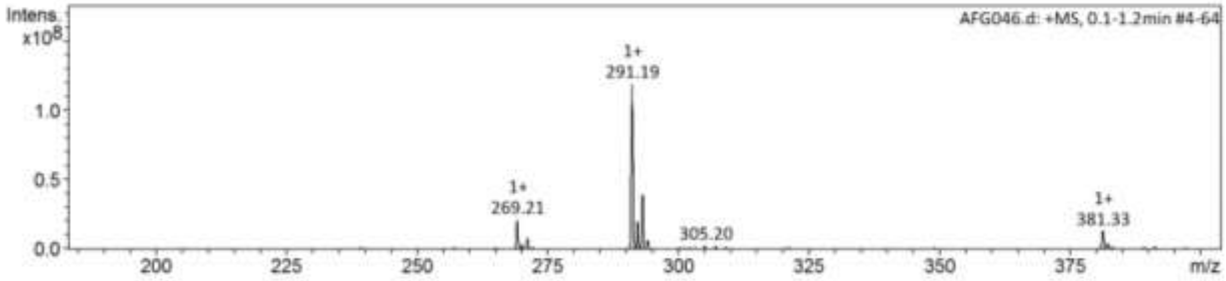

Figure S104. DIP-ESI-MS of compound **19c**.
